# Pan-cancer analysis of telomere maintenance mechanisms

**DOI:** 10.1016/j.jbc.2024.107392

**Published:** 2024-05-18

**Authors:** Meline Hakobyan, Hans Binder, Arsen Arakelyan

**Affiliations:** 1 Bioinformatics Group, Institute of Molecular Biology NAS RA, Yerevan, Armenia; 2 Interdisciplinary Centre for Bioinformatics, University of Leipzig, Leipzig, Germany; 3 Armenian Bioinformatics Institute, Yerevan, Armenia

**Keywords:** cancer, telomere, telomere maintenance mechanisms, telomerase, alternative lengthening of telomeres, pathway signal flow

## Abstract

Telomeres, protective caps at chromosome ends, maintain genomic stability and control cell lifespan. Dysregulated telomere maintenance mechanisms (TMMs) are cancer hallmarks, enabling unchecked cell proliferation. We conducted a pan-cancer evaluation of TMM using RNA sequencing data from The Cancer Genome Atlas for 33 different cancer types and analyzed the activities of telomerase-dependent (TEL) and alternative lengthening of telomeres (ALT) TMM pathways in detail. To further characterize the TMM profiles, we categorized the tumors based on their ALT and TEL TMM pathway activities into five major phenotypes: ALT ^high^ TEL ^low^, ALT ^low^ TEL ^low^, ALT ^middle^ TEL ^middle^, ALT ^high^ TEL ^high^, and ALT ^low^ TEL ^high^. These phenotypes refer to variations in telomere maintenance strategies, shedding light on the heterogeneous nature of telomere regulation in cancer. Moreover, we investigated the clinical implications of TMM phenotypes by examining their associations with clinical characteristics and patient outcomes. Specific TMM profiles were linked to specific survival patterns, emphasizing the potential of TMM profiling as a prognostic indicator and aiding in personalized cancer treatment strategies. Gene ontology analysis of the TMM phenotypes unveiled enriched biological processes associated with cell cycle regulation (both TEL and ALT), DNA replication (TEL), and chromosome dynamics (ALT) showing that telomere maintenance is tightly intertwined with cellular processes governing proliferation and genomic stability. Overall, our study provides an overview of the complexity of transcriptional regulation of telomere maintenance mechanisms in cancer.

Telomeres are unique structures located at the terminal regions of linear chromosomes in eukaryotic cells. In mammals, they consist of repeating DNA sequences, typically composed of the TTAGGG repeats and are associated with a variety of proteins (1). Telomeres play an important role in maintaining the structural integrity of chromosomes and in regulating the replication and stability of the genome (2, 3). With each cell division, the telomeres get shorter and eventually set off an alarm signal in the form of a DNA damage response, causing the cell to either stop dividing or undergo programmed cell death (4). The telomere maintenance mechanism (TMM) is used by cells with unlimited proliferative potential to maintain their telomeres to continue to divide and proliferate (5, 6, 7). The two main TMMs currently known are telomerase-dependent TMM (TEL) (8) and alternative lengthening of telomeres (ALT) (9). TEL-TMM involves the activation of the telomerase enzyme (10). Telomerase is an enzyme that adds telomeric repeating sequences to the ends of chromosomes, thereby preventing telomere shortening (11). The telomerase complex is made up of multiple components, where the core consists of two parts: the telomerase reverse transcriptase (TERT) component which is the catalytic unit, and the telomerase RNA component (TERC) internal telomerase RNA template (12). Mutations or changes to the TERT gene such as modifications to the promoter region (TERTp), amplification of the gene, structural changes to TERT or TERTp, and TERTp hypermethylation can result in increased expression/activity of TERT (13, 14, 15). ALT-TMM uses DNA recombination machinery to extend telomeres. It is associated with several mechanisms such as the presence of heterogeneous telomere lengths, colocalization of telomeres with promyelocytic leukemia nuclear bodies in the ALT-associated promyelocytic leukemia nuclear bodies (called APB), extrachromosomal telomeric repeats such as C-circles, and frequent exchanges between sister telomeres known as T-SCEs. The TMM-ALT is considered more complex compared to telomerase-mediated telomere maintenance, and its detailed mechanism(s) are still under debate (16, 17). Inhibiting telomerase activity is a potential option for cancer therapy because, for most cancers, TEL-TMM activation is considered an important mechanism of cancer progression (18, 19). Nevertheless, recent studies have revealed greater diversity, suggesting that tumors can be characterized not just only by ALT- or TEL-TMM phenotypes (20). It was also found that, in addition to the two main TMMs, some cancer cells may use a combination of TEL and ALT TMMs or switch between them depending on the stage of cancer or the genetics and microenvironmental context (21). For example, some cancer cells may start off using TEL, and then switch to the ALT TMM phenotype as cancer progresses (22). Additionally, a subpopulation of cancer cells within a tumor may use a different TMM than most cells (23). This highlights the need for further research to better understand the diversity and complexity of TMMs used by different types of cancers, and to develop targeted therapies that can effectively counter these mechanisms.

This study proposes a pan-cancer evaluation of telomere length maintenance mechanisms using RNA sequencing data available in The Cancer Genome Atlas (TCGA) to identify similarities and differences among different cancer subtypes. It involves the use of previously developed pathway signatures (24) and the pathway signal flow (PSF) algorithm (25, 26) to estimate the activity levels of TEL and ALT pathways across various cancers. The primary novelty and significance of this study lie in its systematic analysis of the TEL and ALT pathways' activities, and their variations across multiple cancer types. It also delves into the associations between these pathway activities, microsatellite instability status, clinical outcomes, and differential gene expression profiles between distinct TMM phenotypes. Our study unveiled the complex landscape of TMM activities in different cancer types, highlighted associations between TMM pathways, microsatellite instability status, and clinical outcomes, and delineated gene expression patterns associated with distinct TMM phenotypes.

## Results

### Evaluation of telomere maintenance mechanism states across 33 cancer types

To explore the activity of telomere maintenance mechanisms in different types of cancer, we examined RNA-seq data from tumors and matched normal samples from 33 cancer projects, obtained from TCGA dataset (see Table 1). To evaluate the TEL and ALT pathways, we used the TMM assessment approach previously developed in our group (24, 27). It consists of TEL and ALT TMM curated pathways that incorporate gene expression data and topologies of molecular interactions between pathway nodes and the PSF algorithm (25, 26). Specifically, the ALT pathway encompasses 37 genes, while the TEL pathway comprises 26 genes. The TEL pathway is represented through several crucial branches, including TERT activation and recruitment, TERC expression, and dyskerin, culminating in assembling a catalytically active telomerase complex. This complex is subsequently recruited to telomeres and facilitates telomere synthesis by involving telomerase and DNA polymerase alpha in the process (24, 27, 28). On the other hand, the ALT pathway encompasses distinctive branches, including the activation of a DNA damage response, the formation of APBs, and the recruitment of telomeres to APB sites. Following the strand invasion, template-directed synthesis by DNA polymerase delta and processing of Holliday junctions ensure their proper resolution during homologous recombination, which is essential for telomere lengthening in an ALT-dependent manner (29, 30). The PSF algorithm calculates the signal strength that is transmitted from input nodes (genes) to the final sink nodes by considering the interactions among the genes within the pathway using microarray/RNA-seq gene expression fold change (FC) values (see “Experimental procedures” section for details) (25, 26).

**Table 1:** TCGA cancer types with the number of analyzed samples

Cancer type	TCGA code	Number of cancer samples	Number of normal samples	Cancer type	TCGA code	Number of cancer samples	Number of normal samples
Adrenocortical carcinoma	ACC	79	NA^a^	Lung squamous cell carcinoma	LUSC	502	49
Bladder urothelial carcinoma	BLCA	412	19	Mesothelioma	MESO	87	NA^a^
Breast invasive carcinoma	BRCA	1127	99	Ovarian serous cystadenocarcinoma	OV	381	NA^a^
Cervical squamous cell carcinoma and endocervical adenocarcinoma	CESC	306	3	Pancreatic adenocarcinoma	PAAD	179	4
Cholangiocarcinoma	CHOL	35	9	Pheochromocytoma and paraganglioma	PCPG	184	3
Colon adenocarcinoma	COAD	480	41	Prostate adenocarcinoma	PRAD	502	51
Lymphoid Neoplasm Diffuse Large B-cell lymphoma	DLBC	48	NA^a^	Rectum adenocarcinoma	READ	167	10
Esophageal carcinoma	ESCA	163	11	Sarcoma	SARC	263	2
Glioblastoma multiforme	GBM	169	5	Skin cutaneous melanoma	SKCM	472	1
Head and neck squamous cell carcinoma	HNSC	504	44	Stomach adenocarcinoma	STAD	375	32
Kidney chromophobe	KICH	65	25	Testicular germ cell tumors	TGCT	156	NA^a^
Kidney renal clear cell carcinoma	KIRC	541	72	Thymoma	THYM	120	2
Kidney renal papillary cell carcinoma	KIRP	291	32	Thyroid carcinoma	THCA	514	57
Acute myeloid leukemia	LAML	150	NA^a^	Uterine carcinosarcoma	UCS	57	NA
Brain lower grade glioma	LGG	532	NA^a^	Uterine corpus endometrial carcinoma	UCEC	554	35
Liver hepatocellular carcinoma	LIHC	374	50	Uveal melanoma	UVM	80	NA^a^
Lung adenocarcinoma	LUAD	540	58				

a Note: "NA" indicates Not available data in the TCGA.

**Figure 1:**
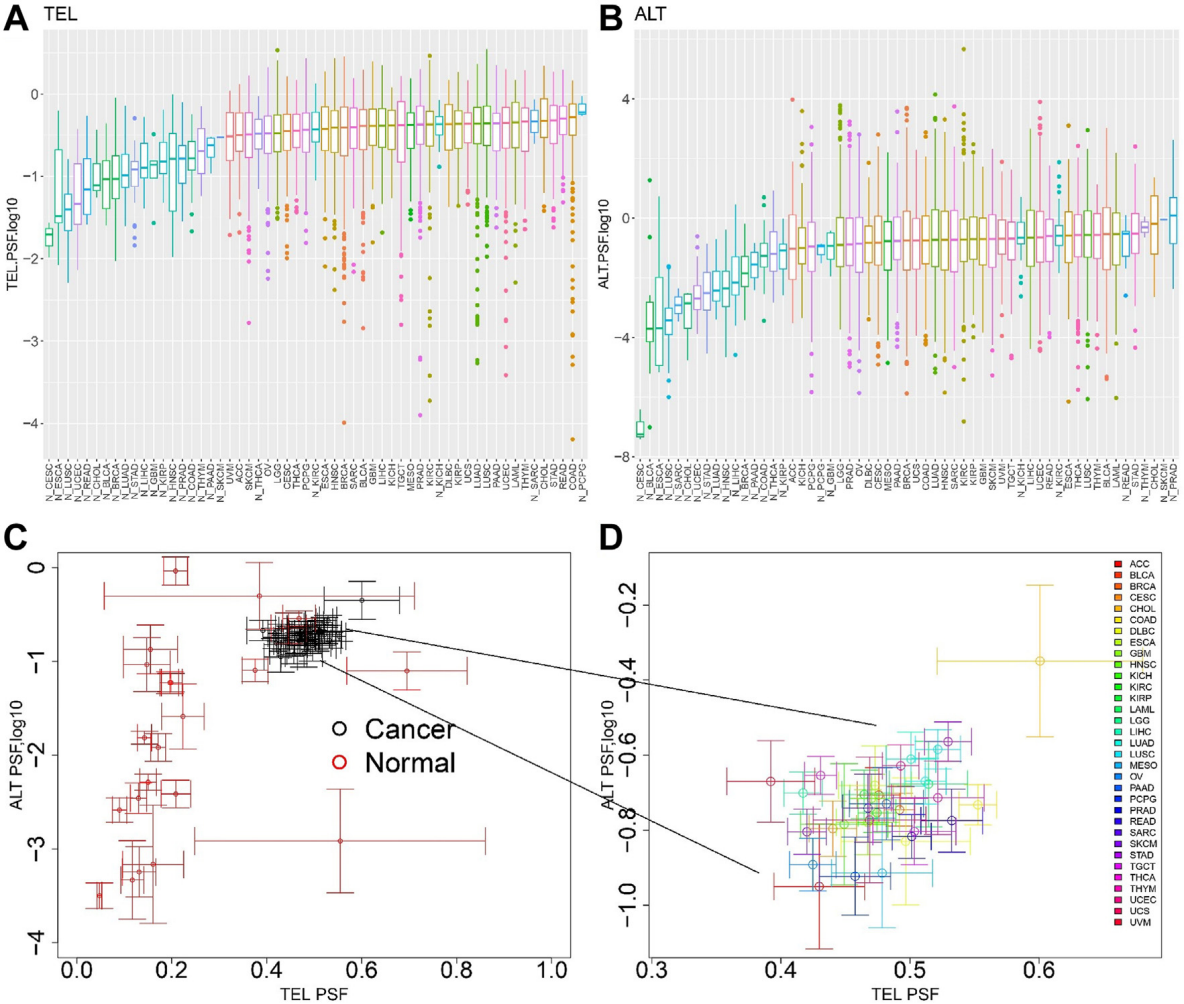
**Comparison of ALT and TEL pathway activity in cancer and normal tissues.** ALT and TEL pathway activity plots (*A*) TEL and (*B*) ALT pathway activity for 33 cancer types and matched normal tissues (prefix N_) plotted according to the TEL (log10) PSF and ALT (log10) PSF and ordered according to median values. *C*, ALT (log10) PSF *versus* TEL (PSF) biplot of mean values and standard error bars in cancerous (*black*) and normal (*red*) samples. *D*, zoom in for 33 cancer types. ALT, alternative lengthening of telomeres; PSF, pathway signal flow; TEL, telomerase-dependent.

Based on the PSF score, which serves as an estimate of the overall activity of the TEL and ALT pathways, the TCGA studies were ordered according to the median of the PSF value (Fig. 1, *A* and *B*). The highest median PSF values for the TEL TMM pathway were observed in colon adenocarcinoma (COAD), rectum adenocarcinoma (READ), stomach adenocarcinoma (STAD) cancer cells, as well as in normal tumor-adjacent cells of pheochromocytoma and paraganglioma. For the ALT pathway, higher activity was noticed in the normal tumor-adjacent samples of prostate adenocarcinoma (PRAD), skin cutaneous melanoma (SKCM), and cancer cells of cholangiocarcinoma (CHOL), and STAD. Our data suggest that ALT and TEL activity in normal tissues were lower compared to cancers in the majority of TCGA cancer types. The most notable difference between matched normal samples and cancer samples was for cervical squamous cell carcinoma and endocervical adenocarcinoma. Moreover, the ALT pathway exhibited greater variability across different cancer types (Fig. 1, *C* and *D*). The majority of normal samples demonstrated reduced levels of ALT and TEL with a scattered distribution, while cancerous cells exhibited elevated levels of ALT and TEL, displaying a clustered spatial distribution pattern (Fig. 1*C*). Thus, TEL and ALT pathways generally show increased activity in cancer tissues compared to normal tissues, with some cancer types demonstrating more pronounced differences. Additionally, the ALT pathway exhibits significant variability across cancer types compared to TEL TMM.

### TMM phenotyping

After evaluating the activity of the ALT and TEL pathways in various cancer types, we aggregated the results to conduct segmented regression analyses and identify thresholds for the classification of samples into different TMM phenotypes. Subsequently, the categorized samples based on their ALT and TEL pathway activities PSF values were plotted in a two-dimensional scatter plot, representing an ALT *versus* TEL phenotypic space. We divide the TMM space into three categories: low, middle, and high to stratify the activity levels of the two TMM pathways accordingly.

**Figure 2:**
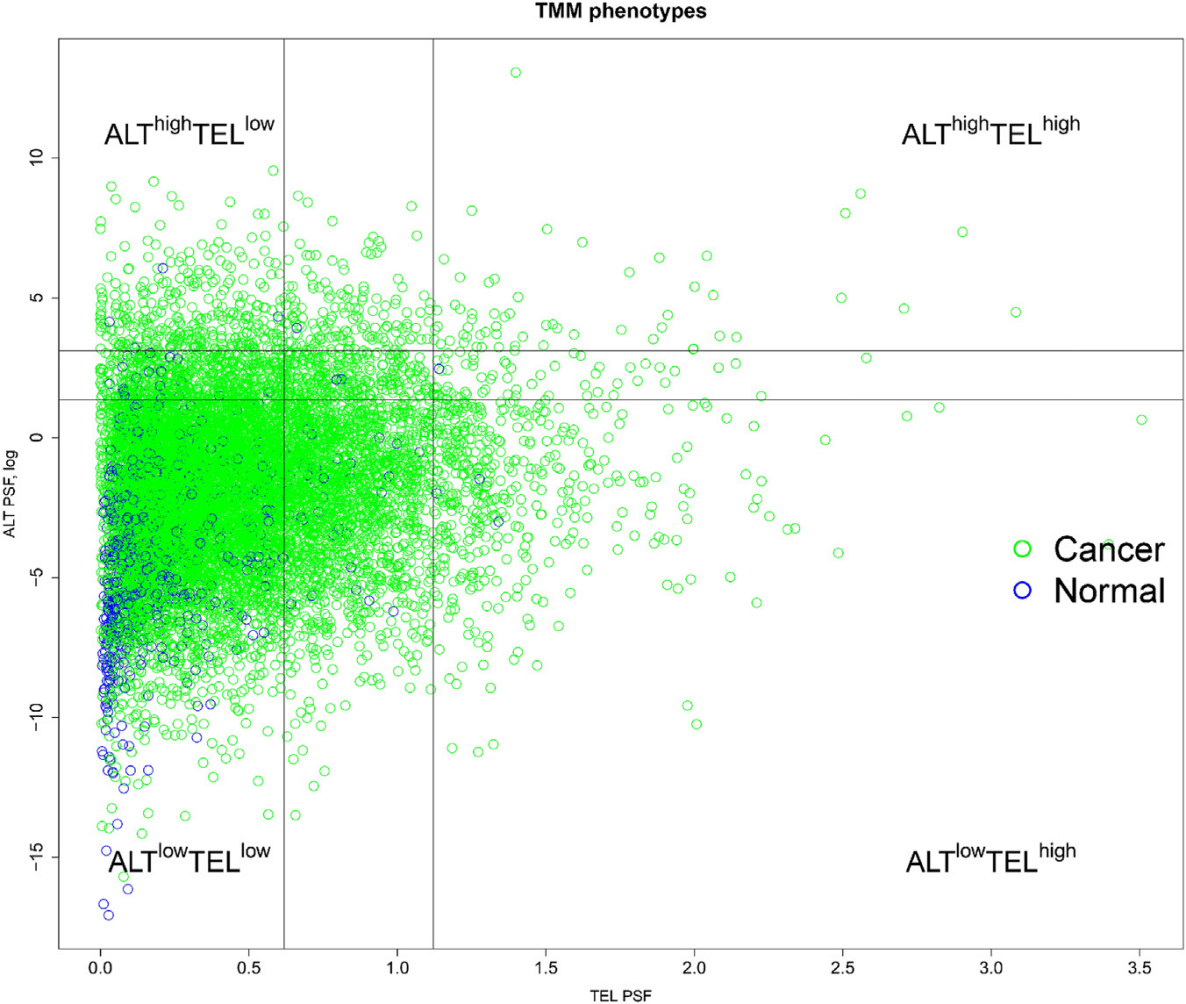
**Stratification of ALT and TEL TMM intofivephenotypes.** Cancer (*green*) and normal (*blue*) samples were divided into five groups based on their TEL PSF and ALT (log) PSF values, namely. ALT ^high^ TEL ^low^, ALT ^low^ TEL ^low^, ALT ^middle^ TEL ^middle^, ALT ^high^ TEL ^high^, and ALT ^low^ TEL ^high^ phenotypes. The *vertical* and *horizontal lines* show the thresholds used. ALT, alternative lengthening of telomeres; PSF, pathway signal flow; TEL, telomerase-dependent; TMM, telomere maintenance mechanism.

This division allowed us to classify the samples into five distinct phenotypes: ALT ^high^ TEL ^low^, ALT ^low^ TEL ^low^, ALT ^middle^ TEL ^middle^, ALT ^high^ TEL ^high^, and ALT ^low^ TEL ^high^. The phenotypes were determined based on the combinations of ALT and TEL pathway activities observed in the samples. Notably, the majority of matched normal samples were primarily located in the ALT ^low^ TEL ^low^ section of the TMM space, as represented in Figure 2. The analysis also reveals a notable prevalence of the ALT ^low^ TEL ^low^ phenotype in 64.49% of all cancers. Our data show that the "ALT ^high^ TEL ^high^ " and "ALT ^middle^ TEL ^middle^ " phenotypes were present in 31 to 40% of cancers. In particular, COAD and CHOL showed the highest percentages (40%) of these samples while lung adenocarcinoma and lymphoid neoplasm diffuse large B-cell lymphoma (DLBC) were the smallest (31%) of samples, with the main contribution coming from ALT ^middle^ TEL ^middle^ (Table S1). We also performed phenotyping for each individual cancer type separately using a similar TMM activity thresholding approach (Figs. S1–S3). Remarkably, the same distribution pattern was observed in almost all individually analyzed cancer types as well. In some cancer types, we observed only four phenotypes; ALT ^high^ TEL ^high^ was absent in including glioblastoma multiforme (GBM), adrenocortical carcinoma (ACC), CHOL, uterine carcinosarcoma (UCS), DLBC, liver hepatocellular carcinoma (LIHC), mesothelioma, ovarian serous cystadenocarcinoma (OV), sarcoma (SARC), and uveal melanoma (UVM) (Table S1 and Figs. S1, *C*–*E*, S2*D*, S3, *B* and *D*, *E*, *F*, *H* and *I*).

Overall, the identified phenotypes based on ALT and TEL pathway activities are consistent across multiple cancer types, suggesting shared patterns in the behavior of cancers.

### GBM single-cell TMM phenotyping

**Figure 3:**
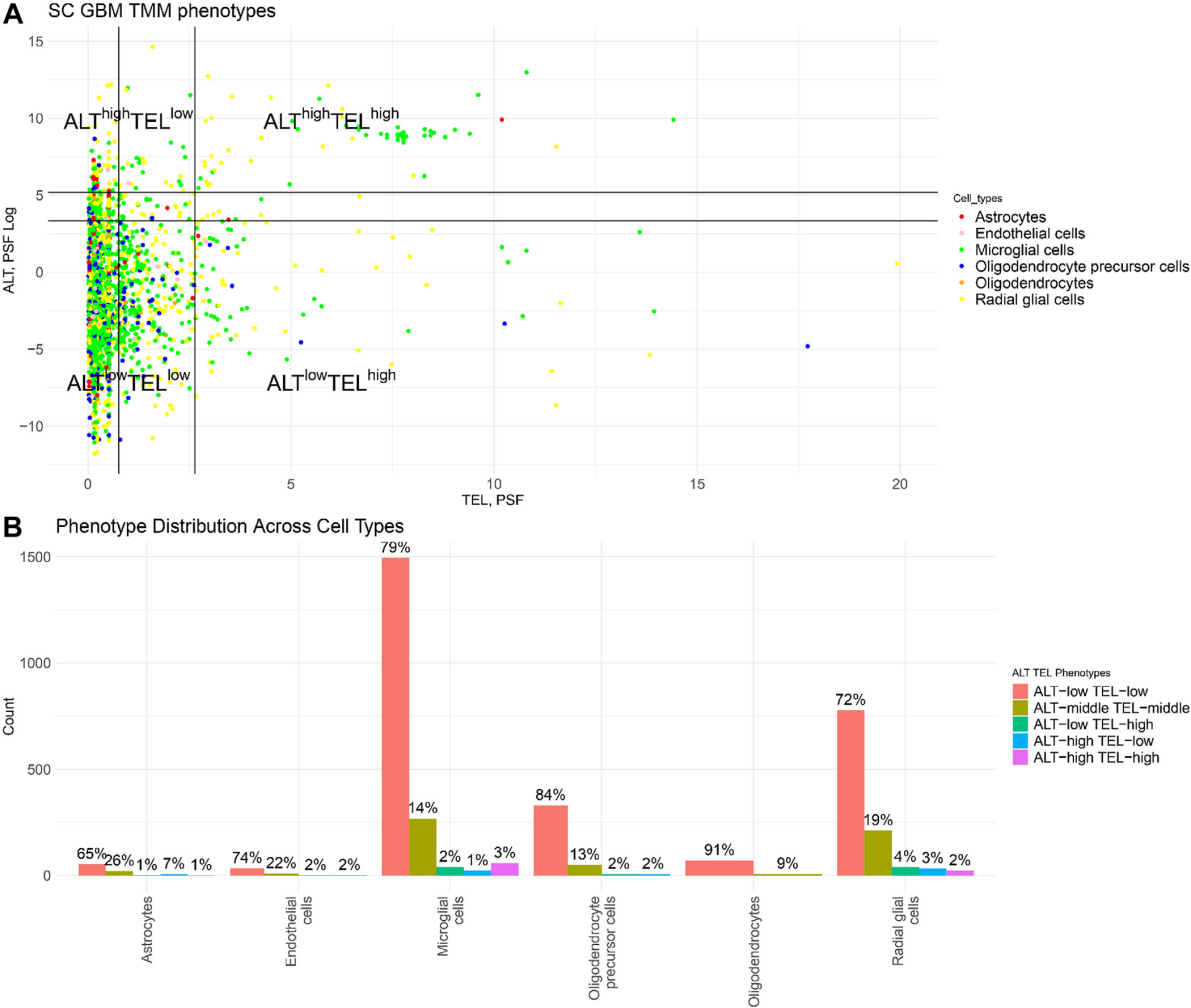
**Stratification of ALT and TEL TMM phenotypes for single-cell GBM and phenotype activity plots for cell types.***A*, stratification of ALT and TEL TMM phenotypes. The color coding corresponds to the cell type. SC GBM is divided into five groups based on their TEL PSF and ALT (log) PSF values: ALT ^high^ TEL ^low^, ALT ^low^ TEL ^low^, ALT ^middle^ TEL ^middle^, ALT ^high^ TEL ^high^, and ALT ^low^ TEL ^high^ phenotypes. The vertical and horizontal lines show the thresholds used. *B*, phenotypes distribution across cell types by percentage. The color coding corresponds to the TMM phenotypes. ALT, alternative lengthening of telomeres; GBM, glioblastoma multiforme; PSF, pathway signal flow; SC, single cell; TEL, telomerase-dependent; TMM, telomere maintenance mechanism.

In order to understand the potential coexistence of ALT and TEL pathways within individual cells, we conducted single-cell (SC) analysis on GBM cancer cells using a similar methodology to that used for bulk analysis. Remarkably, our examination revealed a consistent distribution pattern across SC analyses, mirroring observations made at the bulk level. Notably, within this SC dataset, we identified a subset of cells exhibiting an ALT ^high^ and TEL ^high^ phenotype, suggesting the presence of both the ALT and TEL mechanisms within these cells (Fig. 3*A*). Moreover, 32% of all the cells were distributed in ALT ^high^ TEL ^high^, ALT ^low^ TEL ^high^, and ALT ^high^ TEL ^low^ phenotypes (Fig. 3*B*). These data suggest the TMM phenotype diversity across the cells obtained from the same cancer source as well as the coexistence of TMM-phenotypes in cancer cells.

### Tumor purity

**Figure 4:**
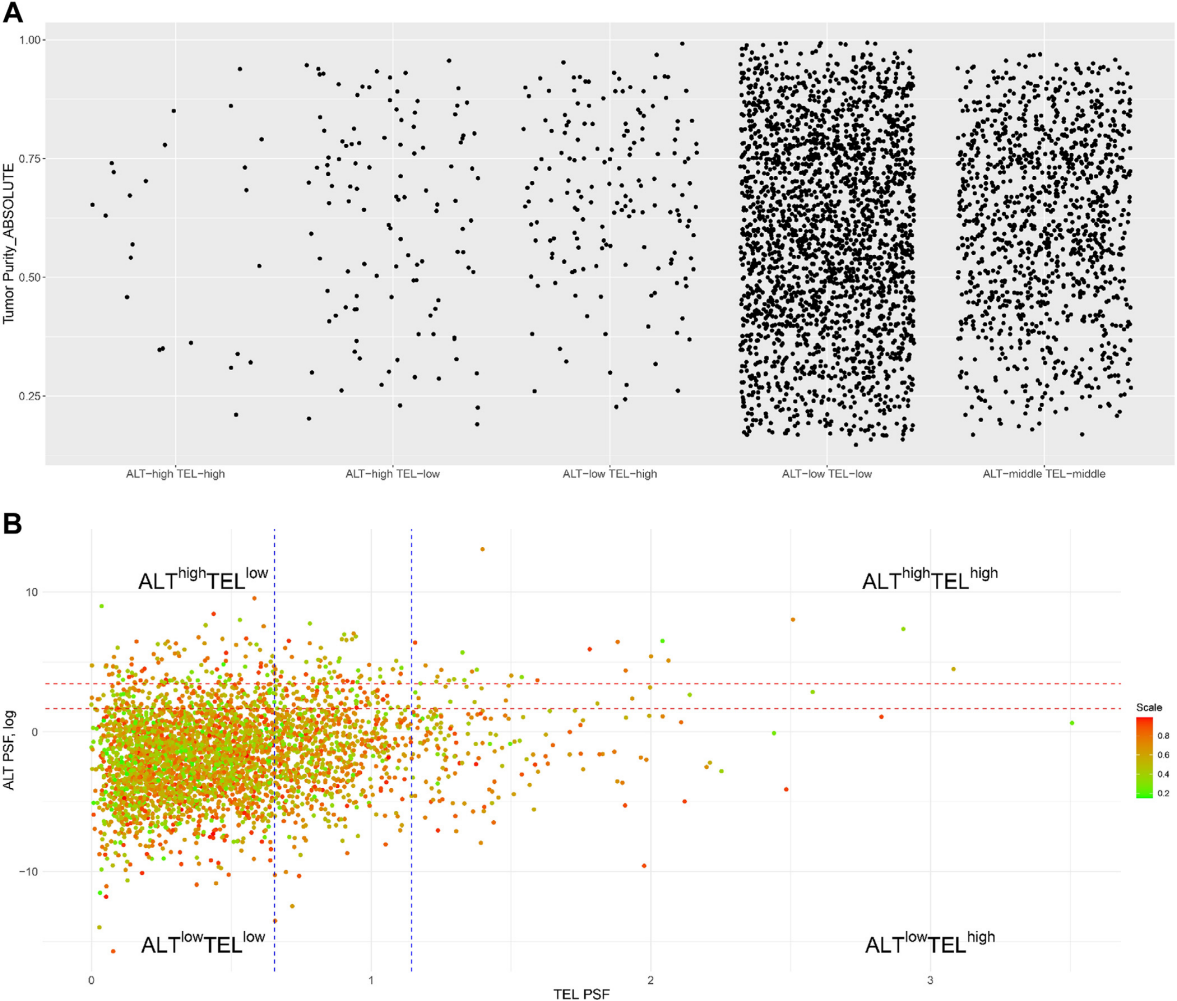
**ABSOLUTE estimation distribution of tumor purity for ALT and TEL phenotypes.***A*, illustrates the distribution of tumor purity across five phenotypic categories. *B*, TMM space is divided into four groups based on their TEL PSF and ALT (log) PSF values. The vertical and horizontal lines show the thresholds used. The *color coding* corresponds to the scale of tumor purity. ALT, alternative lengthening of telomeres; PSF, pathway signal flow; TEL, telomerase-dependent; TMM, telomere maintenance mechanism.

To gain insight into the factors influencing the observed distribution of samples across TMM phenotypes, we examined tumor purity estimation data obtained from five different methods as employed by Aran *et al.* (2015) (31). Our data reveal no definite pattern in tumor purity across different TMM phenotypes. Independent of tumor purity, we observed all TMM phenotypes (Fig. 4, *A* and *B*). However, the ALT ^high^ TEL ^high^ phenotype exhibited higher tumor purity consistently. A consistent pattern emerged across all five observed methods (Fig. S4, *A*–*H*). This suggests that tumor purity has little or no influence on derived TMM phenotypes.

### Microsatellite instability (MSI) and microsatellite stability status and TMM pathway branches

**Figure 5:**
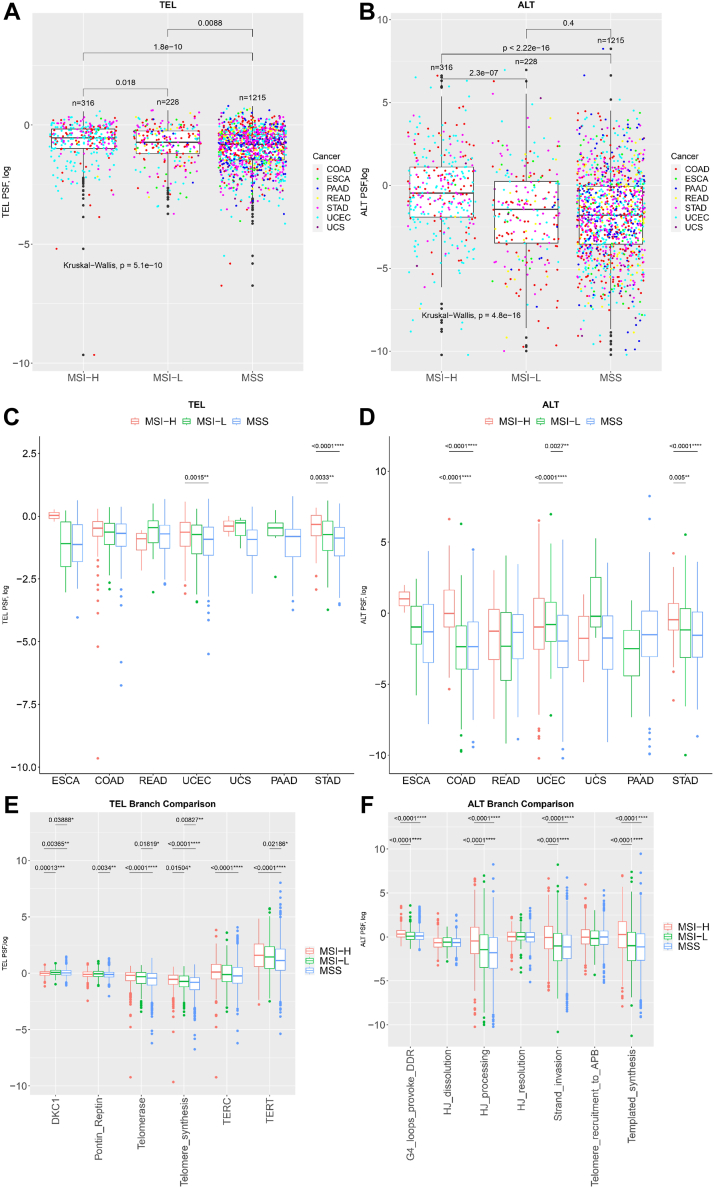
**PSF activity plots of TEL and ALT TMM pathways for MSI (microsatellite instable, high and low) and MSS (microsatellite stable) tumors.** Comparison of (*A*) TEL and (*B*) ALT TMM PSF activities for MSI and MSS tumors. Statistical analysis for MSS\MSI comparison used a Kruskal–Wallis test. *C*, TEL and (*D*) ALT pathway activity across seven cancer types. The *color coding* corresponds to the MSS\MSI status. Statistical analysis for MSS\MSI comparison used a Dunn’s test. PSF activities of different branches of the (*E*) TEL TMM and (*F*) ALT TMM pathways for MSS, MSI-L, and MSI-H tumors. ALT, alternative lengthening of telomeres; MSI, microsatellite instability; MSI-H, microsatellite instability high; MSI-L, microsatellite instability low; MSS, microsatellite stability; PSF, pathway signal flow; TEL, telomerase-dependent; TMM, telomere maintenance mechanism.

We obtained microsatellite instability low (MSI-L) and high (MSI-H) profiles, as well as microsatellite stability (MSS) profiles, for several cancer types including COAD, READ, esophageal carcinoma (ESCA), pancreatic adenocarcinoma, STAD, uterine corpus endometrial carcinoma (UCEC), and UCS from the TCGA dataset. These profiles were combined to create a unified dataset for analysis, and matched normal samples have been excluded. Comparing the TEL and ALT pathway PSF values, we observed that MSI-H samples across cancer types exhibited relatively higher activity in both pathways (ALT *p* = 5.1 × 10^−10^, Kruskal–Wallis test and TEL *p* = 4.8 × 10^−16^, Kruskal–Wallis test) (Fig. 5, *A* and *B*). Next, we applied a similar analysis separately for each of the mentioned cancer types. Specifically, MSI-H-related high activity was seen in the ALT and TEL pathways of UCEC (TEL *p* = 0.0015, Dunn’s test and ALT *p* < 0.0001, Dunn’s test) and STAD (TEL *p* = 0.0033, Dunn’s test and ALT *p* = 0.005, Dunn’s test), as well as in the ALT pathway of COAD (ALT *p* < 0.0001, Dunn’s test) (Fig. 5, *C* and *D*). Overall, in the ALT pathway, the individual-level comparison revealed that most MSS cancer samples have lower ALT pathway activity compared with MSI-H (Fig. 5*D*).

To assess the implications of ALT and TEL pathway branch activity concerning the MSS, MSI-L, and MSI-H status, we analyzed TEL and ALT pathway branch-level activity across seven cancer types. The comparative analysis of PSF scores of the major TMM-pathway branches revealed that TEL pathway activation in MSI-H occurs mainly through TERT and TERC branches (Fig. 5*E*). We observed a more diverse pattern in the ALT pathway, several branches were activated namely Holliday junction processing, Telomere recruitment to APB, strand invasion, and templated synthesis branches. Moreover, for the MSI-H subtype, the Holliday junction processing, strand invasion, and templated synthesis branches exhibit significantly higher (*p* < 0.0001, Dunn’s test) activation levels compared to the MSS subtype (Fig. 5*F*). We also performed individual-level comparisons for the mentioned cancer types separately. Through this individual-level comparison analysis, we observed similar patterns for the TEL and ALT pathways branches PSF levels among several cancer types (Fig. S5, *A* and *B*). In summary, MSI-H samples generally exhibit higher activity in the ALT and TEL pathways, with specific branches contributing to this pathway activation, namely TERT and TERC branches for TEL and Holliday junction processing, telomere recruitment to APB, strand invasion, and templated synthesis branches for ALT. This association was consistent across various cancer types, and the ALT pathway showed greater branch activation diversity than the TEL pathway.

### TMM phenotypes and survival probability analysis

**Figure 6:**
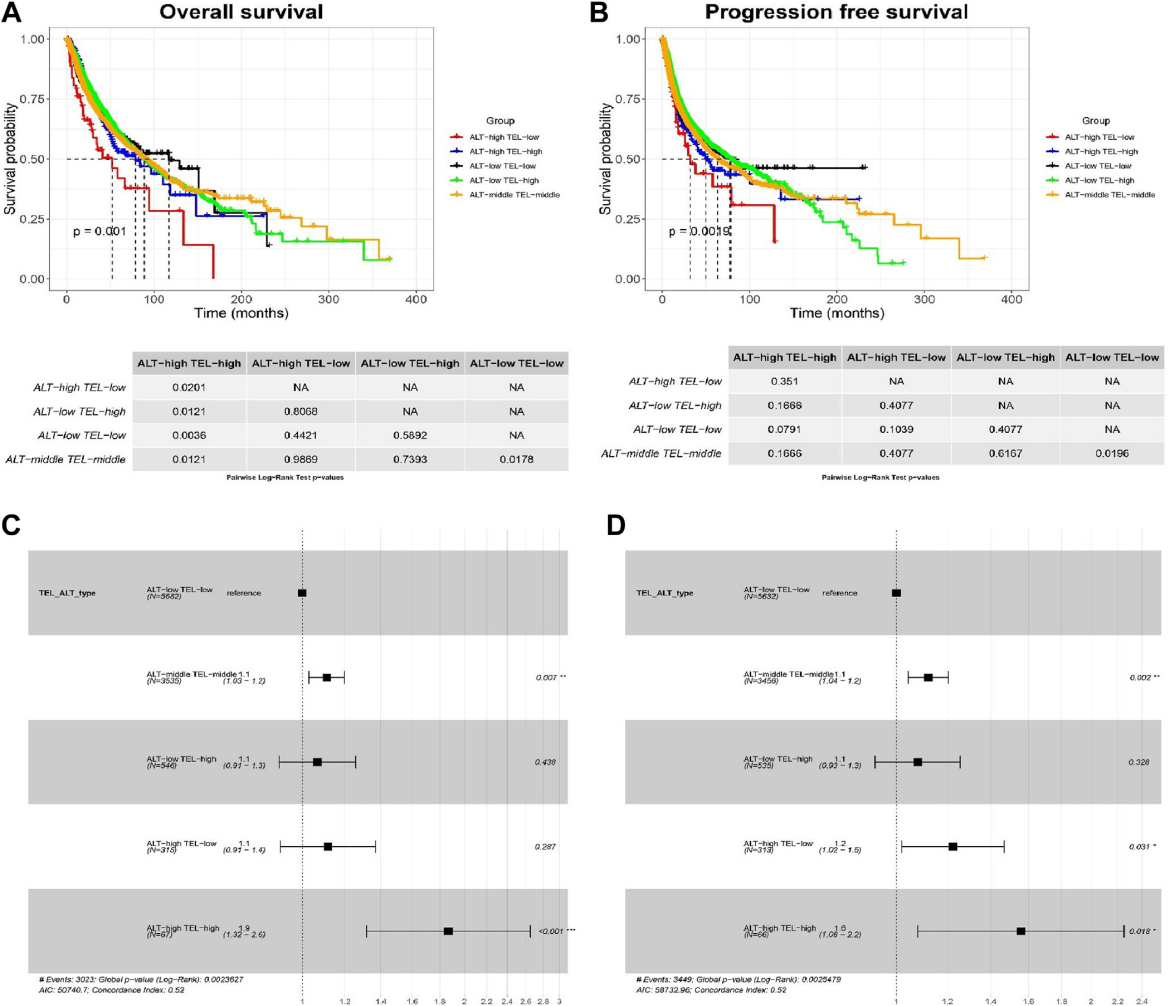
**Survival outcomes and Hazard ratios for ALT and TEL tumors.** Survival and hazard ratio forest plots for ALT and TEL tumors (*A*) and (*B*) overall survival. *C* and *D*, progression-free survival curves revealed a furious prognosis for ALT ^high^ TEL ^low^ tumors. Significance was calculated using a log-rank test for K-M plots, and a Cox proportional hazards regression model was used to estimate hazard ratios. Adjacent normal samples have been removed. The pairwise log-rank test was used to assess the significance between TMM phenotype groups. ALT, alternative lengthening of telomeres; TEL, telomerase-dependent; TMM, telomere maintenance mechanism.

We performed survival analyses for TMM phenotypes based on the corresponding thresholds in a combined dataset containing all 33 cancer types (see TMM phenotyping section). Our analysis revealed significant differences in the overall and progression-free survival rates. Log-rank test has been used to calculate global *p* values (Fig. 6, *A* and *B*). The Cox regression model was used for hazard ratio estimation (Fig. 6, *C* and *D*). We observed significant differences between ALT ^high^ TEL ^high^ *versus* ALT ^low^ TEL ^low^ (*p* = 0.003), and ALT ^middle^ TEL ^middle^ *versus* ALT ^low^ TEL ^low^ (*p* = 0.01 and *p* = 0.01, respectively) for survival outcomes (Fig. 6, *A* and *C*). Survival curves of the progression-free survival for ALT ^high^ and TEL ^low^ tumors revealed an unfavorable prognosis. Furthermore, we observed varying survival outcomes for TMM phenotypes across different individual cancer types. Detailed results can be found in the Supporting Information (Figs. S6–S38). Notably, significant differences were observed at the individual level for the following cancers, in kidney chromophobe where both end points showed significant differences (shortening of survival) for ALT ^high^ TEL ^high^, ALT ^high^ TEL ^low^ *versus* ALT ^low^ TEL ^low^ (overall survival *p* < 0.001 and progression-free survival *p* < 0.001, respectively) (Fig. S16), bladder urothelial carcinoma significant differences (shortening of survival) were observed for the progression-free endpoint in ALT ^high^ TEL ^high^ *versus* ALT ^low^ TEL ^low^, ALT ^low^ TEL ^high^ and ALT ^middle^ TEL ^middle^ (*p* = 0.0018, *p* = 0.0018, and *p* = 0.04, respectively) (Fig. S7). Moreover, several cancer types showed significant differences for several phenotypes, namely kidney renal clear cell carcinoma (Fig. S17), brain lower grade glioma (Fig. S20), PRAD (Fig. S28), and SARC both end points showed significant differences (Fig. S30). For thymoma (THYM) cancers, significant differences were observed for the progression-free end point shortening of survival for ALT ^high^ TEL ^high^ and ALT ^high^ TEL ^low^ (Fig. S35).

### Analysis of gene expression and gene set enrichment across TMM phenotypes

**Figure 7:**
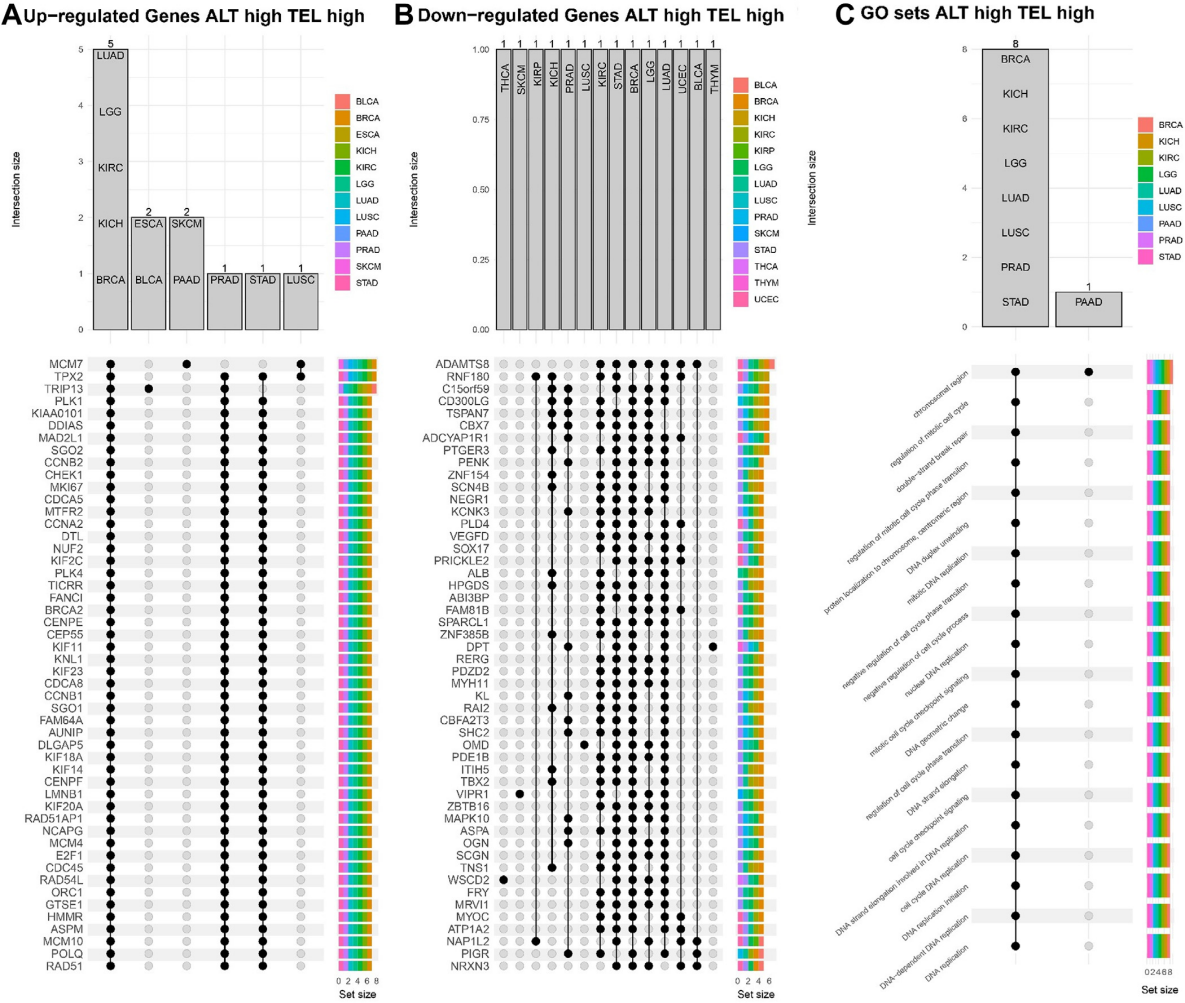
**Upregulated and downregulated genes and overrepresented GO terms are shown as UpSet plots for the pan-cancer data.***A*, top-50 list of frequently upregulated genes for ALT ^high^ TEL ^high^ tumors. *B*, top 50 list of frequently downregulated genes (*C*) overrepresented GO terms by cancer types for ALT high TEL high phenotype. The top-20 terms were chosen for overrepresentation analysis. ALT, alternative lengthening of telomeres; GO, gene ontology; TEL, telomerase-dependent.

Next, we performed a differential gene expression analysis for TMM phenotypes. For this analysis, we used ALT ^low^ and TEL^low^ as a reference phenotype. We focused on identifying upregulated and downregulated genes specific to each phenotype and targeted the 50 most frequently overexpressed and underexpressed genes across different cancer types for each phenotype (Figs. 7, *A* and *B*, and S39–S41, *A* and *B*). We found that in the case of the ALT ^high^ TEL ^high^ phenotype, genes *TRIP13*, *TPX2*, and *MCM7* were upregulated in eight cancer types and *ADAMTS8* in seven cancer types was downregulated (Fig. 7, *A* and *B*). For the ALT ^high^ and TEL^low^ phenotype, the *BRIP1* and *CLSPN* genes were upregulated in 12 different cancer types and the *MTND1P23* pseudogene was downregulated in 14 cancer types, and *SCGB3A1* in 12 cancer types (Fig. S39, *A* and *B*). In the case of the ALT ^low^ TEL ^high^ phenotype, we observed that the *AURKB* gene was upregulated in 11 cancer types (Fig. S40*A*). Additionally, the RNA gene Y_RNA was found to be upregulated in 10 cancer types and *C7* and *OGN* genes were downregulated in 15 cancers (Fig. S40, *A* and *B*). For the ALT ^middle^ TEL ^middle^ phenotype, we also identified upregulated and downregulated genes in different cancer types. These included *CALB1*, *AURKB*, *SEPT14*, and the histone-coding gene *HIST1H1B* and downregulated *ADH1B* and *C7* in 11 cancer types (Fig. S41, *A* and *B*). We found seven upregulated genes (*MCM10*, RAD51-associated protein 1 (*RAD51AP1*), *KIF2C, Y_RNA, FAM64A, CDC6, and CDCA8*) and six downregulated genes (*ADAMTS8, ATP1A2, MYH1, OGN, OMD*, and *ZBTB16*) in at least two phenotypes containing either ALT ^high/middle^ or TEL ^high/middle^ regardless the cancer type. The complete list of upregulated and downregulated genes for each phenotype for all cancer types can be found in the Supporting information (Tables S2–S9).

After conducting differential gene expression analysis, we performed gene ontology (GO) enrichment analysis. By mapping differentially expressed genes with known GO terms, we identified the functional categories overrepresented among the differentially expressed genes in TMM phenotypes by cancer types (Figs. 7*C*, and S39–S41*C*). The GO analysis revealed that cell cycle progression and regulation, chromosomal dynamics and segregation, and DNA replication-related GO terms were overrepresented in the ALT ^high^ TEL ^high^, ALT ^high^ TEL ^low^, and ALT ^middle^ TEL ^middle^ phenotypes (Figs. 7*C*, S39 and S41*C*). DNA biosynthetic process was overrepresented in the ALT ^low^ TEL ^high^ and ALT ^middle^ TEL ^middle^ phenotypes (Figs. S40 and S41*C*).

We also separately conducted differential gene expression analysis and GO enrichment analysis for the TMM phenotypes across each cancer type. The detailed results of these analyses can be found in the Supporting information (Figs. S42–S74).

**Figure 8:**
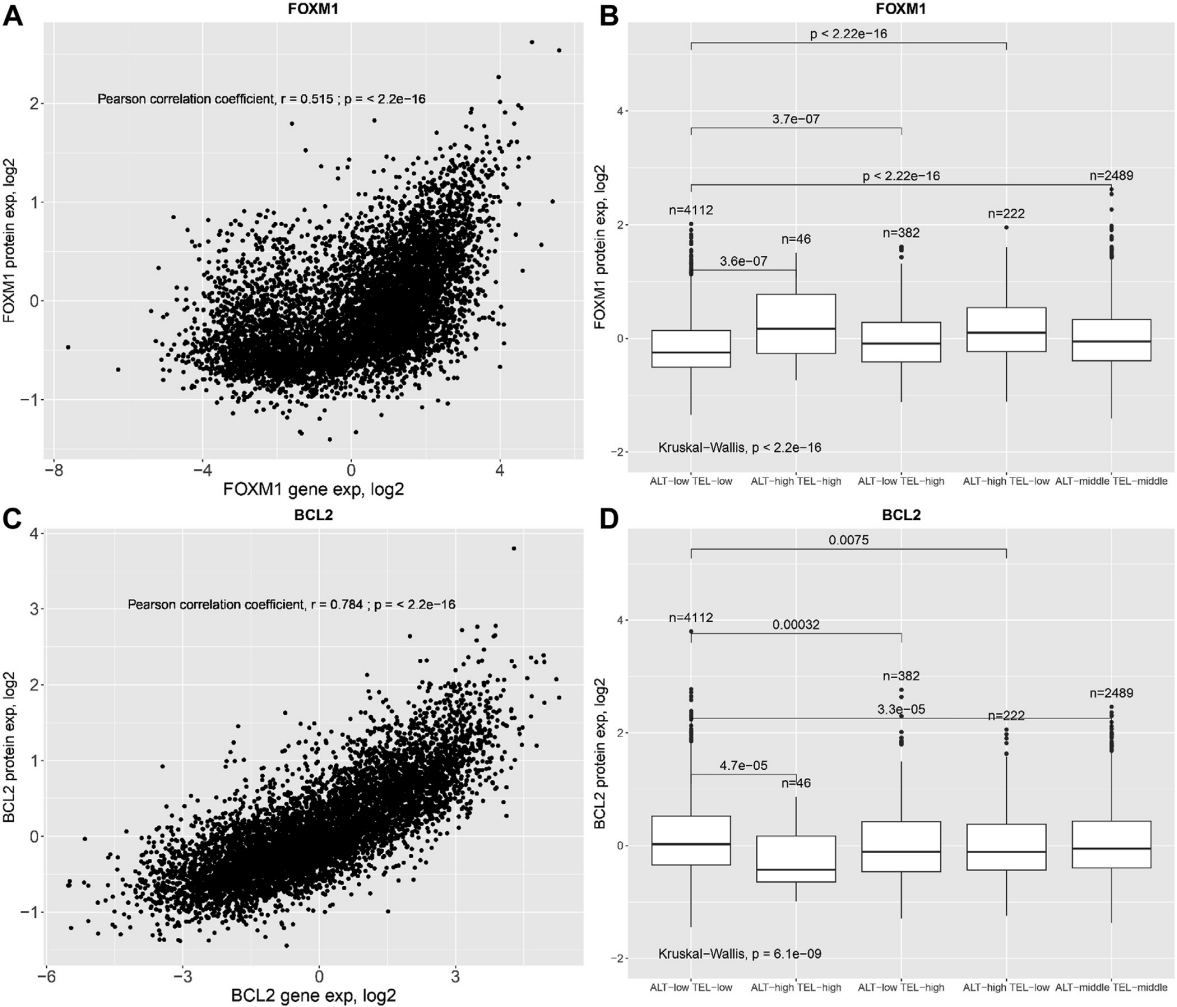
**Correlation plots of the relationship between protein expression *versus* RNA-seq gene expression, and proteome-level TMM phenotype activity plots for all cancer types.***A* and *B*, FOXM1 gene. Statistical analysis for TMM phenotypes comparison used a Kruskal–Wallis test. *C* and *D*, BCL2 gene. Statistical analysis for TMM phenotypes comparison used a Kruskal–Wallis test. TMM, telomere maintenance mechanism.

To evaluate the protein-level TMM patterns, we conducted a comparative analysis of protein-level expression patterns with both RNA-seq expression and TMM phenotypes. Data were obtained from The Cancer Proteome Atlas (32). Out of the 26,293 upregulated and 22,465 downregulated genes, data were available for only 153 genes in The Cancer Proteome Atlas. Results of our analysis of upregulated and downregulated genes, available in The Cancer Proteome Atlas, can be found in the Supporting information (Table S10). Our analysis revealed a robust linear correlation between protein expression levels and RNA gene expression for the BLC2 gene (Pearson correlation coefficient, r = 0.785; *p* < 2.2 × 10^−16^) (Fig. 8*B*), as well as a moderate linear correlation for the FOXM1 gene (Pearson correlation coefficient, r = 0.515; *p* < 2.2 × 10^−16^) (Fig. 8*A*) for all combined cancer types. Furthermore, a strong linear correlation was observed for the KIF2C gene (Pearson correlation coefficient, r = 0.712; *p* < 1.16 × 10^−17^) in the breast invasive carcinoma (BRCA) cancer type (Fig. S76*A*), and a moderate linear correlation for RAD51AP1 (Pearson correlation coefficient, r = 0.308; *p* = 0.0076) (Fig. S76*C*). In addition, the expression levels of the proteins were analyzed across various TMM phenotypes, for the FOXM1 all phenotypes showed significantly higher expression compared with ALT ^low^ TEL ^low^ phenotype (*p* < 2.2 × 10^−16^, Kruskal–Wallis) (Fig. 8*B*). Moreover, for KIF2C ALT ^low^ TEL ^high^ and ALT ^middle^ TEL ^middle^ compared with ALT ^low^ TEL ^low^ showed significantly higher expression (*p* = 0.019, Kruskal–Wallis) (Fig. S76*B*). Additional results can be found in the supporting information (Fig. S75, *A*–*H*).

## Discussion

### Diversity of TMM activation in different types of cancers

The activation of TMMs is a characteristic feature observed in the majority of cancer types (33, 34)․ However, our understanding of the association of TMM with the molecular mechanisms and clinical outcomes in cancers remains limited. The specific mechanisms that trigger the engagement of these pathways in telomere maintenance are not yet well-characterized. The results of our study provide insights into the activity of telomere maintenance mechanisms in various types of cancer. By analyzing RNA-seq data from tumor samples and matched normal samples obtained from TCGA, we focused on two pathways involved in telomere maintenance: the TEL pathway and the ALT pathway. It is essential to note that in our study the ALT pathway demonstrated greater variability across different cancer types. This finding indicates that certain cancers might be more predisposed or receptive to using the ALT pathway for potentially contributing to telomere maintenance and cell proliferation. This is in agreement with the current understanding of ALT telomere maintenance mechanisms (29, 35). Tumors originating from mesenchymal tissues show a higher incidence of ALT activation compared to once arising from epithelial tissues (16, 36). However, previously it has been reported that among 94 diverse cancer subtypes, the presence of the ALT phenotype in approximately 3.73% of all cases. Notably, the prevalence of the ALT phenotype exhibited significant variation across different cancer subtypes, highlighting its heterogeneous occurrence in various cancer types (37). Moreover, in multiple epithelial cancer types, the ALT phenotype was detected with varying prevalence rates, signifying its diverse occurrence among different types of epithelial cancers such as urinary bladder carcinomas (7%) (37), breast carcinomas (2%) (38), MSI-H gastric carcinomas (57%) (39), and hepatocellular carcinoma (7%) (37). The simultaneous high activity of ALT and TEL pathways in certain cancers provides evidence for the coexistence of these mechanisms in telomere maintenance. Several studies have reported instances where both ALT and TEL pathways are active within the same cancer type, suggesting a potential interplay or complementarity between these two mechanisms (40, 41, 42).

The ALT ^high^ TEL ^high^ and ALT ^middle^ TEL ^middle^ phenotypes are predominantly observed across various cancer types, with percentages ranging from approximately 31.2% to 40.4%, notably led by the prevalence of the ALT ^middle^ TEL^middle^ phenotype. Our data suggest the greater contribution of ALT-TMM in telomere maintenance in cancers than was thought before. The TMM pathway activities were generally higher in cancer cells than in matched normal cells across most cancer types. These findings support the elevated activity of telomere maintenance in cancer cells compared to most normal cells (43). As previously shown, the analysis of 264 normal tissues from different tissue types and 541 benign neoplasms originating from various tissue types did not reveal any evidence of the ALT phenotype in these noncancerous tissue samples (37).

It is pertinent to highlight that in our study within some cancer types, the adjacent normal samples displayed notable elevation in the activity of TMM. One explanation for this disparity could be tumor-specific aberrations in adjacent normal cells. Several studies have presented evidence of such a phenomenon in tumor-adjacent cells (44). The corresponding study of 13 head and neck squamous cell carcinoma showed that in the eight cases (61%) a genetically related precursor lesion was detected from the primary tumors field, suggesting that tumor-adjacent residual cancer cells were the origin of the recurrence (45). In addition, the gene expression analysis in prostate cancer revealed that gene expression profiles for each specimen class in tumor-adjacent normal cells were more similar to tumors than normal cells (46). Based on ALT and TEL pathway activity levels, we categorized the cancer samples into five TMM phenotypes (ALT ^high^ TEL ^low^, ALT ^low^ TEL ^low^, ALT ^middle^ TEL ^middle^, ALT ^high^ TEL ^high^, and ALT ^low^ TEL ^high^). It is important to note that certain cancer types, namely the ACC, CHOL, DLBC, GBM, LIHC, mesothelioma, OV, SARC, UCS, and UVM, did not exhibit the ALT ^high^ TEL ^high^ phenotype, indicating potential variations in telomere maintenance mechanisms specific to these cancers. Matched normal samples were primarily located in the ALT ^low^ TEL^low^ section of the TMM space, suggesting lower TMM activity in normal cells. Moreover, it is worth mentioning that the vast majority of cancer samples were predominantly found in the ALT ^low^ TEL ^low^ section. A possible explanation for this may be ALT cells that do not use APBs for telomere maintenance in the absence of telomerase-dependent TMM. A study on immortalized cell lines demonstrated that telomerase-negative cells utilize alternative ALT mechanisms, lack APB structures, and exhibit highly heterogeneous telomere length distribution typical of ALT cells. Our findings from our SC analysis of GBM cancer cells confirmed the observations from bulk RNA seq data. On one side, we found many glioblastoma cells exhibited TEL^low^ ALT ^low^ phenotype, on the other side provided insights into the potential coexistence of ALT and TEL pathways within individual cells. Of particular interest is the identification of a subset of cells displaying both an ALT ^high^ and TEL ^high^ phenotype. This finding strongly suggests the concurrent presence of both mechanisms within these cells, highlighting the complexity of telomere maintenance. Tumor heterogeneity poses a significant challenge in cancer genomics and epigenomics research, particularly when analyzing solid tumor tissues. These tissues are complex mixtures of cancer cells, adjacent normal tissues, stromal, and infiltrating immune cells. Our findings suggest that tumor purity may not exert an influence on the classification of cancer phenotypes.

Overall, this research provides insights into the complex telomere maintenance mechanisms and the adaptability of cells to cope with telomerase deficiency with alternative ALT-TMM (47).

### Enhanced TMM pathways activation in MSI-H *versus* MSS cancers

We also investigated the association of TMM phenotypes with clinical characteristics and outcomes. Our results indicated that samples with MSI-H status demonstrated comparatively higher activity in both telomere maintenance pathways. These results are in agreement with previous studies that have demonstrated the correlation between MSI and specific cancer types, especially COAD (48, 49, 50). In a comprehensive study of 24 different cancer types, there was a subgroup of 12 tumors exhibiting MSI which was analyzed. Remarkably, the findings indicated that a significant majority (83%) of MSI-associated cancers displayed elevated telomerase activity. This observation suggests that telomerase activation frequently occurs in tumors with MSI status (51). Moreover, a study in gastric carcinomas shows that the presence of hTERT with MSI-H was in 48% of cases and 86% detected in non-MSI-H cases. On the other hand, the number of ALT of APBs was higher in MSI-H (57%) compared to non-MSI-H cases (19%). The study also found that cases with hTERT-positive but APBs-negative were more common in non-MSI-H gastric carcinomas (76%) than in MSI-H gastric carcinomas (24%), while cases with hTERT-negative but APBs-positive were more common in MSI-H gastric carcinomas (33%) than in non-MSI-H cases (10%) suggesting that the mismatch repair system defects may contribute to ALT-TMM through homologous recombination of telomeric ends in gastric carcinomas.

The examination of PSF scores for the key branches within the TMM pathways unraveled that within the MSI-H subtype, the TEL pathway is predominantly activated through the TERT and TERC branches. ALT pathway revealed a more intricate and multifaceted pattern. Several branches of the ALT pathway exhibited activation, including, but not limited to, Holliday junction processing, telomere recruitment to APB, Strand invasion, and templated synthesis branches suggesting a more complex nature of the ALT pathway involvement. Moreover, our findings demonstrated that in the context of the MSI-H subtype, the Holliday junction processing, strand invasion, and templated synthesis branches exhibited significantly higher activation levels when compared to the MSS subtype.

Survival analyses for TMM phenotypes demonstrated significant differences in overall survival outcomes between ALT ^high^ TEL ^high^ and ALT ^low^ TEL ^low^, as well as ALT ^middle^ TEL ^middle^ and ALT ^low^ TEL ^low^ phenotypes. In particular, we observed a shortening of survival for ALT ^high^ TEL ^high^ and ALT ^high^ TEL ^low^ phenotypes. Our and other data indicate that TMM mechanisms potentially lead to delayed cellular senescence and increased proliferative capacity, resulting in more aggressive tumor behavior and may shorten patient survival. A recent study conducted by Roderwieser *et al.* (52) revealed that the activation of TEL and ALT characterize different neuroblastoma subgroups, and both mechanisms are associated with unfavorable outcomes. Pan-cancer analysis of TMM-dysregulation signature TMS score (TMScore) based on the molecular signature of TMMs, in 10,107 unique samples across seven cancer types revealed that TMScore was significantly associated with patient outcomes. Moreover, analysis of survival datasets from 5953 expression profiles showed that in breast and lung cancer, higher TMScores correlated with unfavorable outcomes (53).

### Exploring differentially expressed genes and enriched gene sets across diverse TMM phenotypes

Our study encompassed a comprehensive differential gene expression analysis among diverse TMM phenotypes, using ALT ^low^ TEL ^low^ as our baseline reference. Differential gene expressions were observed across various TMM phenotypes, and we identified that several genes were upregulated in a cancer-independent manner in phenotypes.

Our results indicate that the gene MCM10 was differentially expressed in all TMM phenotypes. In cancer cells, there is a consistent observation of upregulated expression of MCM10, as reported in studies by Cui *et al.* (2018) (54), and Wang *et al.* (2019) (55) suggesting that tumors rely on MCM10 to overcome genomic instability (56). Moreover, several studies indicate that a lack of MCM10 protein restricts the ability of telomerase to lengthen telomeres in a telomerase-dependent manner (57). The observations underscore the complex interplay between MCM10, telomerase, and telomere maintenance mechanisms in cancer cells. The upregulated expression of MCM10 in cancer cells may suggest its potential implication in TMM. However, further research is warranted to elucidate the precise molecular interactions between MCM10, telomerase, and other components involved in telomere maintenance pathways. The observed high expression of the gene in the ALT ^high^ TEL ^high^, ALT ^middle^ TEL ^middle^, and ALT ^high^ TEL ^low^ phenotypes suggests its potential role in telomere maintenance and tumor progression. A recent pan-cancer study analyzing the mRNA levels of KIF2C in various TCGA cancers revealed its widespread high expression across multiple cancer types, including BRCA, kidney renal papillary cell carcinoma, PRAD, OV, LIHC, UCEC, THYM, READ, UCS, DLBC, pheochromocytoma and paraganglioma, SKCM, brain lower grade glioma, CHOL, COAD, STAD, bladder urothelial carcinoma, head and neck squamous cell carcinoma, lung squamous cell carcinoma, lung adenocarcinoma, ESCA, kidney renal clear cell carcinoma, and ACC (58). KIF2C is a kinesin motor protein involved in microtubule dynamics and mitotic spindle formation, which are crucial for accurate chromosome segregation during cell division (59). In the context of TMM, the upregulation of KIF2C could be associated with its role in promoting cell proliferation and cell cycle progression, leading to enhanced tumor growth (60). Moreover, the observed high expression of KIF2C in ALT ^high^ TEL ^high^ and ALT ^high^ TEL ^low^ phenotypes may suggest its involvement in telomerase-independent telomere maintenance mechanisms. RAD51AP1 by interacting with the recombinase RAD51 facilitates homologous recombination and enhances its ability to form D-loops during the recombination process (61). In our study, we observed that RAD51AP1 was prominently expressed in three TMM phenotypes: ALT ^high^ TEL ^low^, ALT ^low^ TEL ^high^, and ALT ^high^ TEL ^high^. This finding aligns with a recent comprehensive pan-cancer study, which demonstrated upregulation of RAD51AP1 in 33 out of 34 analyzed cancer types (62). The elevated expression of RAD51AP1 in these TMM phenotypes suggests its potential significance in regulating homologous recombination mechanisms and may have implications for cancer development and progression.

Interestingly, we were able to confirm the results of differential expression using proteomic data. We observed a linear correlation between protein abundance and RNA gene expression levels for differentially expressed genes. Moreover, the protein levels were significantly different between TMM phenotypes. Unfortunately, the proteomic data were not available for many genes; however, our findings show a systematic pattern confirming our RNA-seq results.

We conducted GO analysis to gain a deeper understanding of how the TMM phenotypes connected with the diverse biological processes.

GO analysis results revealed that specific biological processes related to cell cycle regulation, and DNA replication are overrepresented in different telomere maintenance phenotypes. Namely, in ALT ^high^ TEL ^high^, ALT ^high^ TEL ^low^, and ALT ^middle^ TEL ^middle^ phenotypes, the enrichment of cell cycle checkpoint signaling terms, including "cell cycle checkpoint signaling," "regulation of cell cycle phase transition," and "mitotic cell cycle checkpoint signaling," suggests that cell cycle control mechanisms are actively engaged in the TMM regulations. Beyond its primary function in catalyzing the synthesis of telomeric DNA, telomerase plays multifaceted roles within cells. It is been demonstrated to significantly contribute to cell proliferation, ensuring genome stability, and acting as a protective mechanism against apoptosis or programmed cell death (63). Furthermore, the presence of terms related to chromosome dynamics and segregation, such as "chromosome segregation," "sister chromatid segregation," and "nuclear chromosome segregation," may imply that proper chromosome organization and segregation are essential for telomere maintenance in these phenotypes. Telomeres play a crucial role in stabilizing and protecting chromosome ends during replication and segregation, and any disruptions in these processes could lead to telomere dysfunction and genomic instability (64). Moreover, mutations in genes responsible for chromatin remodeling and H3.3 histones along with double-strand breaks induced telomere synthesis are highly associated with ALT-TMM (17). Moreover, the significant overrepresentation of DNA replication-related terms, such as "DNA replication," "DNA-dependent DNA replication," and "DNA replication initiation," suggests that the TMM is associated with active and robust DNA replication processes. This could indicate that cancer cells exhibiting this phenotype have an increased demand for DNA synthesis, which may be linked to their proliferative capacity and aggressive behavior. While our study provides insights into telomere maintenance mechanisms in various cancer types using bioinformatics analysis, it is important to acknowledge its limitations. Firstly, the study relies solely on bioinformatics data analysis rather than experimental validation. Although RNA-seq data from the tumor and matched normal samples were utilized, further experimental studies are needed to confirm the observed TMM activities. Moreover, the RNA level expression data may not translate to protein function and activity leading to misclassification of the TMM. Additionally, the number of samples analyzed varied depending on the specific type of cancer and the availability of data in the chosen dataset. This variability in sample size across different cancer types introduces potential biases and may impact the generalizability of the findings. In addition, another limitation arises from the lack of non-tumor tissue samples, as only matched adjacent normal samples were included in the analysis. This may introduce bias and potentially impact the interpretation of the data.

Moreover, the prevalence of cancer samples in the TEL ^low^ ALT ^low^ phenotype presents another limitation within our study, which may stem from the noncompleteness of the TMM pathways, or other mechanisms involved in TMM that were not considered.

Overall, our study sheds light on the differential activity of telomere maintenance mechanisms in various cancer types. The findings emphasize the complex nature of telomere regulation and highlight the potential significance of the TEL and ALT pathways in cancer development and progression. Further exploration of these mechanisms in specific cancer types may contribute to the identification of novel therapeutic targets and the development of personalized treatment strategies.

## Experimental procedures

### Data preprocessing

RNA sequencing raw count data for 11,123 samples from 33 cancer types have been obtained from TCGA (https://www.cancer.gov/tcga). Raw counts have been normalized for sequencing library size using the DESeq2 (version 1.34.0) (https://bioconductor.org/packages/DESeq2/) (65) R package and converted to log values. SVA R package (version 3.42.0) (https://bioconductor.org/packages/sva/) (66) has been used for the evaluation of batch effects and their correction. Log-transformed data have been mean-centralized over all samples and converted to FC values.

### Evaluation of telomere maintenance mechanism states across 33 cancer types

The state of TEL and ALT TMM pathways was evaluated using TMM signatures developed in our group (24, 27). Gene expression data and topologies of molecular interactions are used in the curated TMM pathways for TMM detection. Overall, TMM pathways contain 37 (ALT) and 26 (TEL) genes derived from 19 and 13 references (http://big.sci.am/software/tmm/tmm-genes/). The approach for comprehending the biology of telomere maintenance mechanisms that focuses on pathways takes into account the relationships between the genes involved and considers the impact of molecular events such as complex formation on TEL or ALT TMM. It is a method for evaluating the activity of the TEL and ALT telomere maintenance pathways in a sample using gene expression data and a PSF algorithm (25, 26). The PSF algorithm calculates the score for each node in the pathway by taking into account how signals propagate through interactions that activate or inhibit the pathway, as well as through the formation of complex and linker nodes. The calculation is based on the FC expression values of the genes in the pathway compared to their mean expression in the given data set. The PSF score provides a more complete assessment of the activity of a pathway by considering the expression levels of all genes involved, as well as the interactions between them. The PSF score offers a quantitative measurement of the activity of the TMM pathway in a given sample, giving a more comprehensive understanding of pathway activation. Thus, the PSF scores of the end point nodes in the pathway estimate the overall activity of TEL and ALT.

### TMM phenotyping

In order to assign TMM phenotypes to the samples, the segmented R package (version 1.6.0) (https://cran.r-project.org/web/packages/segmented/index.html) was used (67). We used two thresholds to divide TMM space into “low”, “middle”, and “high” phenotypes.

### TMM phenotyping of SC GBM

SC RNAseq data for glioblastoma were obtained from the Gene Expression Omnibus database, under accession number GSE84465. This dataset comprises 3589 cells isolated from both the tumor core and the surrounding peripheral tissue across four individual patients. The scRNA-seq analysis was performed using the Seurat R package (version 5.0.1) (68) and normalization of the data was carried out utilizing the SCTransform function within Seurat. For TMM activity calculations, we performed similar to bulk RNA-seq analysis. Furthermore, the segmented R package (version 1.6.0) was used for TMM space stratification.

### Tumor purity

Tumor purity data for TCGA cancer samples were obtained from the study by Aran *et al.*, 2015 (31). In their study, purity estimates for each sample were determined using four distinct methods: ESTIMATE, ABSOLUTE, LUMP, and IHC, and one combined method (31). The ESTIMATE purity estimation method used gene expression profiles of 141 immune genes and 141 stromal genes. ABSOLUTE method used somatic copy-number data. LUMP, on the other hand, is based on averaged nonmethylated immune-specific CpG sites. Lastly, purity estimates from IHC were derived from hematoxylin and eosin stain slide image analysis. In their study, they used the combined purity estimate method representing the median purity derived from each method after normalization with at least two measurements.

### MSI and MSS status and TMM pathway branches

The R package “TCGAbiolinks” (version 2.23.5) (69) was used to download the MSI and MSS status of the TCGA COAD, READ, ESCA, pancreatic adenocarcinoma, STAD, UCEC, and UCS datasets. For other cancer types, this information was not available at the time of our study.

The significance of the difference in ALT- and TEL-TMM activity values between MSI and MSS tumors was assessed using the Kruskall–Willis and Dunn’s test. *p* values < 0.05 were considered significant. Additionally, we performed branch-level activity difference assessment for these cancers. The TEL and ALT pathway branch-level information was extracted after the calculation of overall TMM activity based on the TMM pathways’ topologies (24, 27).

### TMM phenotypes and survival probability analysis

Kaplan–Meier survival curves have been used to estimate the overall survival, and progression-free survival depending on the defined TMM phenotypes using the survival (version 3.3.1) (https://cran.r-project.org/web/packages/survival/index.html) and survminer (version 0.4.9) (https://cran.r-project.org/web/packages/survminer/index.html) R packages. The pairwise log-rank test was used to assess the significance between TMM phenotype groups. The Cox regression model has been used for hazard ratio estimation (70).

### Analysis of gene expression and gene set enrichment across TMM phenotypes

We performed differential expression analysis for the ALT ^low^ TEL ^low^ phenotype compared to the ALT ^high^ TEL ^high^, ALT ^low^ TEL ^high^, ALT ^middle^ TEL ^middle^, ALT ^high^ TEL ^low^ in 33 cancer types, using the DESeq2 (version 1.34.0) (65) R package.

#### GO analysis

The lists of differentially expressed genes (*p*-adjusted < 0.05) for all TMM phenotypes for corresponding cancer types were subjected to functional annotation using gene set analysis (EnrichGO function) implemented in the clusterProfiler R package (version 4.2.2) (https://bioconductor.org/packages/clusterProfiler/) (71). The analysis was performed against gene sets from the biological process, cellular component, and molecular function GO categories. The *p*-values were adjusted for multiple testing using the Benjamini–Hochberg method, and a q-value cutoff of 0.05 was applied to identify statistically significant GO terms. For the visualization of the functional enrichment results: enrichplot R (version 1.21.0) (https://bioconductor.org/packages/enrichplot/) (72) package was used.

#### Proteomic data analysis

Reverse-phase protein array data were obtained from The Cancer Proteome Atlas (32). We used level 4 replicate-based combined reverse-phase protein array Pan-Cancer data (Pan-Can 32 L4) for proteome data analysis. Proteomic data for BRCA cancer type were obtained from Proteomic Data Commons (https://pdc.cancer.gov) (73) Study Identifier: PDC000173. Pearson’s correlation coefficient was used to assess the correlation between proteome *versus* normalized RNA-seq. Subsequently, FCs in gene expressions were calculated, and a log2 transformation was applied to the FC values before correlation analysis. For many genes, proteomic data were not available at the time of our study.

## Data availability

The TCGA (The Cancer Genome Atlas) data, including the RNA-seq data mentioned in the study, are publicly accessible through the NCI Genomic Data Commons (GDC) (https://gdc.cancer.gov/). All data are available in the main text or in the Supporting Information section.

## Supporting information

This article contains supporting information.

## Conflict of interest

The authors declare that they have no conflicts of interest with the contents of this article.
