# Supplementary material for: Pan-cancer analysis of telomere maintenance mechanisms
Source: J Biol Chem. 2024 May 18;300(6):107392. doi: 10.1016/j.jbc.2024.107392 (PMC11225560; doi:10.1016/j.jbc.2024.107392)
Supplement: Supporting Information [file mmc1.pdf]

Pan-cancer analysis of telomere maintenance mechanisms

Meline Hakobyan[1]*, Hans Binder[2,3] , Arsen Arakelyan[1]
1 - Bioinformatics Group, Institute of Molecular Biology NAS RA, Yerevan, Armenia
2 - Interdisciplinary Centre for Bioinformatics, University of Leipzig, Leipzig, Germany
3 - Armenian Bioinformatics Institute, Yerevan, Armenia
* Correspondence:
Corresponding author: anilem.hakobyan@gmail.com

Items included:

Table S1. Distribution of the samples by TMM phenotypes for cancer types.

Figure S1-S3. TMM Phenotyping for all cancer types separately.

Figure S4. Distribution of ALT and TEL pathway tumor purity for 5 phenotypes and tumor purity scale-based TMM phenotyping for 4 methods.

Figure S5 Activity plots of MSS/MSI statuses for TMM pathway branches for ALT and TEL pathways.

Figure S6-38. Survival and hazard ratio forest plots across different individual cancer types ALT and TEL phenotypes separately.

Figure S39-41. Up-regulated and down-regulated genes and over-represented GO terms UpSet plot for the pan-cancer studies for ALT ^{high} TEL ^{low}, ALT ^{low} TEL ^{high}, ALT ^{middle} TEL ^{middle} phenotypes.

Figure S42-74. Differential gene expression analysis and gene ontology enrichment analysis for the TMM phenotypes across different cancer types, conducted individually.

Figure S75. Correlation plots of the relationship between protein expression versus RNA-seq gene expression, and Proteom level TMM phenotype activity plots for all cancer types.

Figure S76. Correlation plots of the relationship between protein expression versus RNA-seq gene expression, and Proteom level TMM phenotype activity plots for BRCA.

Items included as separate Excel sheets:

Table S2: Pan-cancer down-regulated gene list for ALT ^{high} TEL ^{high} phenotype.

Table S3: Pan-cancer up-regulated gene list for ALT ^{high} TEL ^{high} phenotype.

Table S4: Pan-cancer down-regulated gene list for ALT ^{high} TEL ^{low} phenotype.

Table S5: Pan-cancer up-regulated gene list for ALT ^{high} TEL ^{low} phenotype.

Table S6: Pan-cancer down-regulated gene list for ALT ^{low} TEL ^{high} phenotype.

Table S7: Pan-cancer up-regulated gene list for ALT ^{low} TEL ^{high} phenotype.

Table S8: Pan-cancer down-regulated gene list for ALT ^{middle} TEL ^{middle} phenotype.

Table S9: Pan-cancer up-regulated gene list for ALT ^{middle} TEL ^{middle} phenotype.

Table S10: Protein-level TMM phenotypes comparison.

Table S1. **Distribution of the samples by TMM phenotypes for cancer types.**

Cancer type	TMM phenotypes									
	ALT-high TEL-high	ALT-high TEL-high %	ALT-high TEL-low	ALT-high TEL-low %	ALT-low TEL-high	ALT-low TEL-high %	ALT-low TEL-low	ALT-low TEL-low %	ALT-middle TEL-middle	ALT-middle TEL-middle %
ACC	-	-	5	6.33	2	2.54	54	68.36	18	22.79
BLCA	4	0.98	16	3.89	18	4.37	251	60.93	123	29.86
BLCA Normal	-	-	-	-	-	-	18	94.74	1	5.27
BRCA	9	0.8	53	4.71	59	5.24	667	59.19	339	30.08
BRCA Normal	-	-	-	-	-	-	98	98.99	1	1.02
CESC	2	0.66	12	3.93	9	2.95	208	67.98	75	24.51
CESC Normal	-	-	-	-	-	-	3	100	-	-
CHOL	-	-	-	-	3	8.58	18	51.43	14	40
CHOL Normal	-	-	-	-	-	-	9	100	-	-
COAD	-	-	7	1.46	23	4.8	256	53.34	194	40.42
COAD Normal	-	-	-	-	-	-	40	97.57	1	2.44
DLBC	-	-	2	4.17	2	4.17	29	60.42	15	31.25
ESCA	2	1.23	4	2.46	7	4.3	99	60.74	51	31.29
ESCA Normal	-	-	-	-	-	-	9	81.82	2	18.19
GBM	-	-	2	1.19	2	1.19	108	63.91	57	33.73
GBM Normal	-	-	-	-	-	-	5	100	-	-
HNSC	3	0.6	33	6.55	23	4.57	310	61.51	135	26.79
HNSC Normal	-	-	-	-	-	-	42	95.46	2	4.55
KICH	1	1.54	2	3.08	-	-	42	64.62	20	30.77

KICH Normal	-	-	-	-	-	-	19	76	6	24
KIRC	3	0.56	11	2.04	18	4.17	368	70.84	141	22.23
KIRC Normal	-	-	2	2.78	3	3.33	51	68.03	16	26.07
KIRP	-	-	4	1.38	5	1.72	198	68.05	84	28.87
KIRP Normal	-	-	-	-	-	-	31	96.88	1	3.13
LAML	-	-	5	3.34	10	6.67	82	54.67	53	35.34
LGG	5	0.94	16	3.01	10	1.88	377	70.87	124	23.31
LIHC	4	1.08	21	5.64	16	4.29	219	58.45	114	30.57
LIHC Normal	-	-	-	-	-	-	50	100	-	-
LUAD	11	2.04	19	3.52	28	5.19	323	59.82	159	29.45
LUAD Normal	-	-	-	-	-	-	57	98.28	1	1.73
LUSC	5	1	8	1.6	25	4.99	298	59.37	166	33.07
LUSC Normal	-	-	-	-	-	-	49	100	-	-
MESO	-	-	4	4.6	6	6.9	59	67.82	18	20.69
OV	1	0.27	14	3.68	20	5.25	279	73.23	67	17.59
PAAD	2	1.12	9	5.03	9	5.03	120	67.04	39	21.79
PAAD Normal	-	-	-	-	-	-	4	100	-	-
PCPG	1	0.55	10	5.44	8	4.35	122	66.31	43	23.37
PCPG Normal	-	-	-	-	-	-	2	66.67	1	33.34
PRAD	1	0.2	17	3.93	27	5.38	298	59.37	159	31.68
PRAD Normal	-	-	2	3.39	-	-	34	66.67	15	29.42
READ	-	-	1	0.6	4	2.4	103	61.68	59	35.33
READ Normal	-	-	-	-	-	-	10	100	-	-
SARC	-	-	14	5.33	9	3.43	147	55.9	93	35.37
SARC Normal	-	-	-	-	-	-	1	50	1	50

SKCM	2	0.43	9	1.91	17	3.61	316	66.95	128	27.12
SKCM Normal	-	-	-	-	-	-	1	100	-	-
STAD	2	0.54	6	1.6	18	4.8	224	59.74	125	33.34
STAD Normal	-	-	-	-	-	-	32	100	-	-
TGCT	-	-	2	1.29	11	7.06	92	58.98	51	32.7
THCA	-	-	9	1.76	13	2.53	351	68.29	141	27.44
THCA Normal	-	-	-	-	-	-	48	84.22	9	15.79
THYM	-	-	-	-	8	6.67	80	66.67	32	26.67
THYM Normal	-	-	-	-	-	-	1	50	1	50
UCEC	2	0.37	19	3.43	16	2.89	330	59.57	187	33.76
UCEC Normal	-	-	-	-	-	-	35	100	-	-
UCS	-	-	2	3.51	3	5.27	41	71.93	11	19.3
UVM	-	-	2	2.5	-	-	56	70	22	27.5

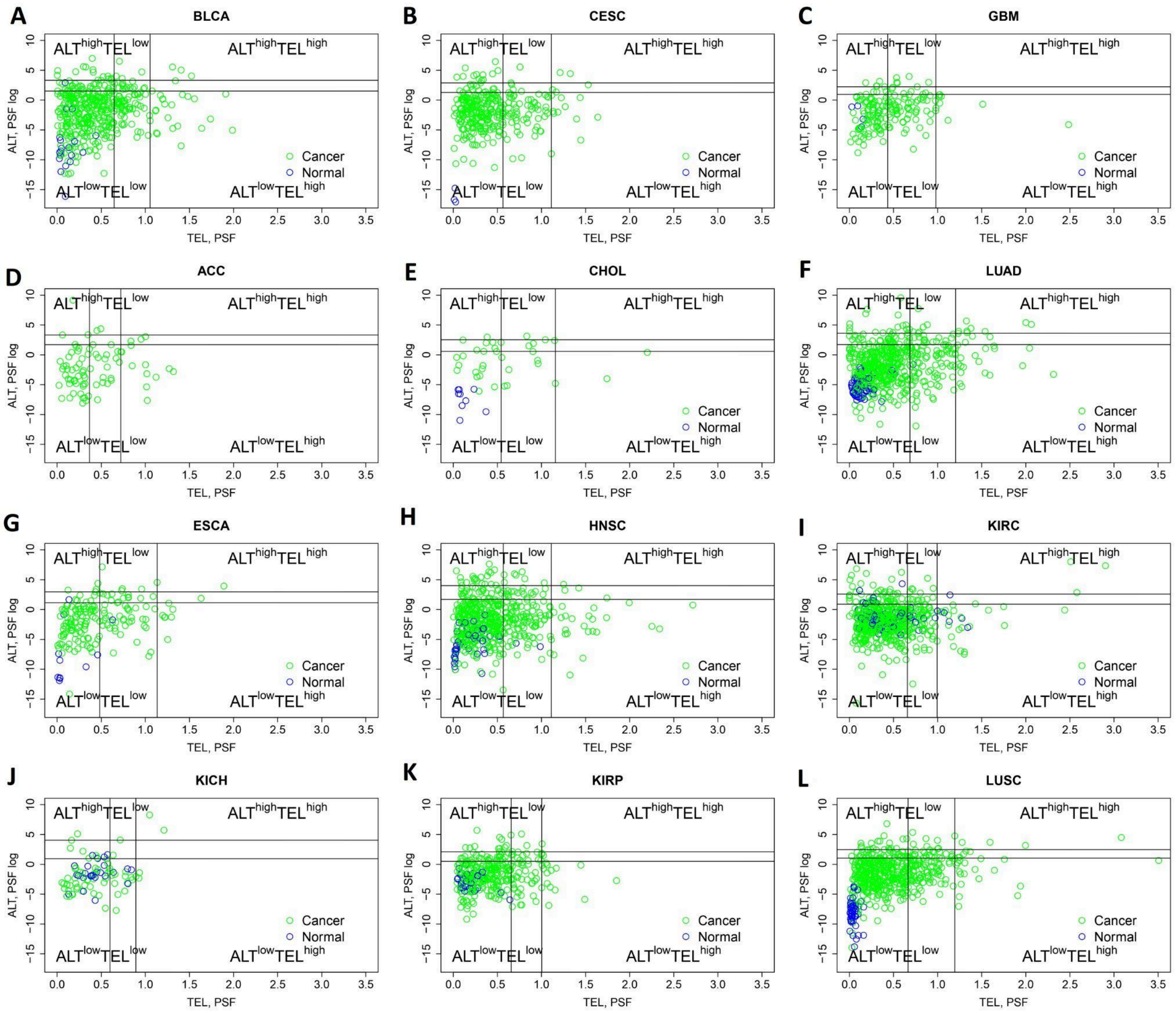

Figure S1 ALT and TEL pathways (**A**) BLCA, (**B**) CESC, (**C**) GBM, (**D**) ACC, (**E**) CHOL, (**F**) LUAD, (**G**) ESCA, (**H**) HNSC, (**I**) KIRC, (**J**) KICH, (**K**) KIRP and (**L**) LUSC TMM phenotyping. Cancer (green) and normal (blue) samples were plotted based on the TEL PSF and ALT (Log) PSF values. The vertical and horizontal lines reflect the threshold values.

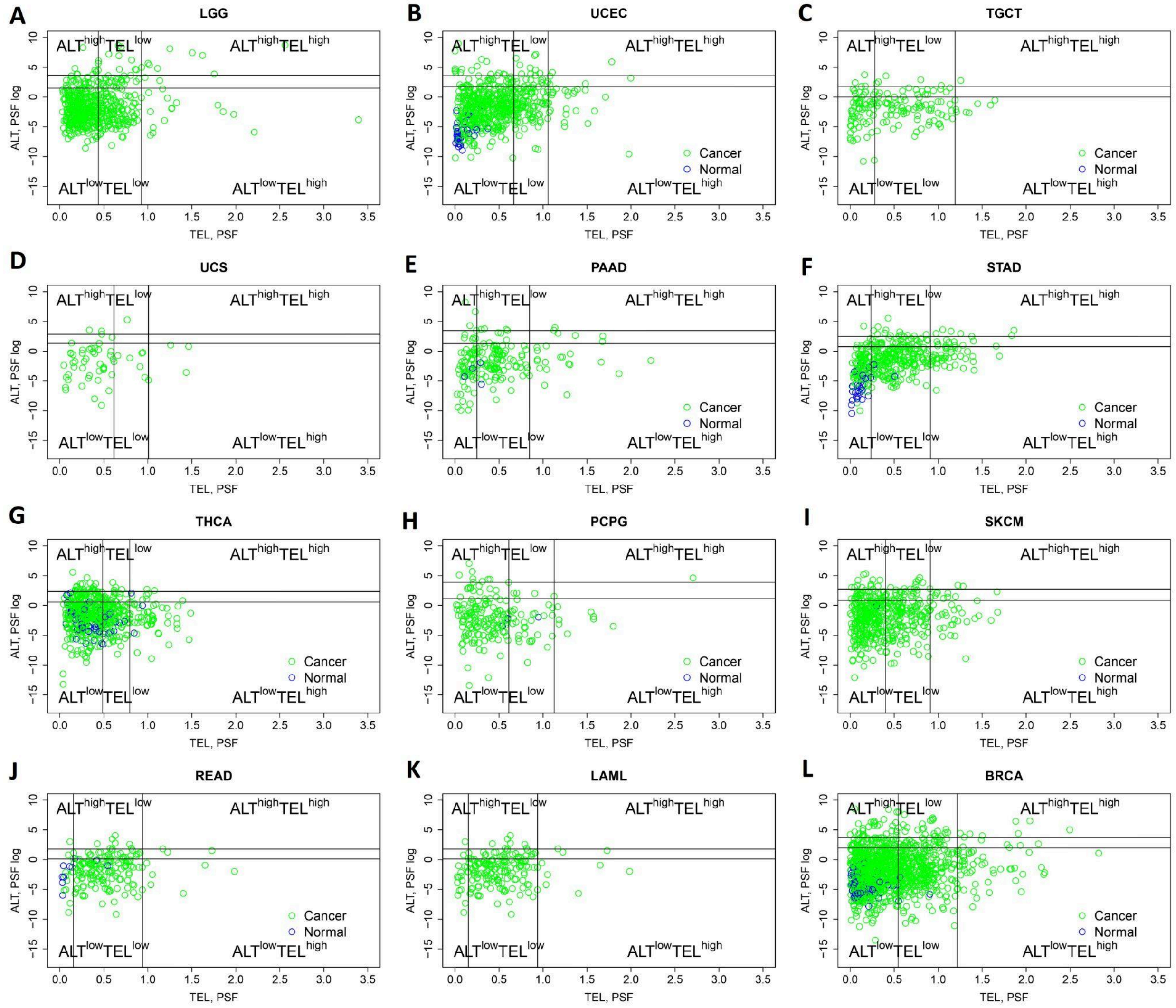

Figure S2. ALT and TEL pathways (**A**) LGG, (**B**) UCEC, (**C**) TGCT, (**D**) UCS, (**E**) PAAD, (**F**) STAD, (**G**) THCA, (**H**) PCPG, (**I**) SKCM, (**J**) READ, (**K**) LAML and (**L**) BRCA TMM phenotyping. Cancer (green) and normal (blue) samples were plotted based on the TEL PSF and ALT (Log) PSF values. The vertical and horizontal lines reflect the threshold values.

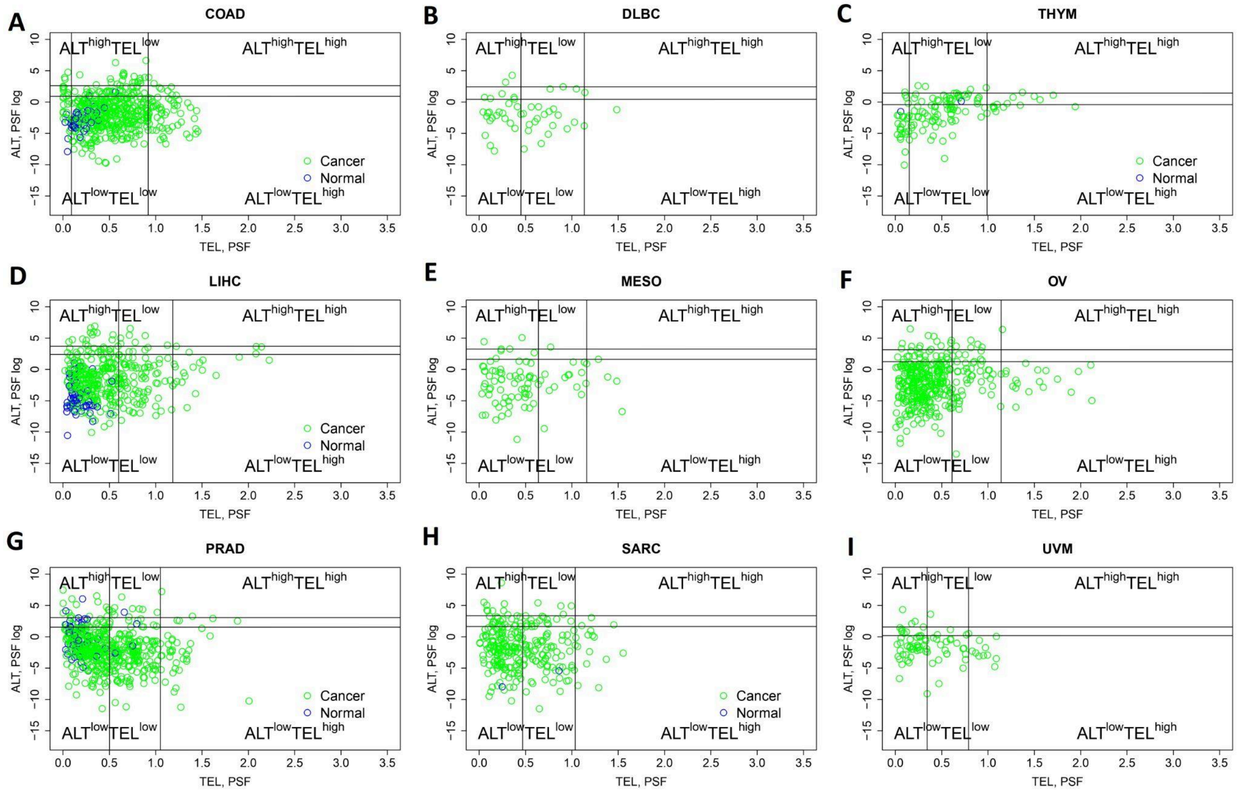

Figure S3. ALT and TEL pathways (**A**) COAD, (**B**) DLBC, (**C**) THYM, (**D**) LIHC, (**E**) MESO, (**F**) OV, (**G**) PRAD, (**H**) SARC, and (**I**) UVM TMM phenotyping. Cancer (green) and normal (blue) samples were plotted based on the TEL PSF and ALT (Log) PSF values. The vertical and horizontal lines reflect the threshold values.

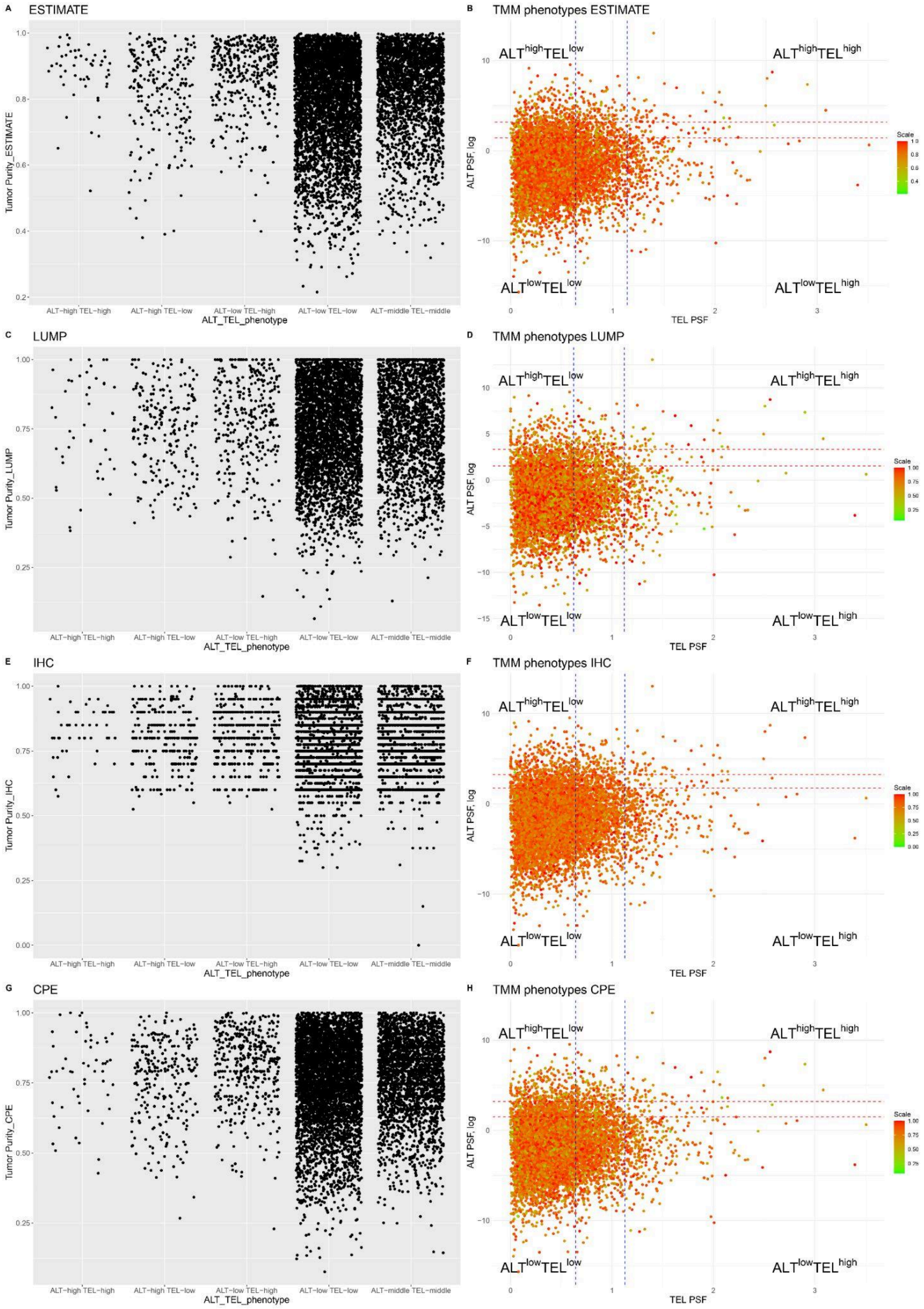
A
ESTIMATE
Tumor Purity_ESTIMATE
ALT_TEL_phenotype
ALT-high TEL-high
ALT-high TEL-low
ALT-low TEL-high
ALT-low TEL-low
ALT-middle TEL-middle
B
TMM phenotypes ESTIMATE
ALT PSF, log
TEL PSF
Scale
C
LUMP
Tumor Purity_LUMP
D
TMM phenotypes LUMP
E
IHC
Tumor Purity_IHC
F
TMM phenotypes IHC
G
CPE
Tumor Purity_CPE
H
TMM phenotypes CPE

Figure S4. Distribution of ALT and TEL pathway tumor purity for 5 phenotypes and tumor purity scale-based TMM phenotyping. TMM space is divided into four groups based on their TEL PSF and ALT (log) PSF values. The vertical and horizontal lines show the thresholds used. The color coding corresponds to the Scale of tumor purity. (**A**) and (**B**) ESTIMATE method. (**C**) and (**D**) LUMP method. (**E**) and (**F**) IHC method. (**G**) and (**H**) CPE method.

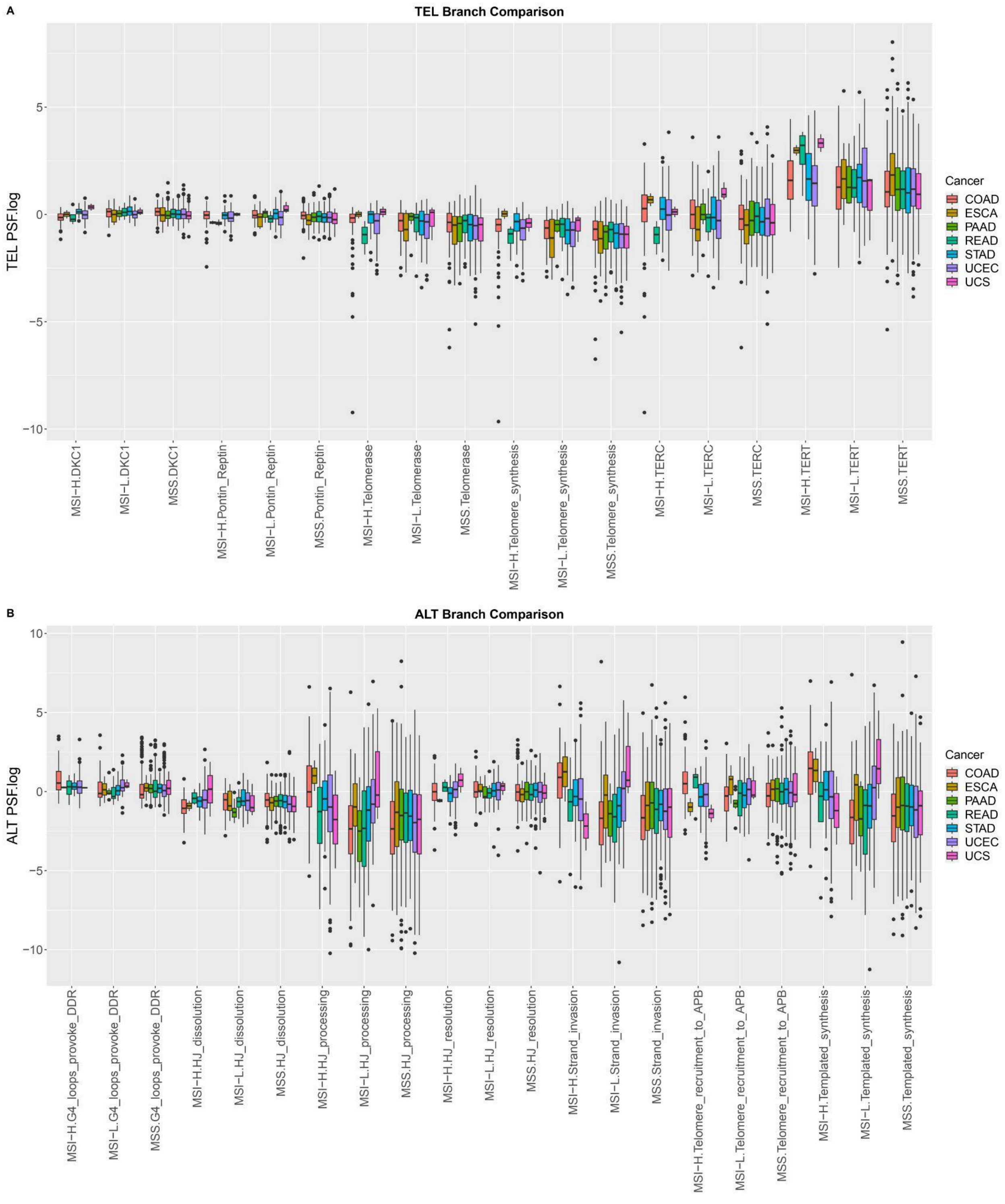

Figure S5. TEL and ALT pathways branch activity pattern for MSS\MSI samples across seven cancer types. **(A)** TEL pathway branch activity in relation to the MSS, MSI-L, and MSI-H status. **(B)** ALT pathway branch activity in relation to the MSS, MSI-L, and MSI-H status. The color coding corresponds to the cancer type. MSI-H samples were missing for the PAAD cancer type. The adjacent normal samples have been excluded from the analysis.

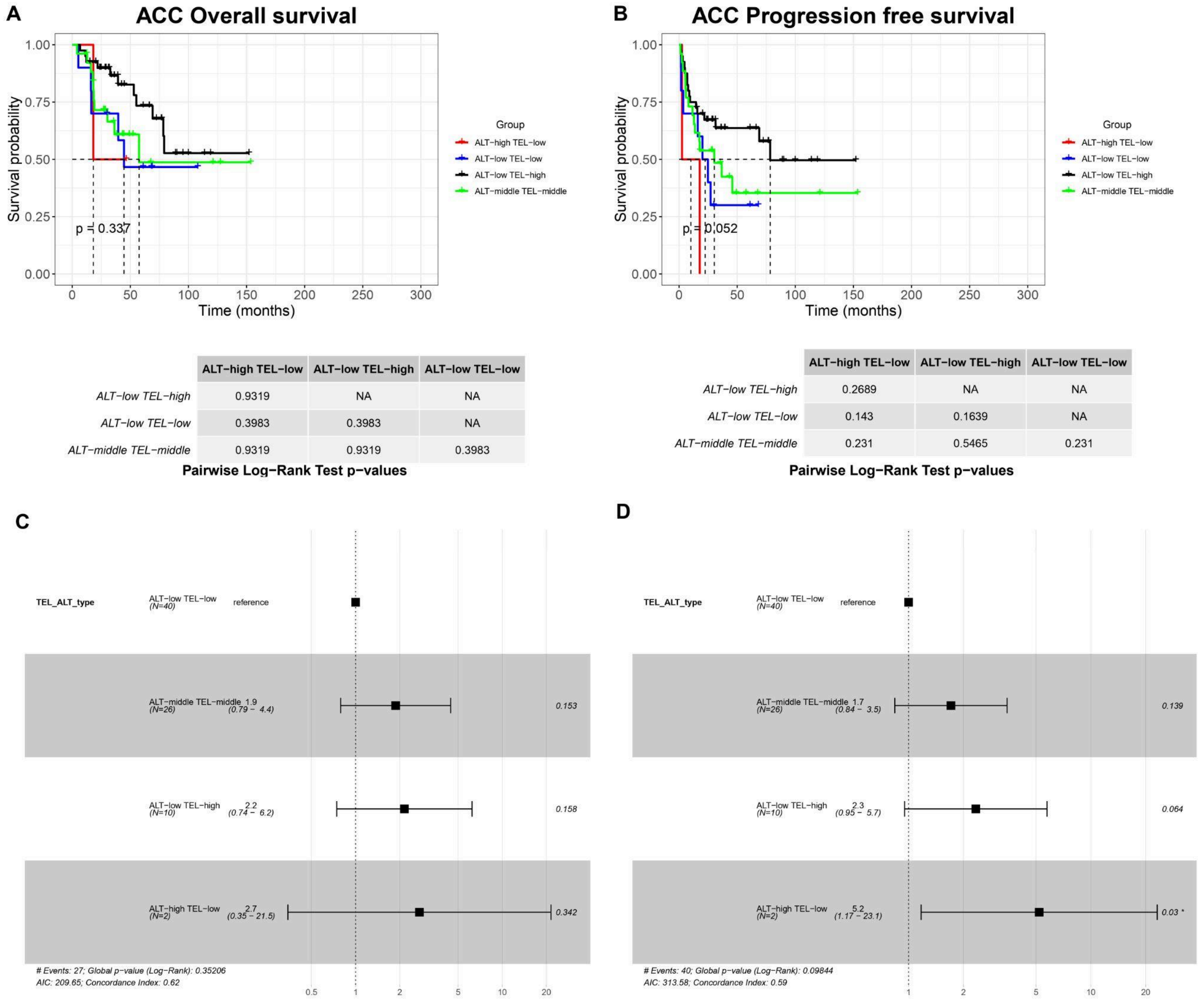

A — Pairwise Log−Rank Test p−values

	ALT−high TEL−low	ALT−low TEL−high	ALT−low TEL−low
ALT−low TEL−high	0.9319	NA	NA
ALT−low TEL−low	0.3983	0.3983	NA
ALT−middle TEL−middle	0.9319	0.9319	0.3983

B — Pairwise Log−Rank Test p−values

	ALT−high TEL−low	ALT−low TEL−high	ALT−low TEL−low
ALT−low TEL−high	0.2689	NA	NA
ALT−low TEL−low	0.143	0.1639	NA
ALT−middle TEL−middle	0.231	0.5465	0.231

Figure S6. Survival and hazard ratio forest plots for ACC ALT and TEL phenotypes (**A**) and (**C**) Overall survival. (**B**) and (**D**) Progression-free survival. Significance was calculated using a Log-rank test for K-M plots and a Cox proportional hazards regression model was used to estimate hazard ratios.The pairwise log-rank test was used to assess the significance between TMM phenotype groups.

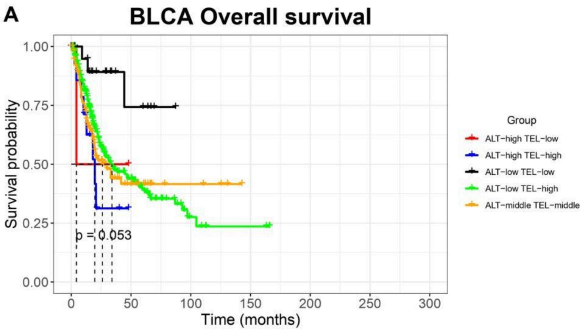

	ALT−high TEL−high	ALT−high TEL−low	ALT−low TEL−high	ALT−low TEL−low
ALT−high TEL−low	0.9838	NA	NA	NA
ALT−low TEL−high	0.4748	0.0333	NA	NA
ALT−low TEL−low	0.9838	0.3121	0.0364	NA
ALT−middle TEL−middle	0.9838	0.6768	0.0364	0.6768

Pairwise Log−Rank Test p−values

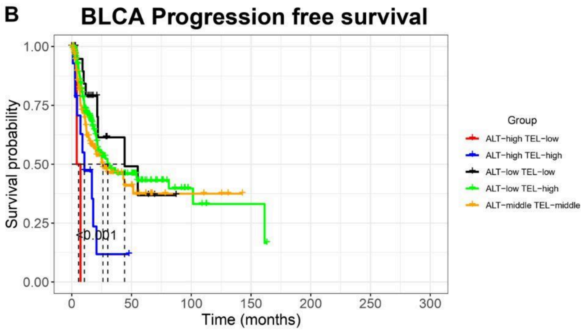

	ALT−high TEL−high	ALT−high TEL−low	ALT−low TEL−high	ALT−low TEL−low
ALT−high TEL−low	0.3114	NA	NA	NA
ALT−low TEL−high	0.0018	0.0083	NA	NA
ALT−low TEL−low	0.0018	0.0043	0.5705	NA
ALT−middle TEL−middle	0.0406	0.0484	0.3116	0.3116

Pairwise Log−Rank Test p−values

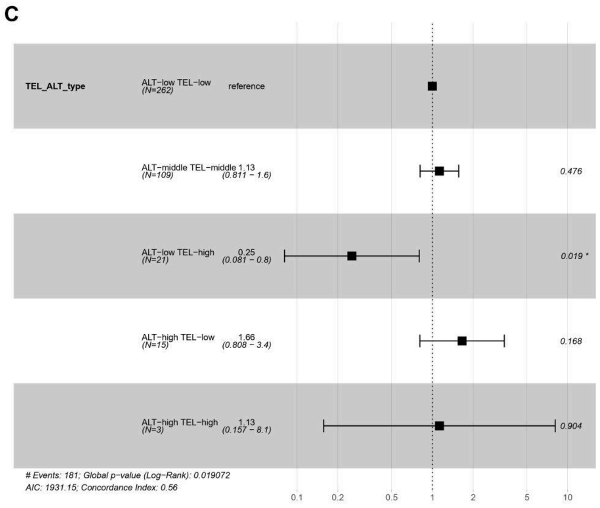

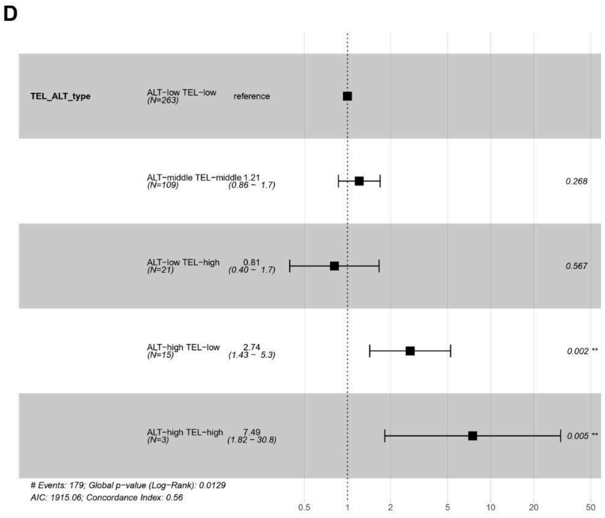

Figure S7. Survival and hazard ratio forest plots for BLCA ALT and TEL phenotypes (**A**) and (**C**) Overall survival. (**B**) and (**D**) Progression-free survival. Significance was calculated using a Log-rank test for K-M plots and a Cox proportional hazards regression model was used to estimate hazard ratios.The pairwise log-rank test was used to assess the significance between TMM phenotype groups.

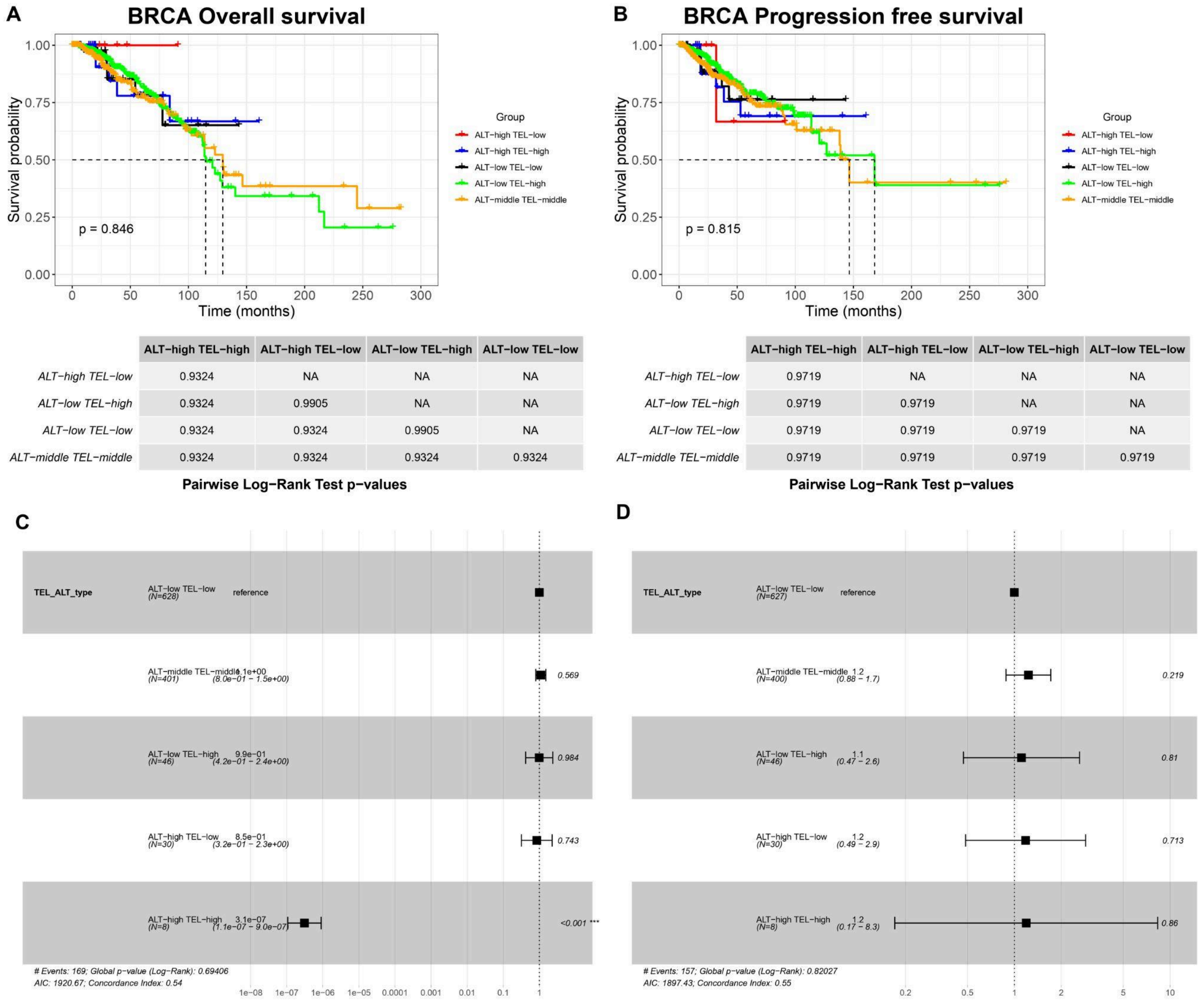

Figure S8. Survival and hazard ratio forest plots for BRCA ALT and TEL phenotypes (**A**) and (**C**) Overall survival. (**B**) and (**D**) Progression-free survival. Significance was calculated using a Log-rank test for K-M plots and a Cox proportional hazards regression model was used to estimate hazard ratios.The pairwise log-rank test was used to assess the significance between TMM phenotype groups.

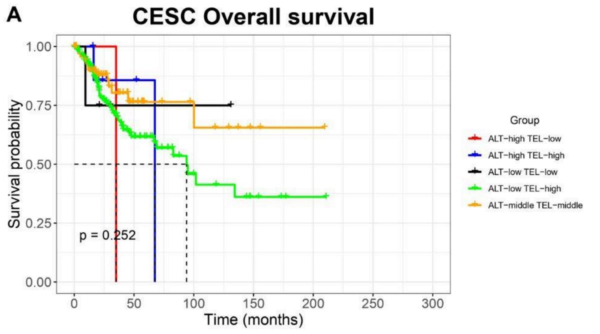

	ALT−high TEL−high	ALT−high TEL−low	ALT−low TEL−high	ALT−low TEL−low
ALT−high TEL−low	0.8672	NA	NA	NA
ALT−low TEL−high	0.9408	0.9408	NA	NA
ALT−low TEL−low	0.8672	0.9408	0.9408	NA
ALT−middle TEL−middle	0.6184	0.9408	0.9408	0.5183

Pairwise Log−Rank Test p−values

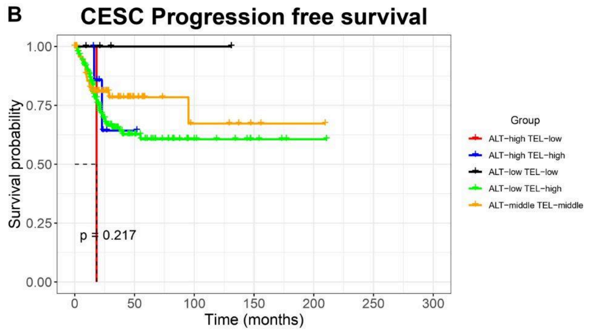

	ALT−high TEL−high	ALT−high TEL−low	ALT−low TEL−high	ALT−low TEL−low
ALT−high TEL−low	0.3273	NA	NA	NA
ALT−low TEL−high	0.3273	0.4192	NA	NA
ALT−low TEL−low	0.3273	0.8107	0.4192	NA
ALT−middle TEL−middle	0.3273	0.8263	0.4192	0.3273

Pairwise Log−Rank Test p−values

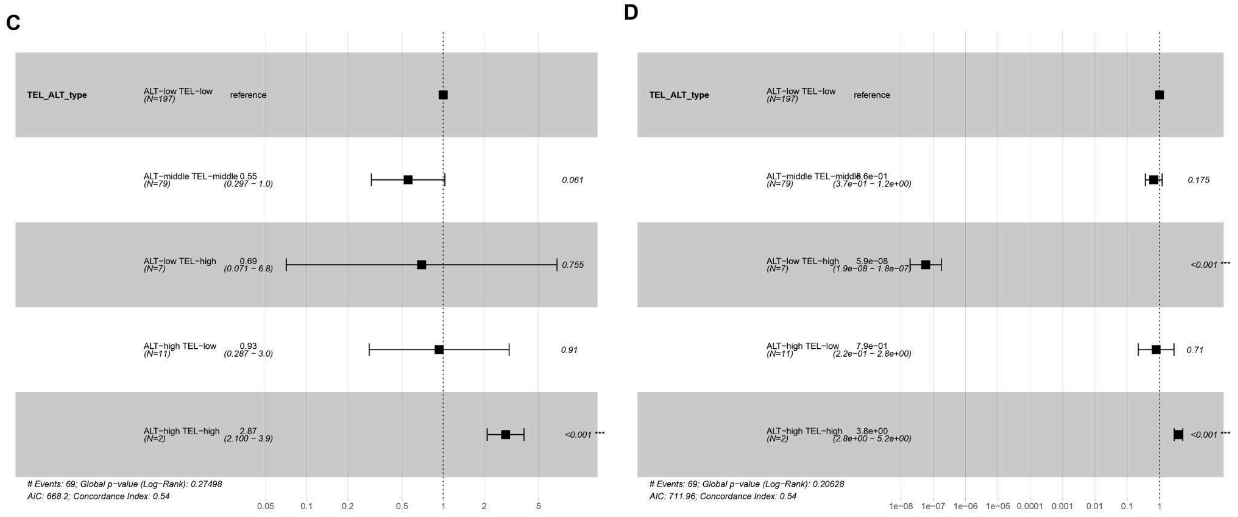

Figure S9. Survival and hazard ratio forest plots for CESC ALT and TEL phenotypes (**A**) and (**C**) Overall survival. (**B**) and (**D**) Progression-free survival. Significance was calculated using a Log-rank test for K-M plots and a Cox proportional hazards regression model was used to estimate hazard ratios. The pairwise log-rank test was used to assess the significance between TMM phenotype groups.

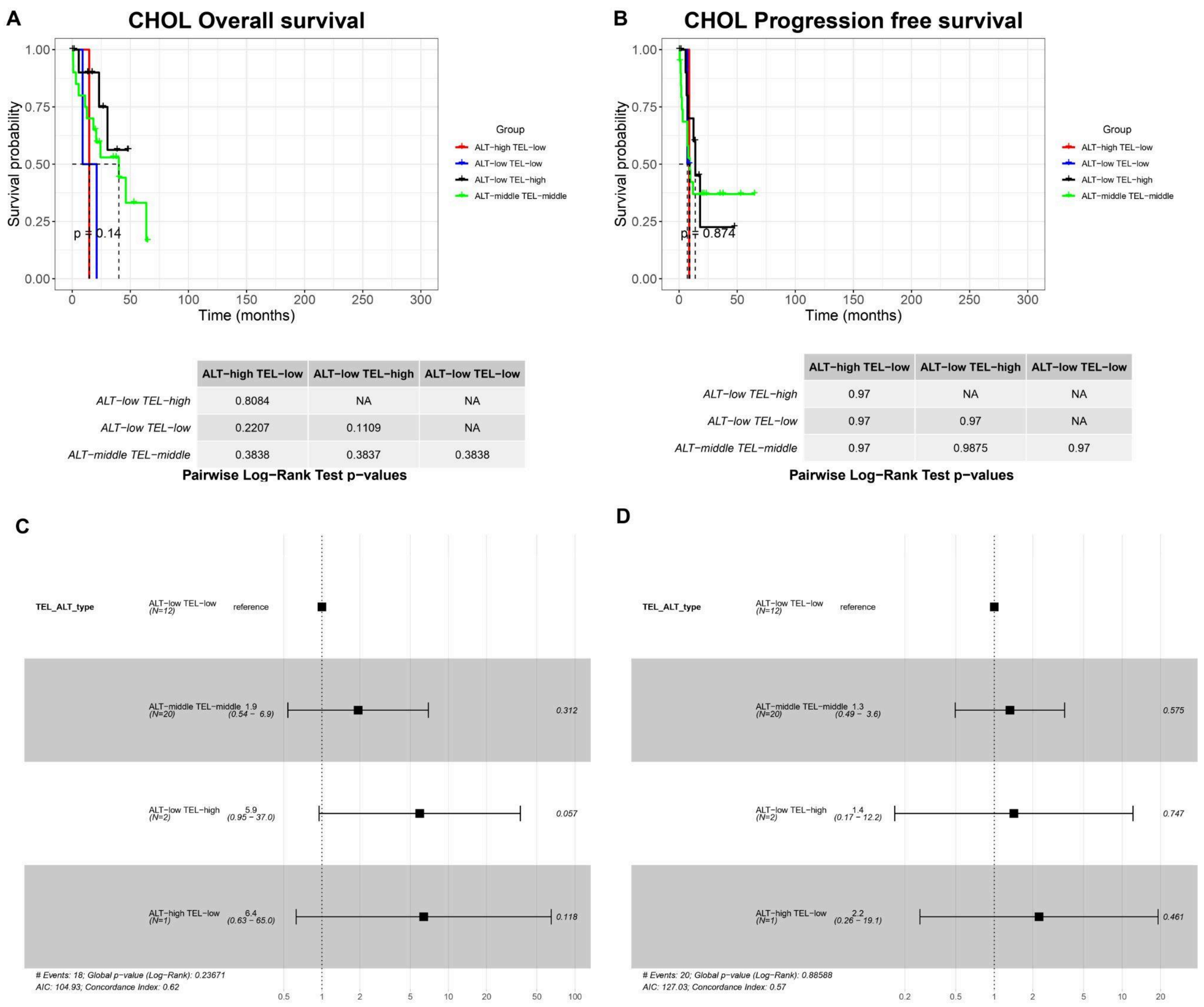

Figure S10. Survival and hazard ratio forest plots for CHOL ALT and TEL phenotypes (**A**) and (**C**) Overall survival. (**B**) and (**D**) Progression-free survival. Significance was calculated using a Log-rank test for K-M plots and a Cox proportional hazards regression model was used to estimate hazard ratios. The pairwise log-rank test was used to assess the significance between TMM phenotype groups.

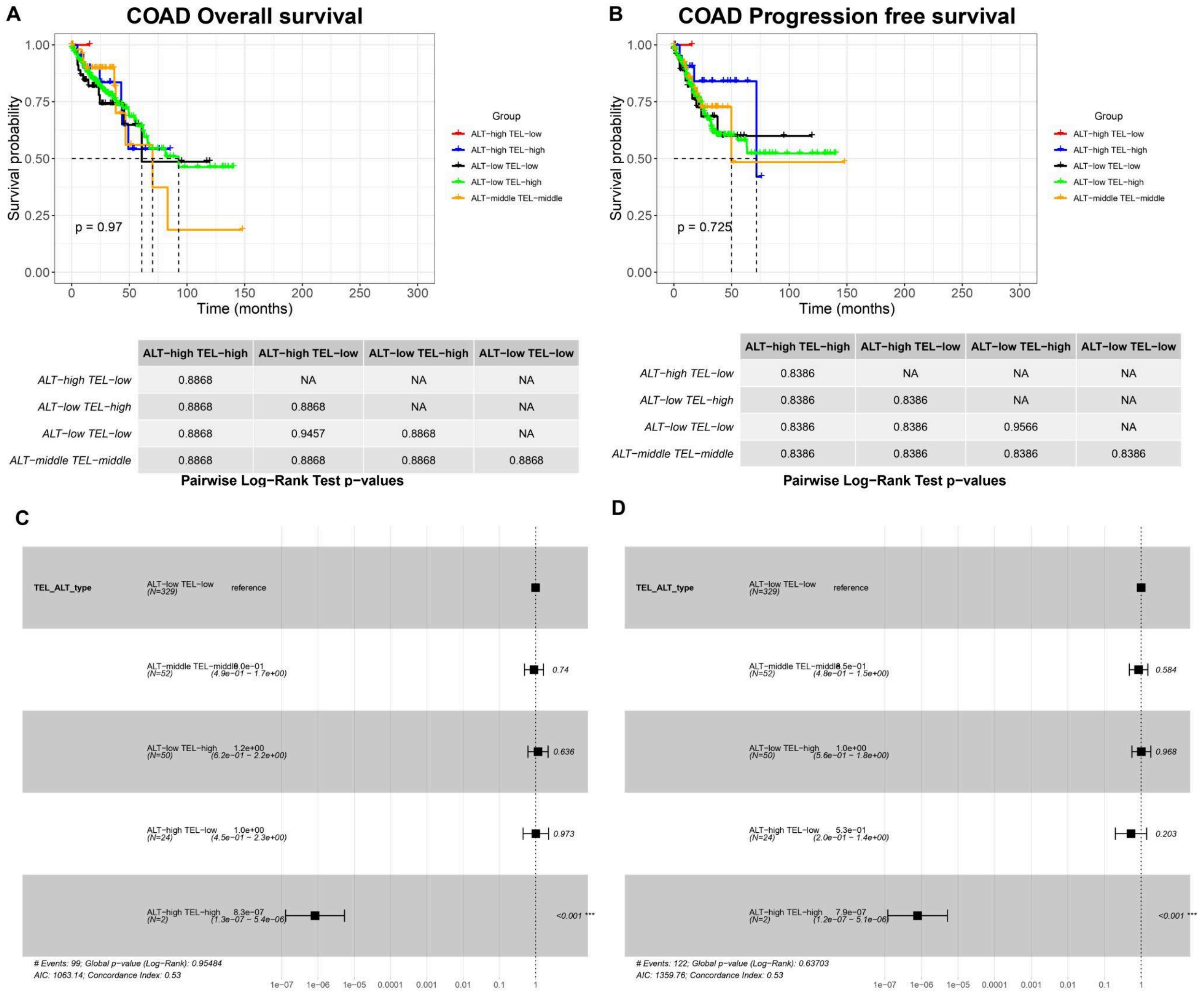

Figure S11. Survival and hazard ratio forest plots for COAD ALT and TEL phenotypes (**A**) and (**C**) Overall survival. (**B**) and (**D**) Progression-free survival. Significance was calculated using a Log-rank test for K-M plots and a Cox proportional hazards regression model was used to estimate hazard ratios. The pairwise log-rank test was used to assess the significance between TMM phenotype groups.

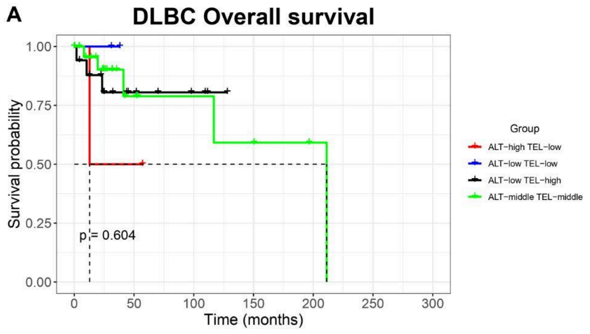

	ALT−high TEL−low	ALT−low TEL−high	ALT−low TEL−low
ALT−low TEL−high	0.7214	NA	NA
ALT−low TEL−low	0.7214	0.7773	NA
ALT−middle TEL−middle	0.7214	0.7837	0.8641

Pairwise Log−Rank Test p−values

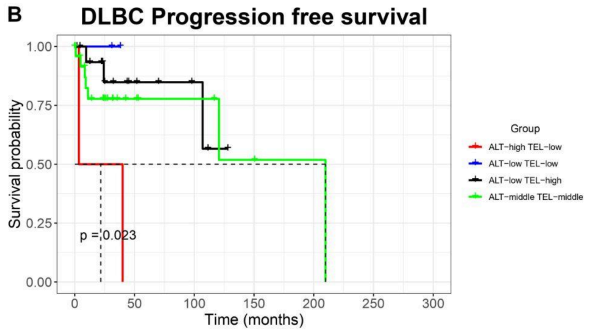

	ALT−high TEL−low	ALT−low TEL−high	ALT−low TEL−low
ALT−low TEL−high	0.6327	NA	NA
ALT−low TEL−low	0.0279	0.6327	NA
ALT−middle TEL−middle	0.0639	0.6327	0.6327

Pairwise Log−Rank Test p−values

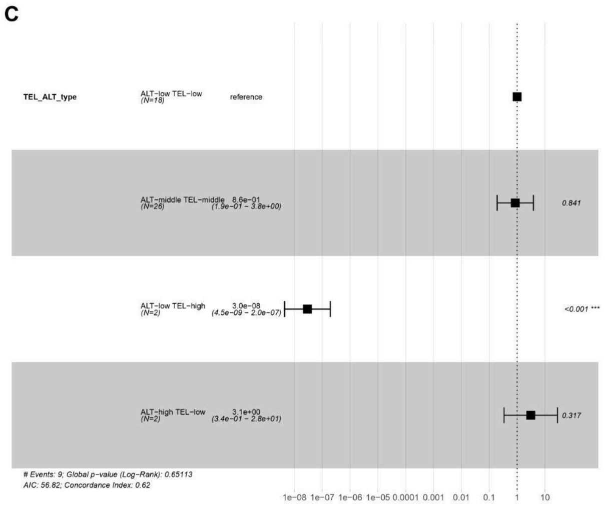

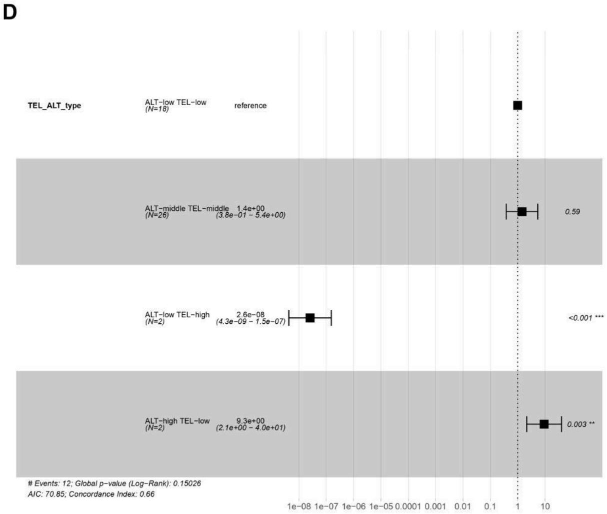

Figure S12. Survival and hazard ratio forest plots for DLBC ALT and TEL phenotypes (**A**) and (**C**) Overall survival. (**B**) and (**D**) Progression-free survival. Significance was calculated using a Log-rank test for K-M plots and a Cox proportional hazards regression model was used to estimate hazard ratios.The pairwise log-rank test was used to assess the significance between TMM phenotype groups.

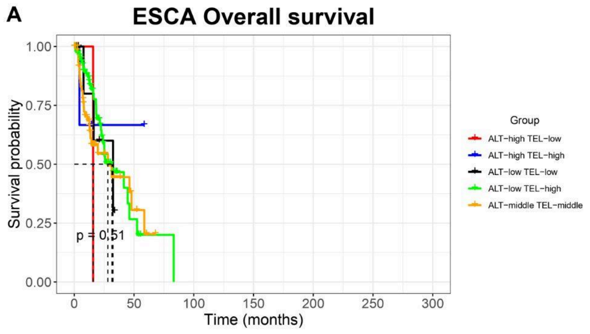

	ALT−high TEL−high	ALT−high TEL−low	ALT−low TEL−high	ALT−low TEL−low
ALT−high TEL−low	0.8818	NA	NA	NA
ALT−low TEL−high	0.8223	0.9227	NA	NA
ALT−low TEL−low	0.8223	0.8818	0.8818	NA
ALT−middle TEL−middle	0.8818	0.8818	0.9232	0.8223

Pairwise Log−Rank Test p−values

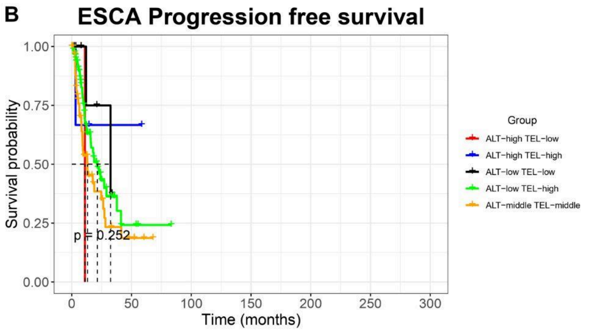

	ALT−high TEL−high	ALT−high TEL−low	ALT−low TEL−high	ALT−low TEL−low
ALT−high TEL−low	0.6688	NA	NA	NA
ALT−low TEL−high	0.4113	1	NA	NA
ALT−low TEL−low	0.6688	0.6688	0.6688	NA
ALT−middle TEL−middle	0.7134	0.6688	0.638	0.4113

Pairwise Log−Rank Test p−values

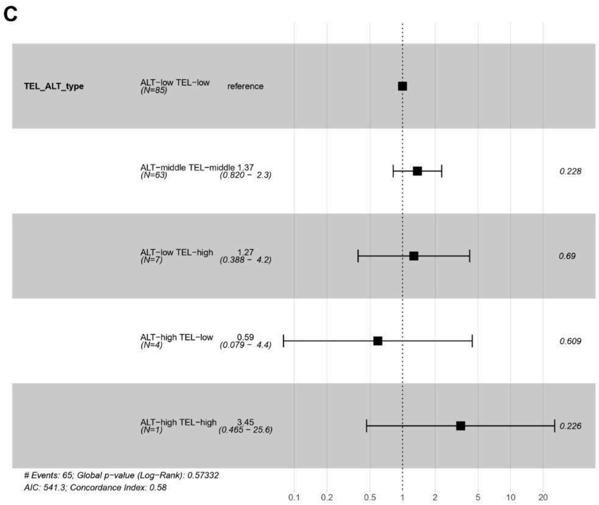

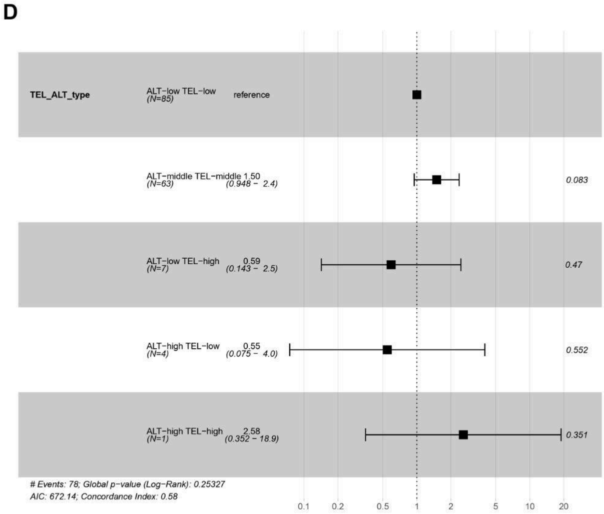

Figure S13. Survival and hazard ratio forest plots for ESCA ALT and TEL phenotypes (**A**) and (**C**) Overall survival. (**B**) and (**D**) Progression-free survival. Significance was calculated using a Log-rank test for K-M plots and a Cox proportional hazards regression model was used to estimate hazard ratios.The pairwise log-rank test was used to assess the significance between TMM phenotype groups.

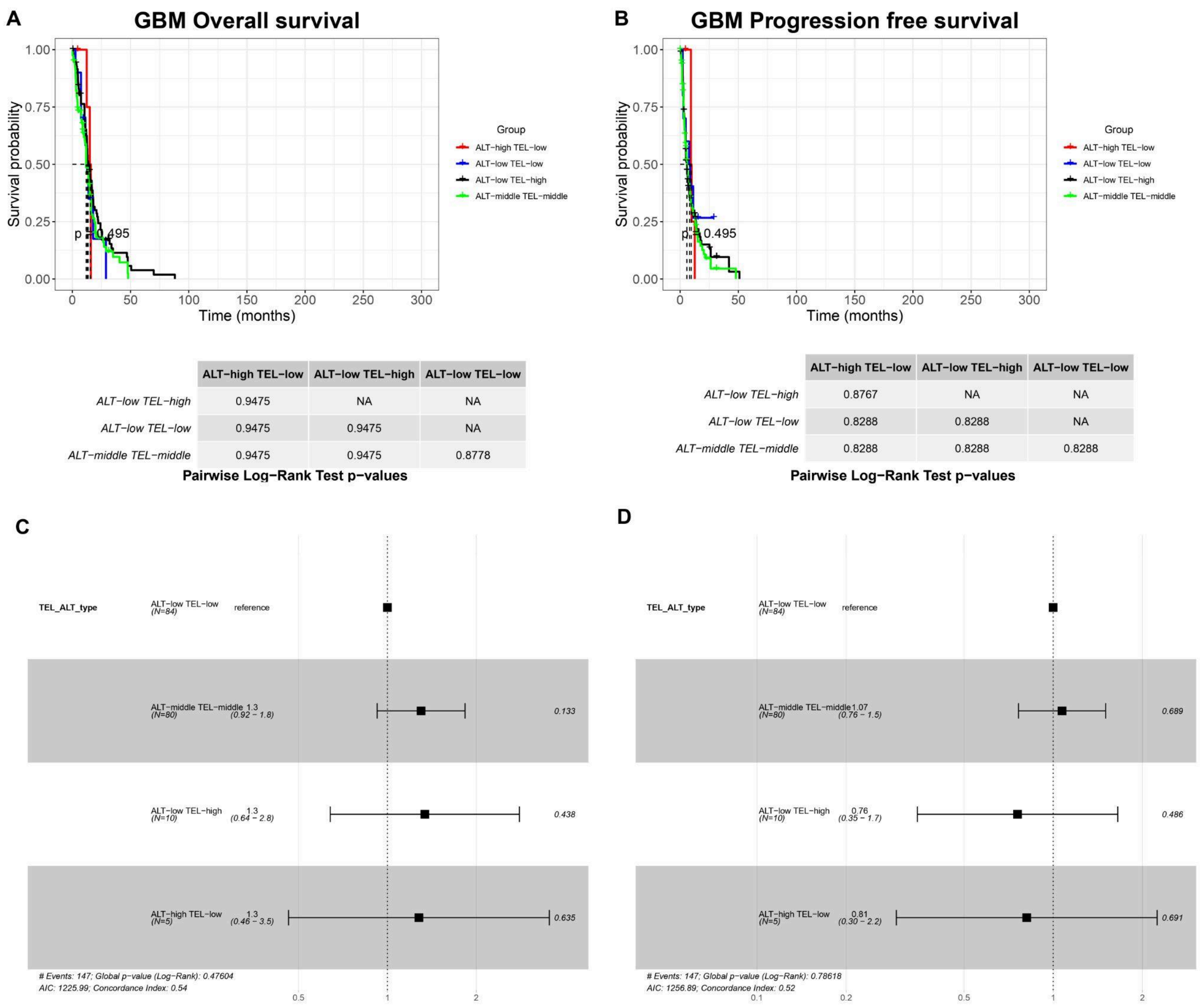

Figure S14. Survival and hazard ratio forest plots for GBM ALT and TEL phenotypes (**A**) and (**C**) Overall survival. (**B**) and (**D**) Progression-free survival. Significance was calculated using a Log-rank test for K-M plots and a Cox proportional hazards regression model was used to estimate hazard ratios.The pairwise log-rank test was used to assess the significance between TMM phenotype groups.

A

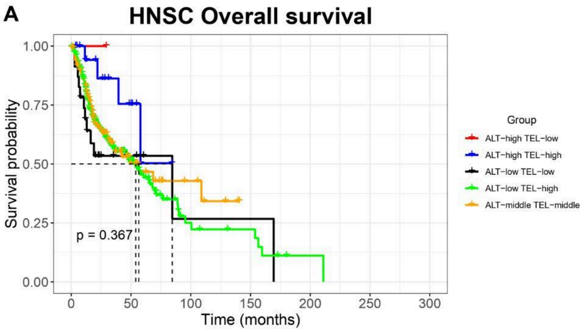

	ALT−high TEL−high	ALT−high TEL−low	ALT−low TEL−high	ALT−low TEL−low
ALT−high TEL−low	0.7062	NA	NA	NA
ALT−low TEL−high	0.6232	0.3322	NA	NA
ALT−low TEL−low	0.6232	0.3322	0.6232	NA
ALT−middle TEL−middle	0.6232	0.3322	0.6232	0.7062

Pairwise Log−Rank Test p−values

B

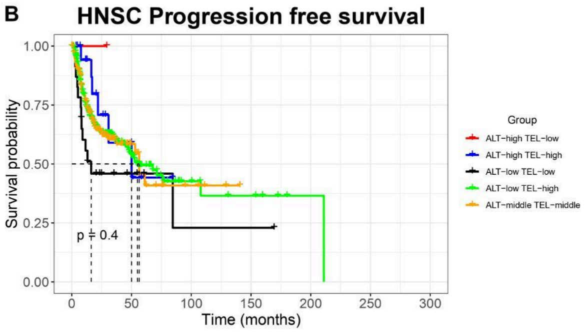

	ALT−high TEL−high	ALT−high TEL−low	ALT−low TEL−high	ALT−low TEL−low
ALT−high TEL−low	0.6285	NA	NA	NA
ALT−low TEL−high	0.6208	0.4206	NA	NA
ALT−low TEL−low	0.6208	0.6208	0.4206	NA
ALT−middle TEL−middle	0.6208	0.6208	0.4206	0.9166

HNSC Pairwise Log−Rank Test p−values

C

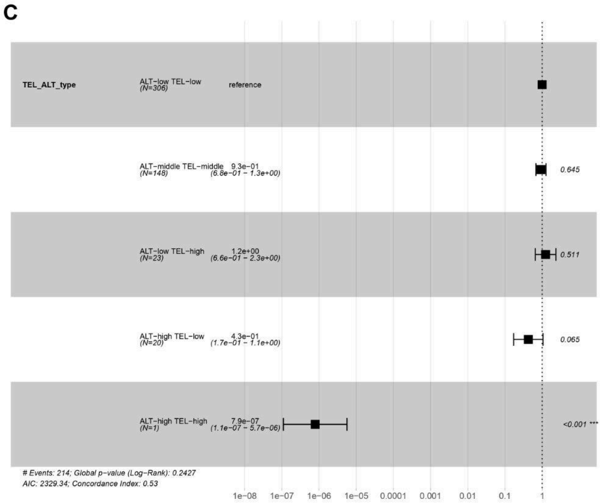

D

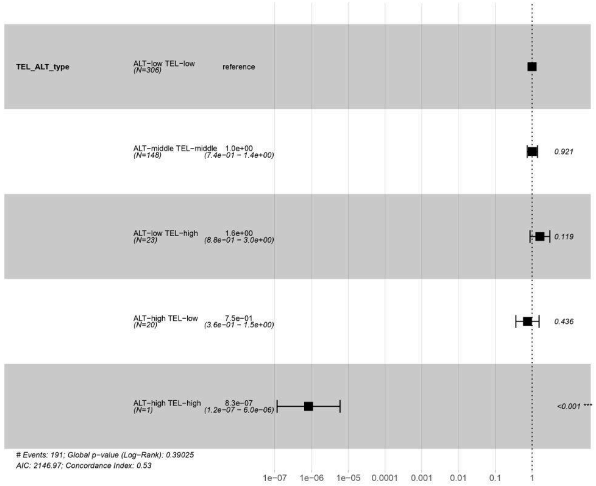

Figure S15. Survival and hazard ratio forest plots for HNSC ALT and TEL phenotypes (**A**) and (**C**) Overall survival. (**B**) and (**D**) Progression-free survival. Significance was calculated using a Log-rank test for K-M plots and a Cox proportional hazards regression model was used to estimate hazard ratios.The pairwise log-rank test was used to assess the significance between TMM phenotype groups.

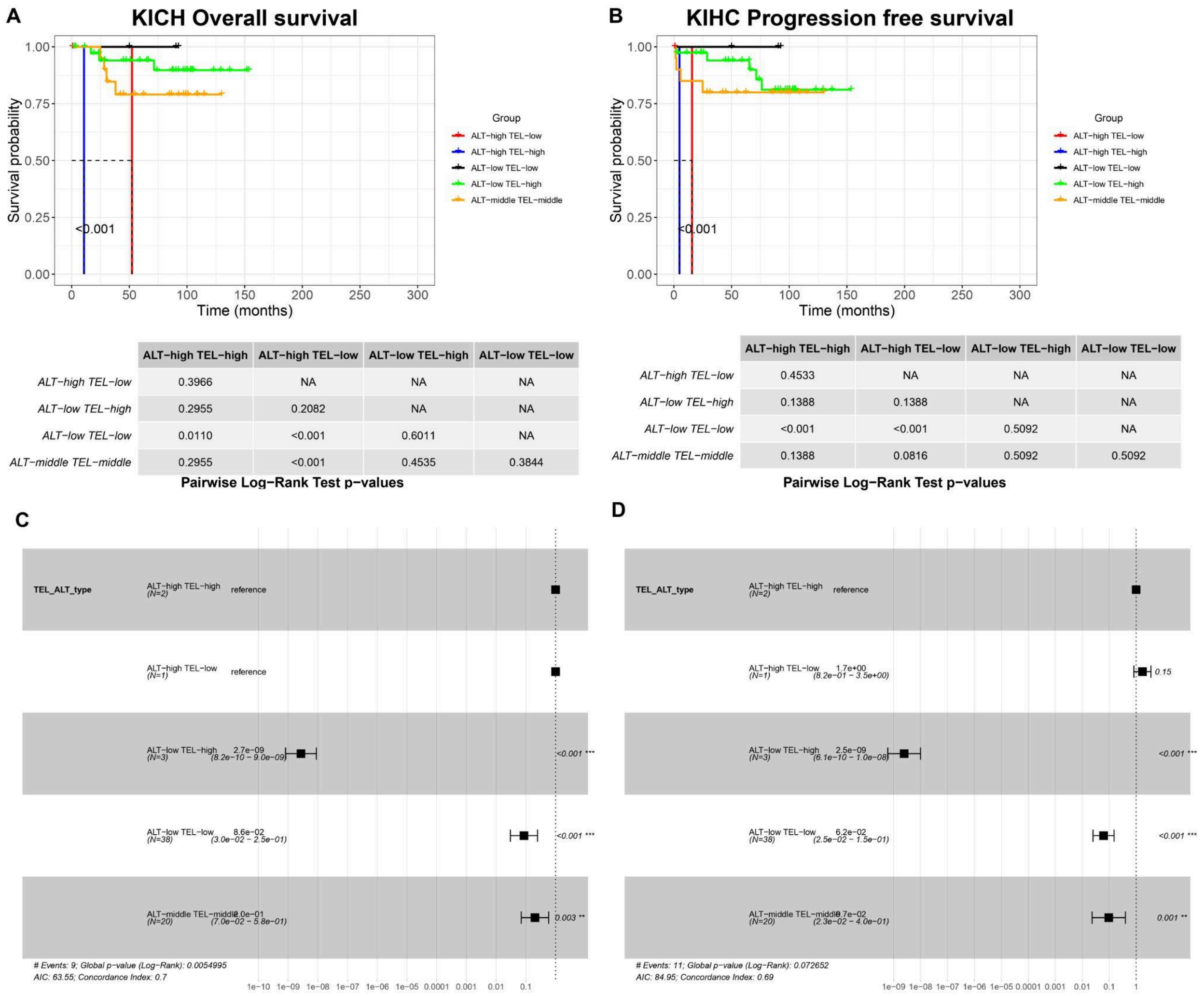

Figure S16. Survival and hazard ratio forest plots for KICH ALT and TEL phenotypes (**A**) and (**C**) Overall survival. (**B**) and (**D**) Progression-free survival. Significance was calculated using a Log-rank test for K-M plots and a Cox proportional hazards regression model was used to estimate hazard ratios.The pairwise log-rank test was used to assess the significance between TMM phenotype groups.

A

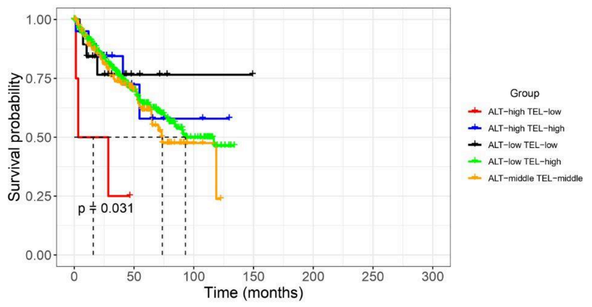

	ALT−high TEL−high	ALT−high TEL−low	ALT−low TEL−high	ALT−low TEL−low
ALT−high TEL−low	0.066	NA	NA	NA
ALT−low TEL−high	0.066	0.9081	NA	NA
ALT−low TEL−low	0.0182	0.9081	0.9081	NA
ALT−middle TEL−middle	0.0182	0.782	0.782	0.782

Pairwise Log−Rank Test p−values

B

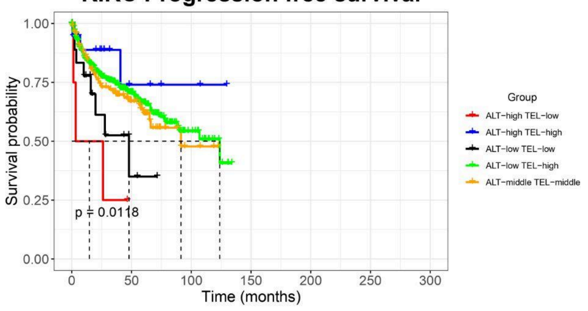

	ALT−high TEL−high	ALT−high TEL−low	ALT−low TEL−high	ALT−low TEL−low
ALT−high TEL−low	0.061	NA	NA	NA
ALT−low TEL−high	0.268	0.132	NA	NA
ALT−low TEL−low	0.049	0.303	0.127	NA
ALT−middle TEL−middle	0.061	0.268	0.19	0.53

Pairwise Log−Rank Test p−values

C

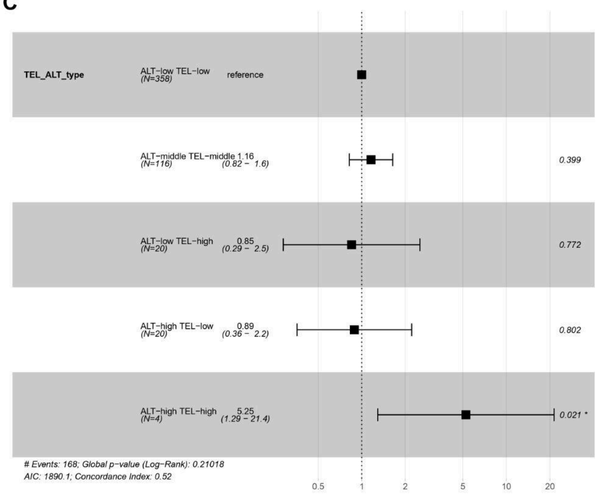

D

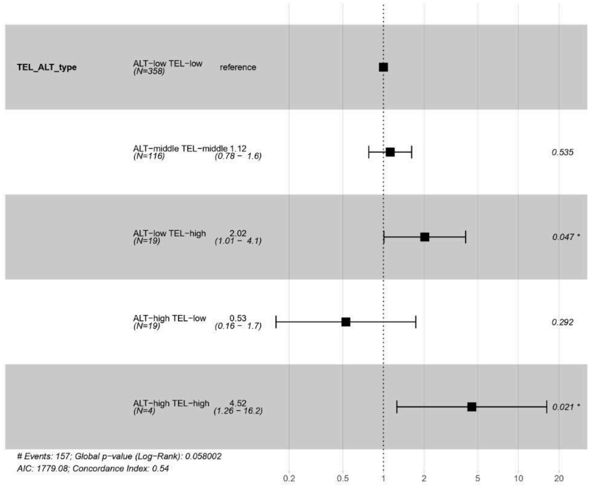

Figure S17. Survival and hazard ratio forest plots for KIRC ALT and TEL phenotypes (**A**) and (**C**) Overall survival. (**B**) and (**D**) Progression-free survival. Significance was calculated using a Log-rank test for K-M plots and a Cox proportional hazards regression model was used to estimate hazard ratios.The pairwise log-rank test was used to assess the significance between TMM phenotype groups.

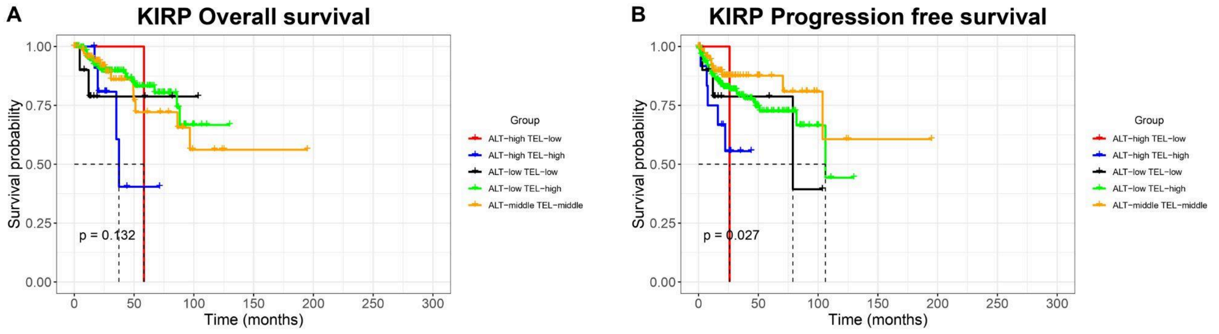

	ALT−high TEL−high	ALT−high TEL−low	ALT−low TEL−high	ALT−low TEL−low
ALT−high TEL−low	0.8787	NA	NA	NA
ALT−low TEL−high	0.7256	0.8787	NA	NA
ALT−low TEL−low	0.2672	0.2672	0.7256	NA
ALT−middle TEL−middle	0.7256	0.2672	0.8756	0.8231

Pairwise Log−Rank Test p−values

	ALT−high TEL−high	ALT−high TEL−low	ALT−low TEL−high	ALT−low TEL−low
ALT−high TEL−low	0.6934	NA	NA	NA
ALT−low TEL−high	0.5242	0.5443	NA	NA
ALT−low TEL−low	0.1829	0.1584	0.5463	NA
ALT−middle TEL−middle	0.1584	0.0959	0.2687	0.2687

Pairwise Log−Rank Test p−values

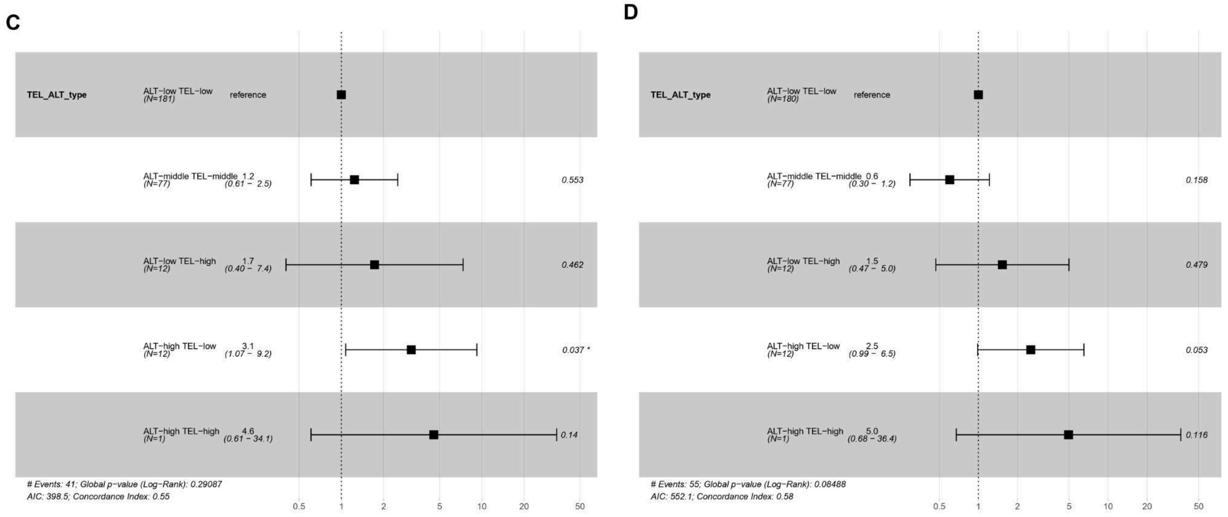

Figure S18. Survival and hazard ratio forest plots for KIRP ALT and TEL phenotypes (**A**) and (**C**) Overall survival. (**B**) and (**D**) Progression-free survival. Significance was calculated using a Log-rank test for K-M plots and a Cox proportional hazards regression model was used to estimate hazard ratios.The pairwise log-rank test was used to assess the significance between TMM phenotype groups.

A

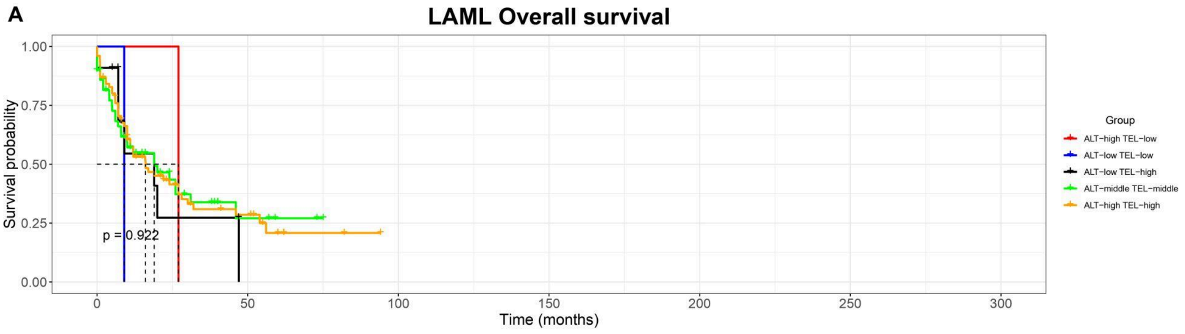

	ALT−high TEL−high	ALT−high TEL−low	ALT−low TEL−high	ALT−low TEL−low
ALT−high TEL−low	0.9925	NA	NA	NA
ALT−low TEL−high	0.9925	0.9925	NA	NA
ALT−low TEL−low	0.9925	0.9925	0.9925	NA
ALT−middle TEL−middle	0.9925	0.9925	0.9925	0.9925

Pairwise Log−Rank Test p−values

B

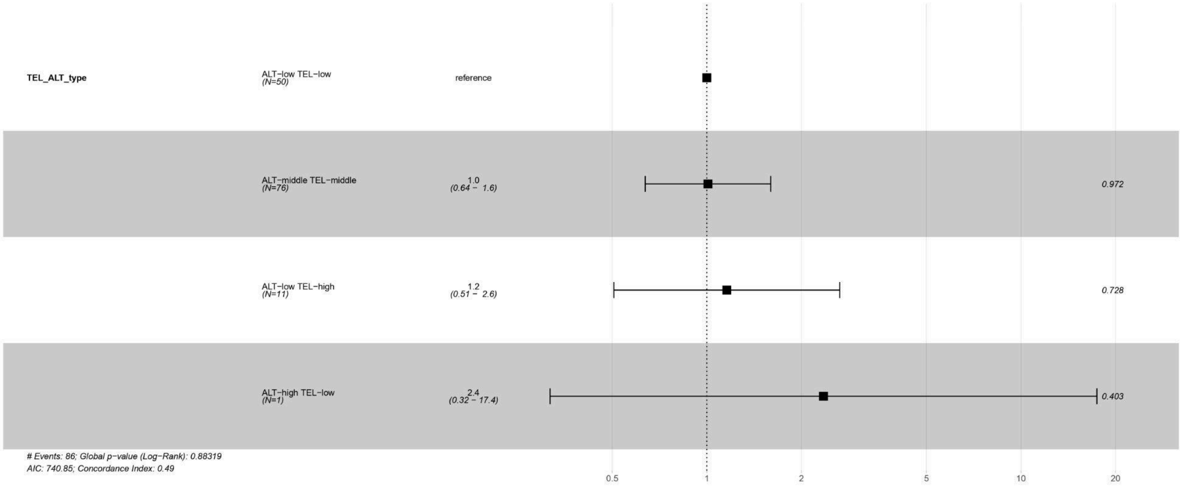

Figure S19. Survival and hazard ratio forest plots for LAML ALT and TEL phenotypes (**A**) and (**B**) Overall survival. Significance was calculated using a Log-rank test for K-M plots and a Cox proportional hazards regression model was used to estimate hazard ratios.The pairwise log-rank test was used to assess the significance between TMM phenotype groups.

A

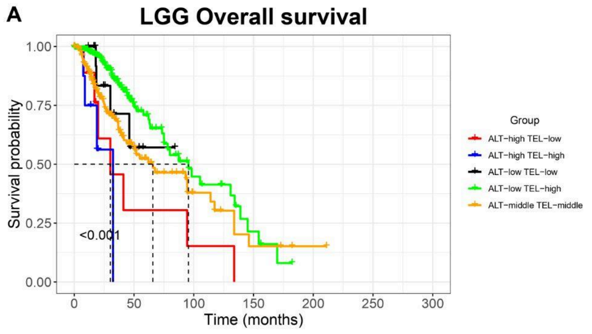

B

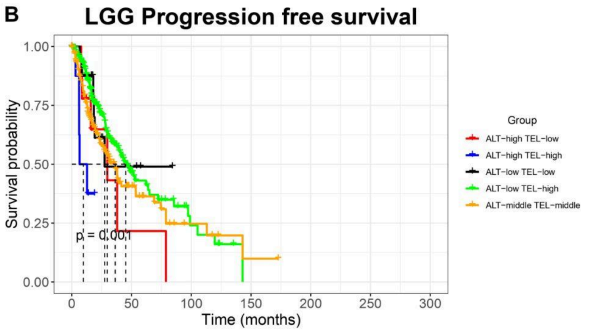

	ALT−high TEL−high	ALT−high TEL−low	ALT−low TEL−high	ALT−low TEL−low
ALT−high TEL−low	0.4631	NA	NA	NA
ALT−low TEL−high	0.2442	0.0812	NA	NA
ALT−low TEL−low	0.0066	<0.001	0.4345	NA
ALT−middle TEL−middle	0.2442	0.0812	0.4978	0.0027

LGG Pairwise Log−Rank Test p−values

	ALT−high TEL−high	ALT−high TEL−low	ALT−low TEL−high	ALT−low TEL−low
ALT−high TEL−low	0.1753	NA	NA	NA
ALT−low TEL−high	0.3816	0.0585	NA	NA
ALT−low TEL−low	0.1753	<0.001	0.9498	NA
ALT−middle TEL−middle	0.5722	0.0585	0.4384	0.0585

Pairwise Log−Rank Test p−values

C

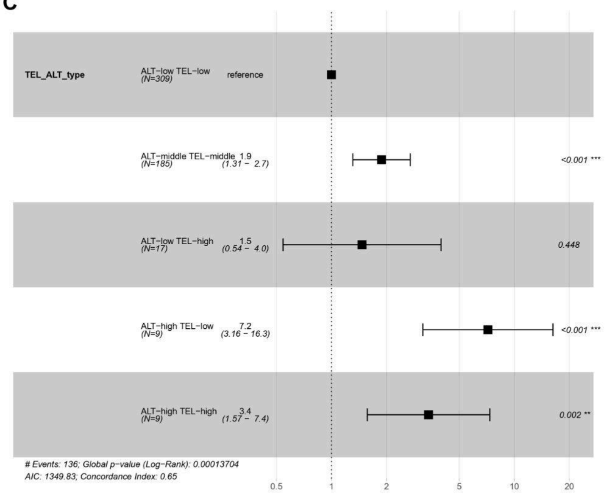

D

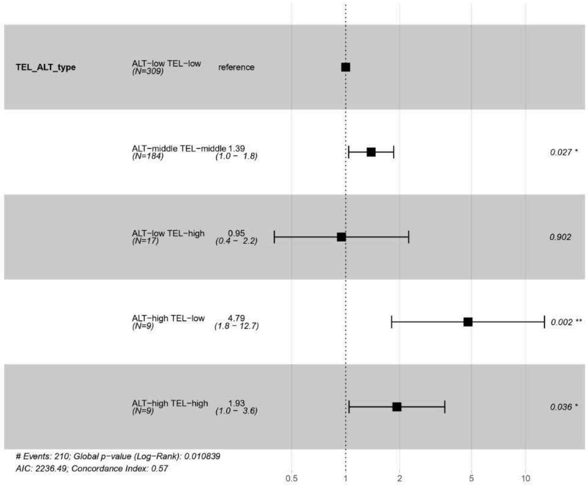

Figure S20. Survival and hazard ratio forest plots for LGG ALT and TEL phenotypes (**A**) and (**C**) Overall survival. (**B**) and (**D**) Progression-free survival. Significance was calculated using a Log-rank test for K-M plots and a Cox proportional hazards regression model was used to estimate hazard ratios.The pairwise log-rank test was used to assess the significance between TMM phenotype groups.

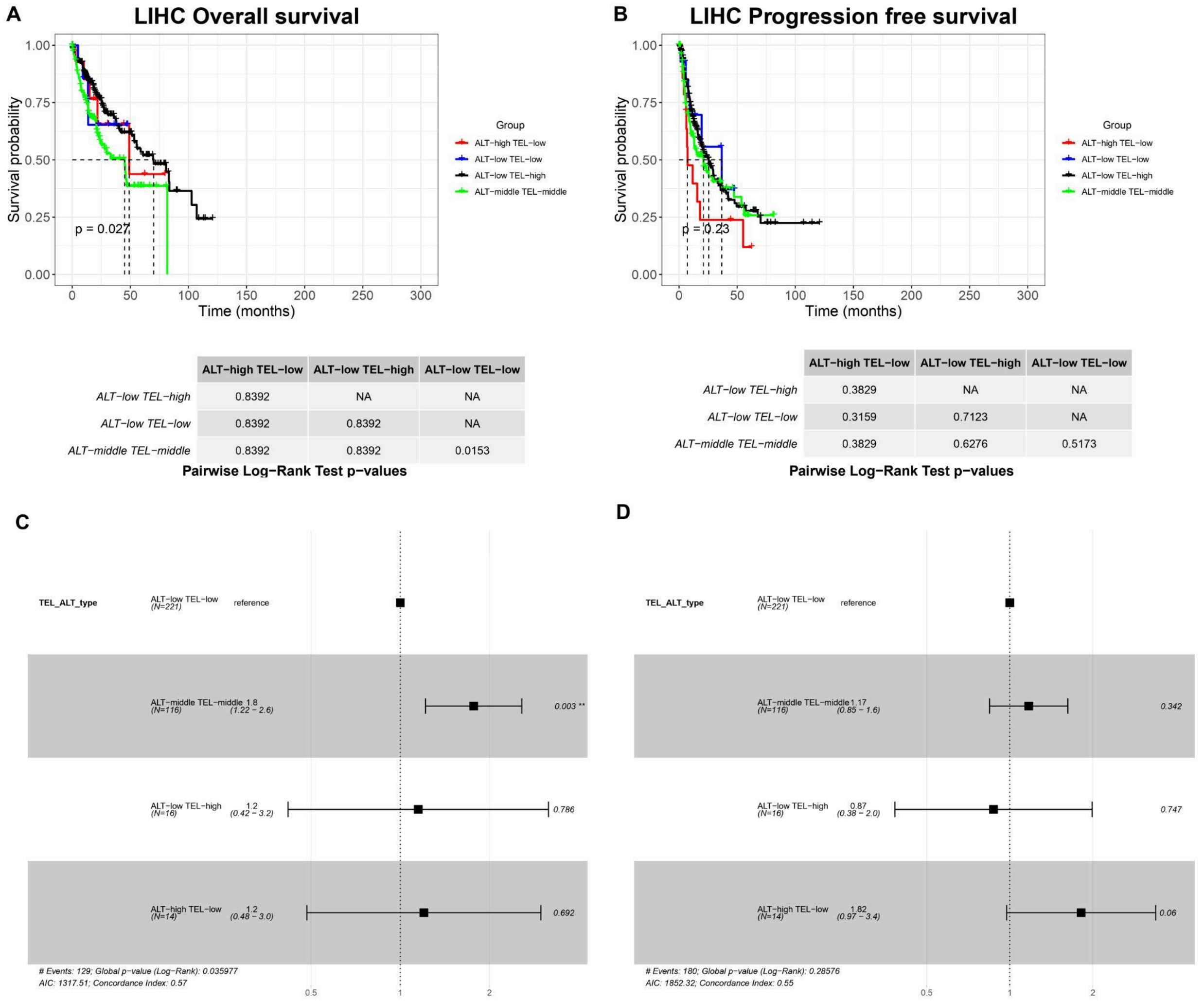

	ALT−high TEL−low	ALT−low TEL−high	ALT−low TEL−low
ALT−low TEL−high	0.8392	NA	NA
ALT−low TEL−low	0.8392	0.8392	NA
ALT−middle TEL−middle	0.8392	0.8392	0.0153

Pairwise Log−Rank Test p−values

	ALT−high TEL−low	ALT−low TEL−high	ALT−low TEL−low
ALT−low TEL−high	0.3829	NA	NA
ALT−low TEL−low	0.3159	0.7123	NA
ALT−middle TEL−middle	0.3829	0.6276	0.5173

Pairwise Log−Rank Test p−values

Figure S21. Survival and hazard ratio forest plots for LIHC ALT and TEL phenotypes (**A**) and (**C**) Overall survival. (**B**) and (**D**) Progression-free survival. Significance was calculated using a Log-rank test for K-M plots and a Cox proportional hazards regression model was used to estimate hazard ratios.The pairwise log-rank test was used to assess the significance between TMM phenotype groups.

A

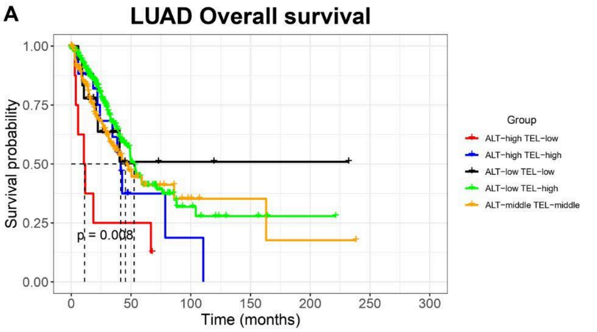

	ALT−high TEL−high	ALT−high TEL−low	ALT−low TEL−high	ALT−low TEL−low
ALT−high TEL−low	0.1534	NA	NA	NA
ALT−low TEL−high	0.0829	0.6134	NA	NA
ALT−low TEL−low	0.0154	0.2912	0.912	NA
ALT−middle TEL−middle	0.0563	0.6911	0.6911	0.1616

Pairwise Log−Rank Test p−values

B

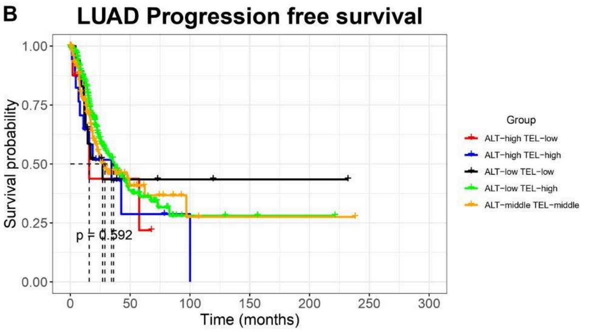

	ALT−high TEL−high	ALT−high TEL−low	ALT−low TEL−high	ALT−low TEL−low
ALT−high TEL−low	0.961	NA	NA	NA
ALT−low TEL−high	0.8571	0.8571	NA	NA
ALT−low TEL−low	0.8571	0.8571	0.9354	NA
ALT−middle TEL−middle	0.8571	0.8571	0.961	0.8571

Pairwise Log−Rank Test p−values

C

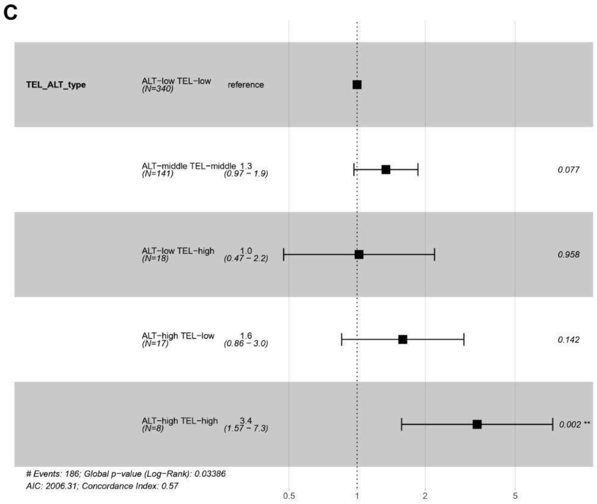

D

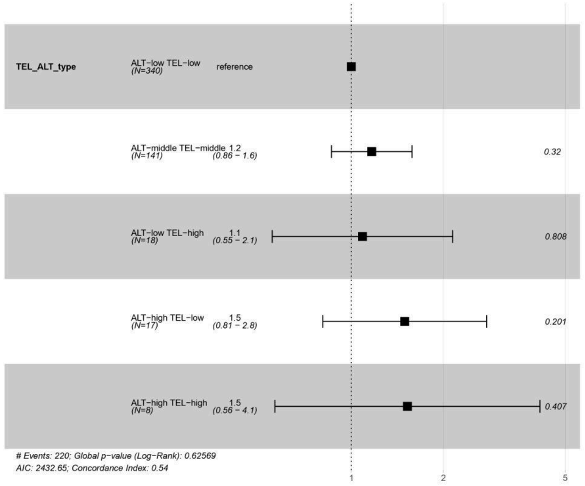

Figure S22. Survival and hazard ratio forest plots for LUAD ALT and TEL phenotypes (**A**) and (**C**) Overall survival. (**B**) and (**D**) Progression-free survival. Significance was calculated using a Log-rank test for K-M plots and a Cox proportional hazards regression model was used to estimate hazard ratios.The pairwise log-rank test was used to assess the significance between TMM phenotype groups.

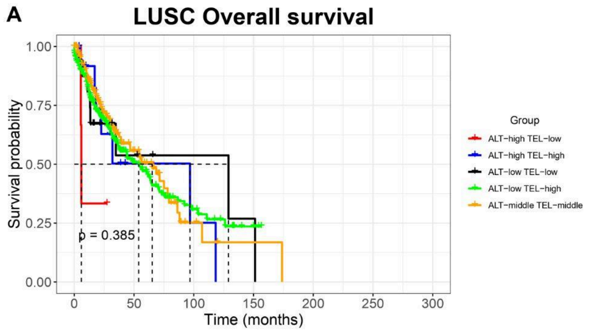

	ALT−high TEL−high	ALT−high TEL−low	ALT−low TEL−high	ALT−low TEL−low
ALT−high TEL−low	0.4868	NA	NA	NA
ALT−low TEL−high	0.411	0.9797	NA	NA
ALT−low TEL−low	0.411	0.9797	0.9797	NA
ALT−middle TEL−middle	0.2834	0.9797	0.9797	0.9678

Pairwise Log−Rank Test p−values

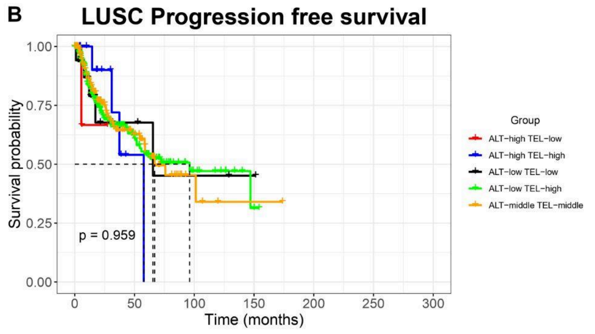

	ALT−high TEL−high	ALT−high TEL−low	ALT−low TEL−high	ALT−low TEL−low
ALT−high TEL−low	0.978	NA	NA	NA
ALT−low TEL−high	0.978	0.978	NA	NA
ALT−low TEL−low	0.978	0.978	0.978	NA
ALT−middle TEL−middle	0.978	0.978	0.978	0.978

Pairwise Log−Rank Test p−values

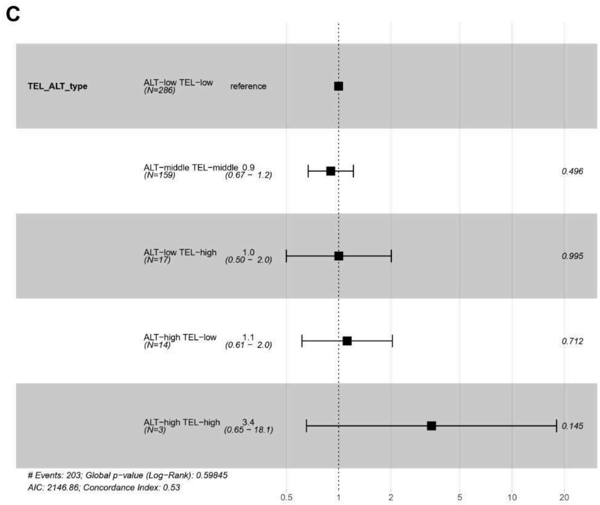

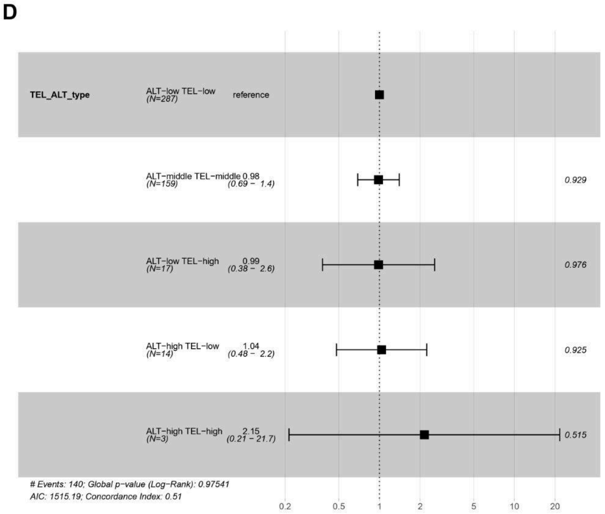

Figure S23. Survival and hazard ratio forest plots for LUSC ALT and TEL phenotypes (**A**) and (**C**) Overall survival. (**B**) and (**D**) Progression-free survival. Significance was calculated using a Log-rank test for K-M plots and a Cox proportional hazards regression model was used to estimate hazard ratios.The pairwise log-rank test was used to assess the significance between TMM phenotype groups.

A

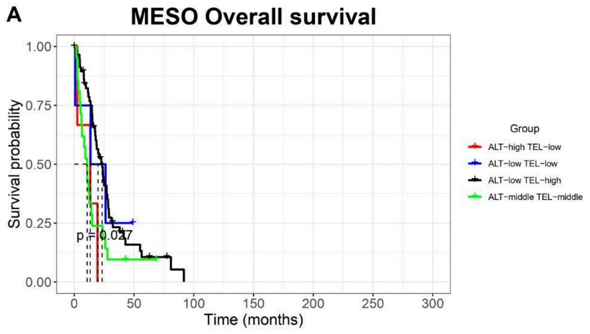

	ALT−high TEL−low	ALT−low TEL−high	ALT−low TEL−low
ALT−low TEL−high	0.5398	NA	NA
ALT−low TEL−low	0.0926	0.987	NA
ALT−middle TEL−middle	0.9664	0.5398	0.0568

Pairwise Log−Rank Test p−values

B

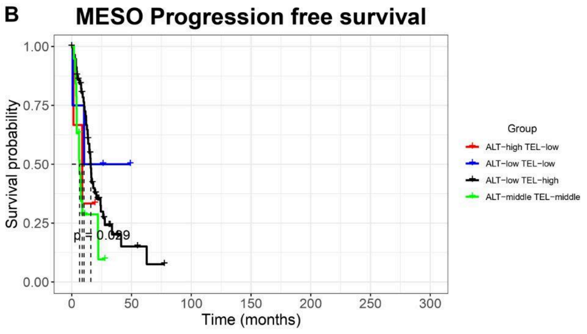

	ALT−high TEL−low	ALT−low TEL−high	ALT−low TEL−low
ALT−low TEL−high	0.793	NA	NA
ALT−low TEL−low	0.7735	0.7735	NA
ALT−middle TEL−middle	0.8664	0.5965	0.0215

Pairwise Log−Rank Test p−values

C

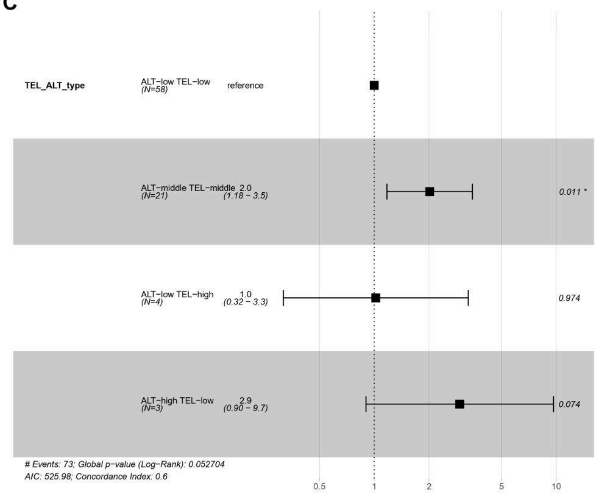

D

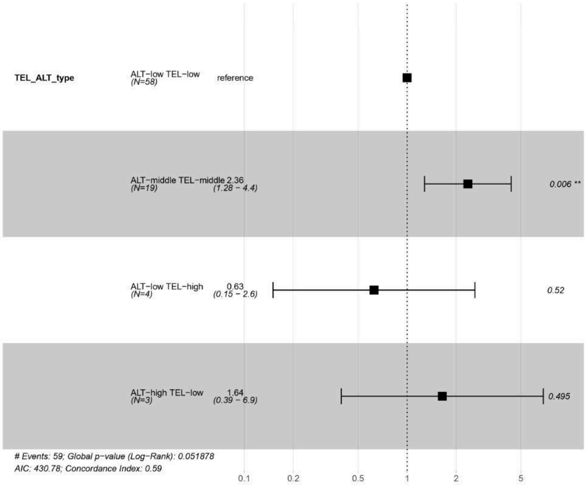

Figure S24. Survival and hazard ratio forest plots for MESO ALT and TEL phenotypes (**A**) and (**C**) Overall survival. (**B**) and (**D**) Progression-free survival. Significance was calculated using a Log-rank test for K-M plots and a Cox proportional hazards regression model was used to estimate hazard ratios.The pairwise log-rank test was used to assess the significance between TMM phenotype groups.

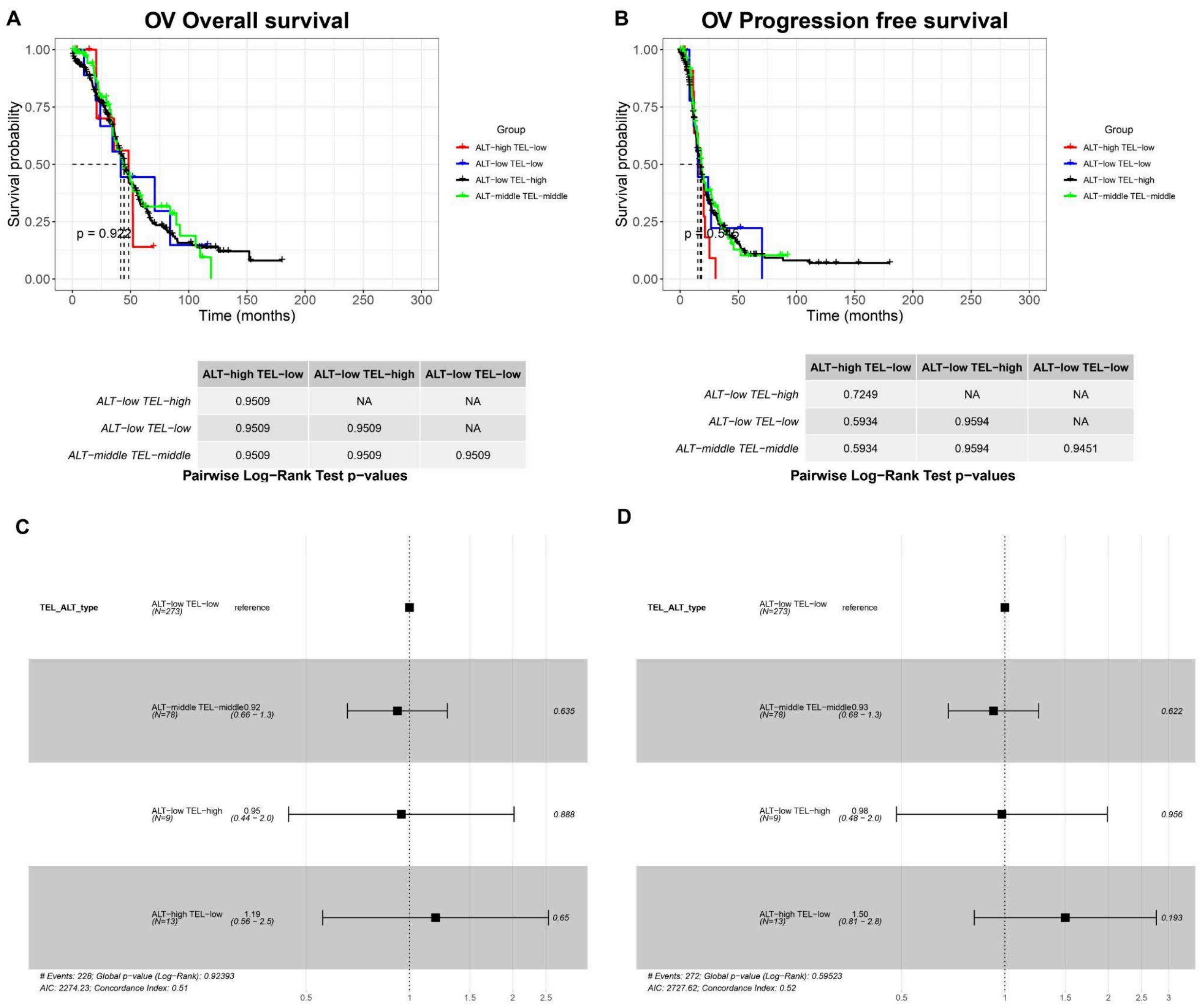

Figure S25. Survival and hazard ratio forest plots for OV ALT and TEL phenotypes (**A**) and (**C**) Overall survival. (**B**) and (**D**) Progression-free survival. Significance was calculated using a Log-rank test for K-M plots and a Cox proportional hazards regression model was used to estimate hazard ratios.The pairwise log-rank test was used to assess the significance between TMM phenotype groups.

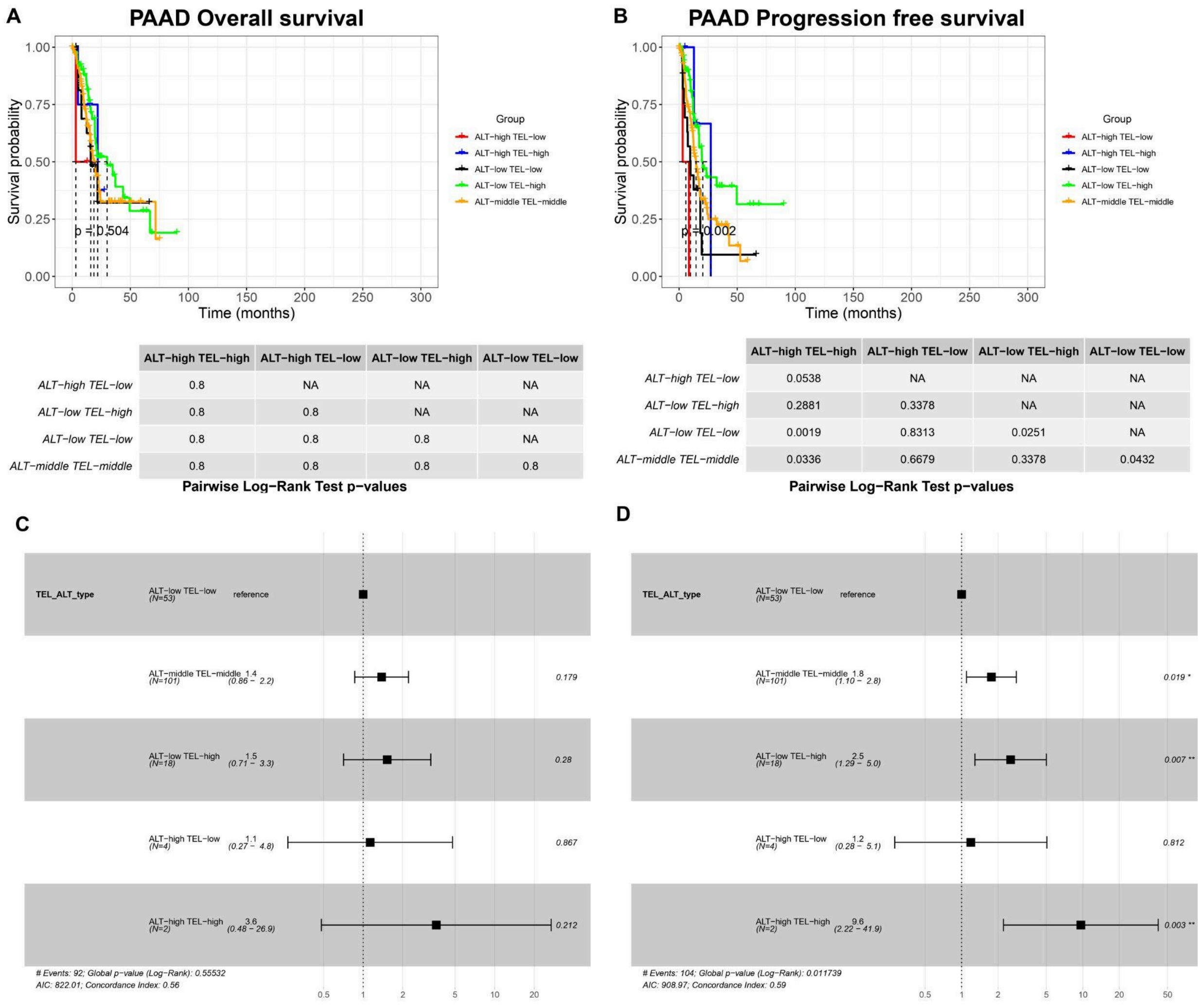

A – Pairwise Log−Rank Test p−values

	ALT−high TEL−high	ALT−high TEL−low	ALT−low TEL−high	ALT−low TEL−low
ALT−high TEL−low	0.8	NA	NA	NA
ALT−low TEL−high	0.8	0.8	NA	NA
ALT−low TEL−low	0.8	0.8	0.8	NA
ALT−middle TEL−middle	0.8	0.8	0.8	0.8

B – Pairwise Log−Rank Test p−values

	ALT−high TEL−high	ALT−high TEL−low	ALT−low TEL−high	ALT−low TEL−low
ALT−high TEL−low	0.0538	NA	NA	NA
ALT−low TEL−high	0.2881	0.3378	NA	NA
ALT−low TEL−low	0.0019	0.8313	0.0251	NA
ALT−middle TEL−middle	0.0336	0.6679	0.3378	0.0432

Figure S26. Survival and hazard ratio forest plots for PAAD ALT and TEL phenotypes (**A**) and (**C**) Overall survival. (**B**) and (**D**) Progression-free survival. Significance was calculated using a Log-rank test for K-M plots and a Cox proportional hazards regression model was used to estimate hazard ratios.The pairwise log-rank test was used to assess the significance between TMM phenotype groups.

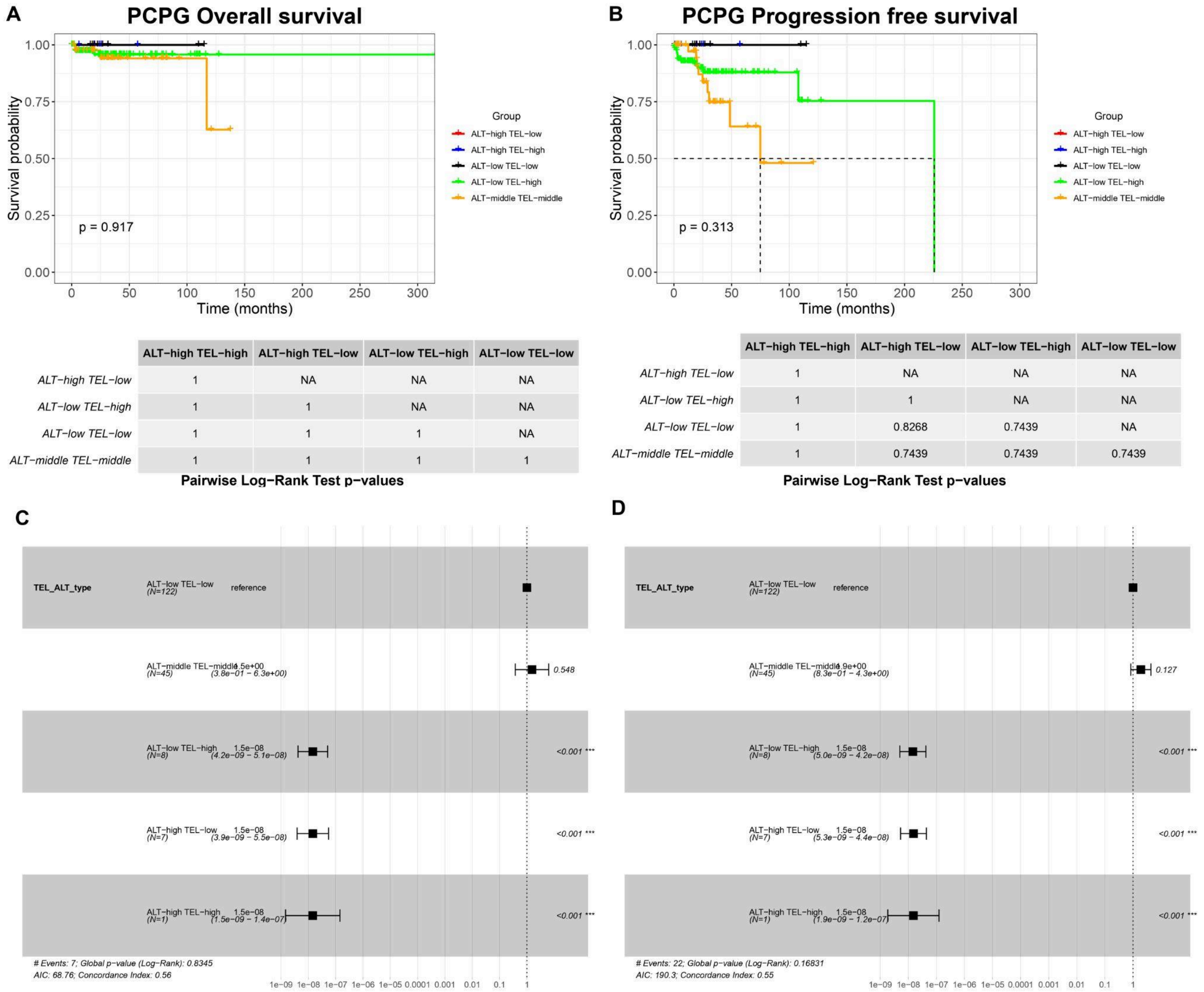

Figure S27. Survival and hazard ratio forest plots for PCPG ALT and TEL phenotypes (**A**) and (**C**) Overall survival. (**B**) and (**D**) Progression-free survival. Significance was calculated using a Log-rank test for K-M plots and a Cox proportional hazards regression model was used to estimate hazard ratios.The pairwise log-rank test was used to assess the significance between TMM phenotype groups.

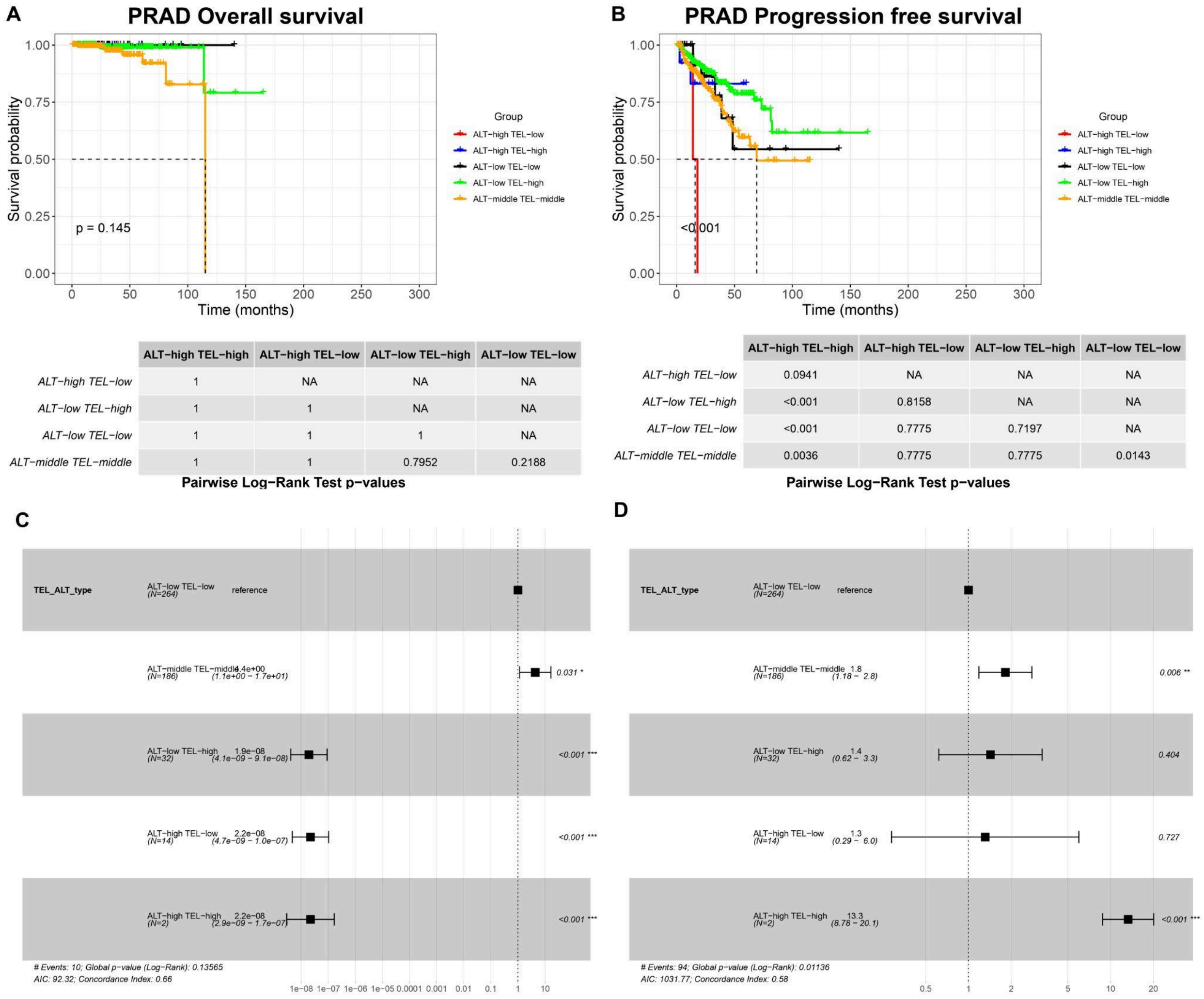

Figure S28. Survival and hazard ratio forest plots for PRAD ALT and TEL phenotypes (**A**) and (**C**) Overall survival. (**B**) and (**D**) Progression-free survival. Significance was calculated using a Log-rank test for K-M plots and a Cox proportional hazards regression model was used to estimate hazard ratios.The pairwise log-rank test was used to assess the significance between TMM phenotype groups.

A

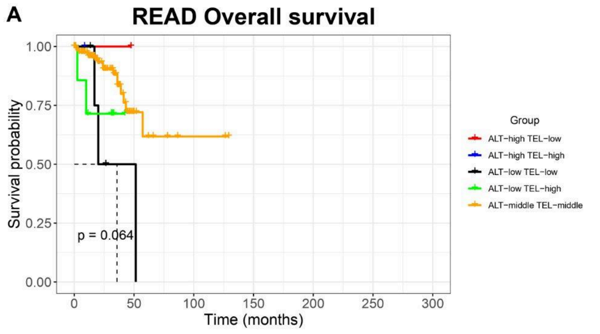

	ALT−high TEL−high	ALT−high TEL−low	ALT−low TEL−high	ALT−low TEL−low
ALT−high TEL−low	1	NA	NA	NA
ALT−low TEL−high	1	1	NA	NA
ALT−low TEL−low	1	1	1	NA
ALT−middle TEL−middle	1	1	0.0785	0.6751

Pairwise Log−Rank Test p−values

B

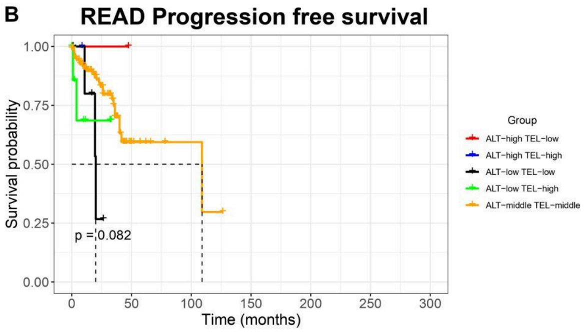

	ALT−high TEL−high	ALT−high TEL−low	ALT−low TEL−high	ALT−low TEL−low
ALT−high TEL−low	1	NA	NA	NA
ALT−low TEL−high	0.9305	1	NA	NA
ALT−low TEL−low	0.9305	0.9305	0.9789	NA
ALT−middle TEL−middle	0.9305	0.9789	0.1128	0.8528

Pairwise Log−Rank Test p−values

C

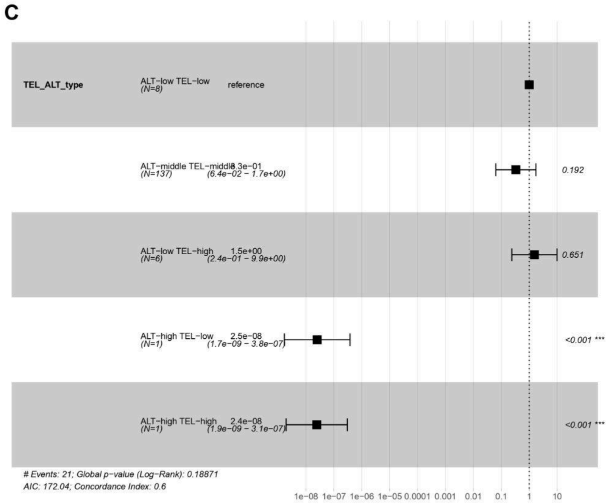

D

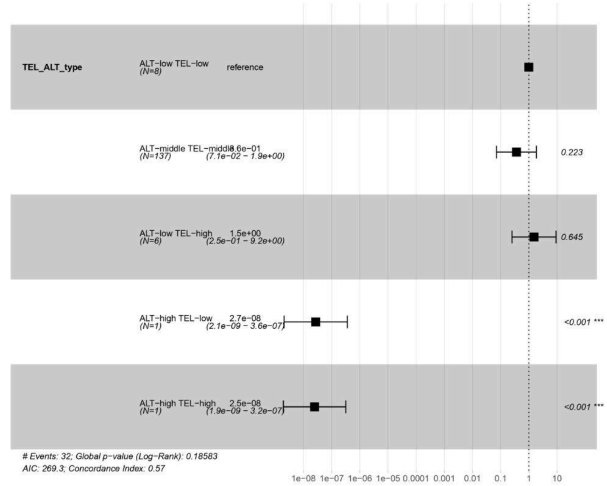

Figure S29. Survival and hazard ratio forest plots for READ ALT and TEL phenotypes (**A**) and (**C**) Overall survival. (**B**) and (**D**) Progression-free survival. Significance was calculated using a Log-rank test for K-M plots and a Cox proportional hazards regression model was used to estimate hazard ratios.The pairwise log-rank test was used to assess the significance between TMM phenotype groups.

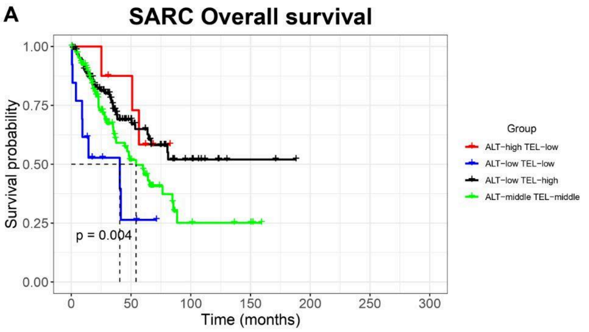

	ALT−high TEL−low	ALT−low TEL−high	ALT−low TEL−low
ALT−low TEL−high	0.0725	NA	NA
ALT−low TEL−low	0.7805	0.0118	NA
ALT−middle TEL−middle	0.3262	0.0719	0.0692

Pairwise Log−Rank Test p−values

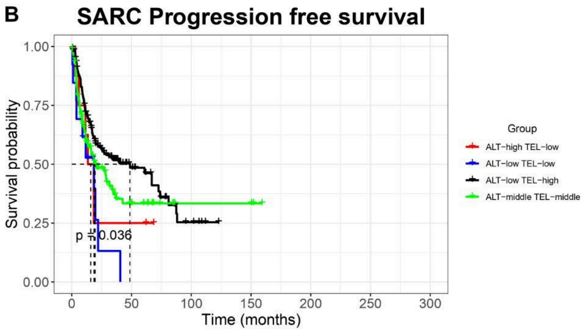

	ALT−high TEL−low	ALT−low TEL−high	ALT−low TEL−low
ALT−low TEL−high	0.61	NA	NA
ALT−low TEL−low	0.2892	0.0321	NA
ALT−middle TEL−middle	0.61	0.2183	0.2183

Pairwise Log−Rank Test p−values

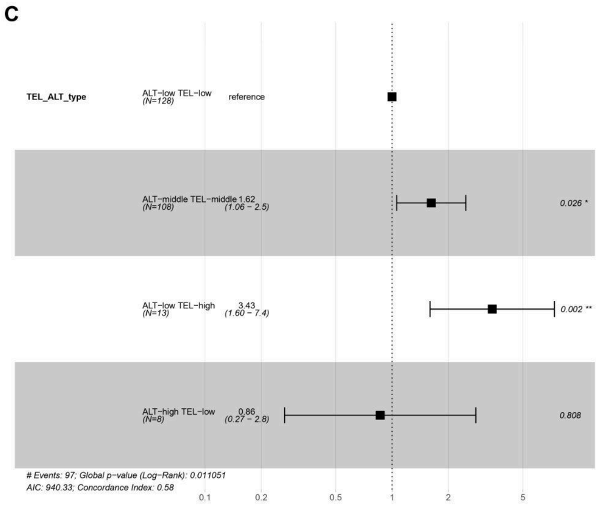

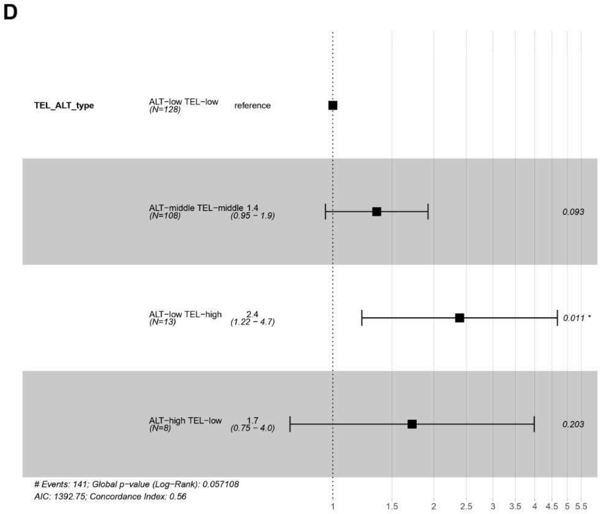

Figure S30. Survival and hazard ratio forest plots for SARC ALT and TEL phenotypes (**A**) and (**C**) Overall survival. (**B**) and (**D**) Progression-free survival. Significance was calculated using a Log-rank test for K-M plots and a Cox proportional hazards regression model was used to estimate hazard ratios.The pairwise log-rank test was used to assess the significance between TMM phenotype groups.

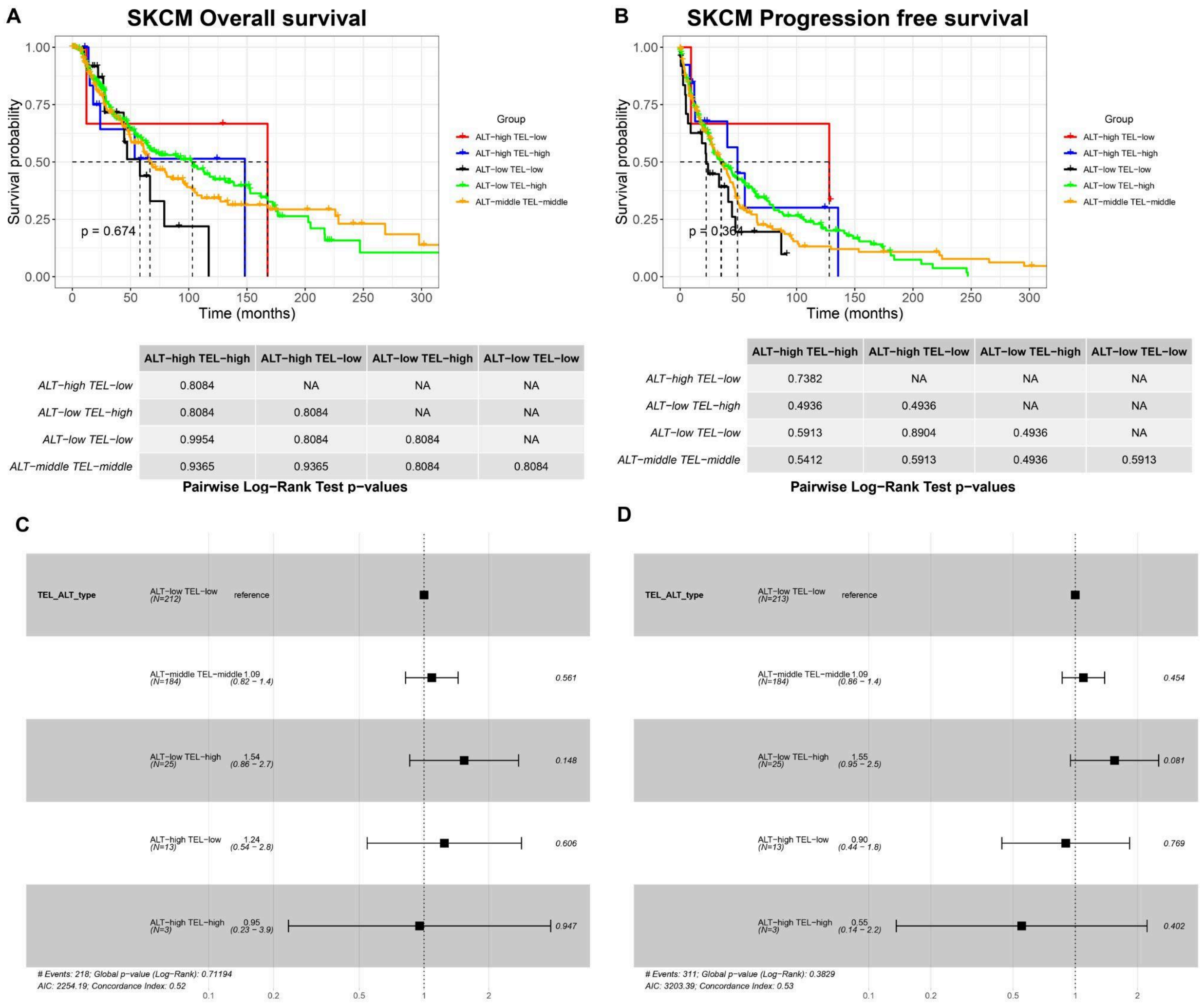

Figure S31. Survival and hazard ratio forest plots for SKCM ALT and TEL phenotypes (**A**) and (**C**) Overall survival. (**B**) and (**D**) Progression-free survival. Significance was calculated using a Log-rank test for K-M plots and a Cox proportional hazards regression model was used to estimate hazard ratios.The pairwise log-rank test was used to assess the significance between TMM phenotype groups.

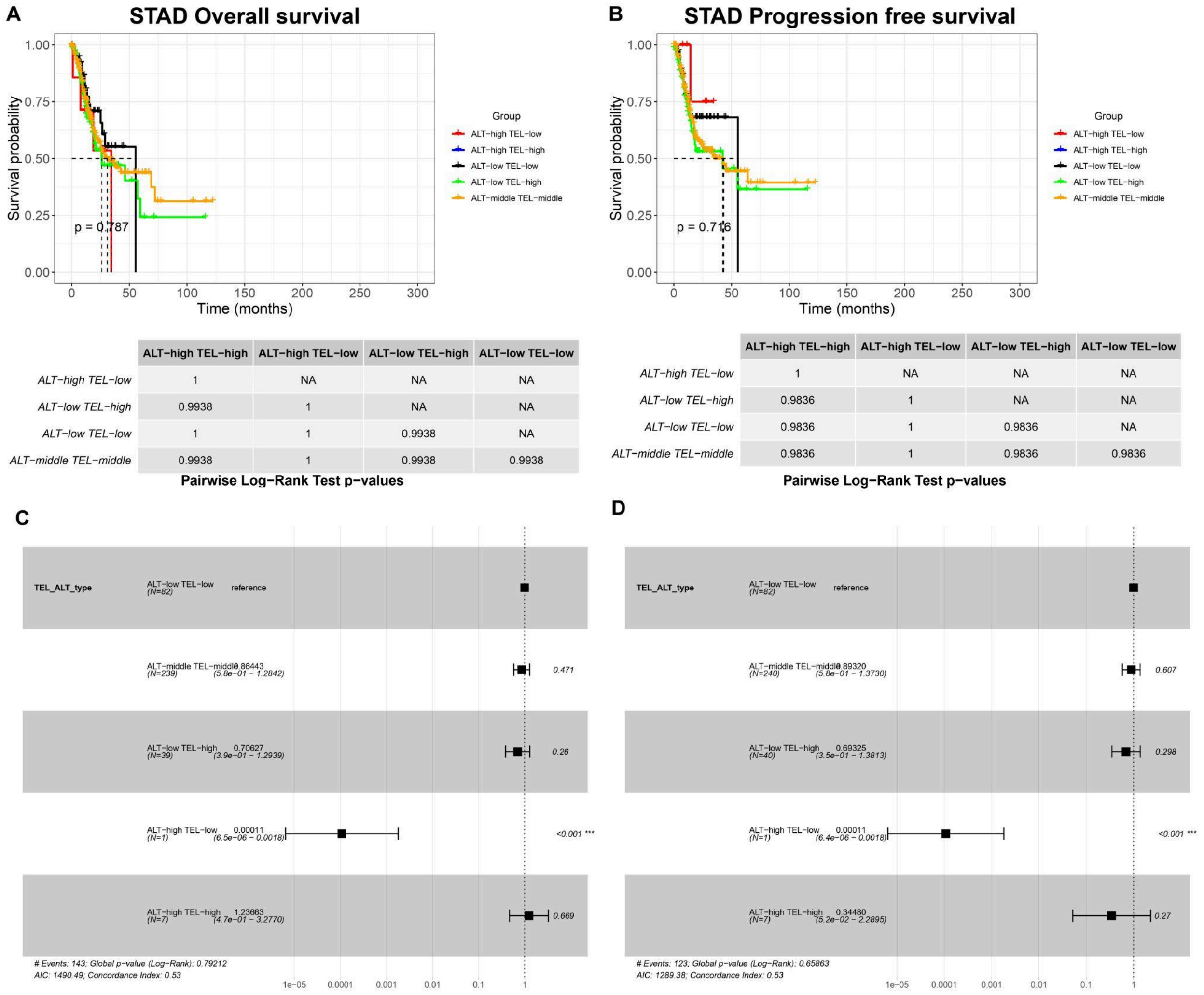

Figure S32. Survival and hazard ratio forest plots for STAD ALT and TEL phenotypes (**A**) and (**C**) Overall survival. (**B**) and (**D**) Progression-free survival. Significance was calculated using a Log-rank test for K-M plots and a Cox proportional hazards regression model was used to estimate hazard ratios.The pairwise log-rank test was used to assess the significance between TMM phenotype groups.

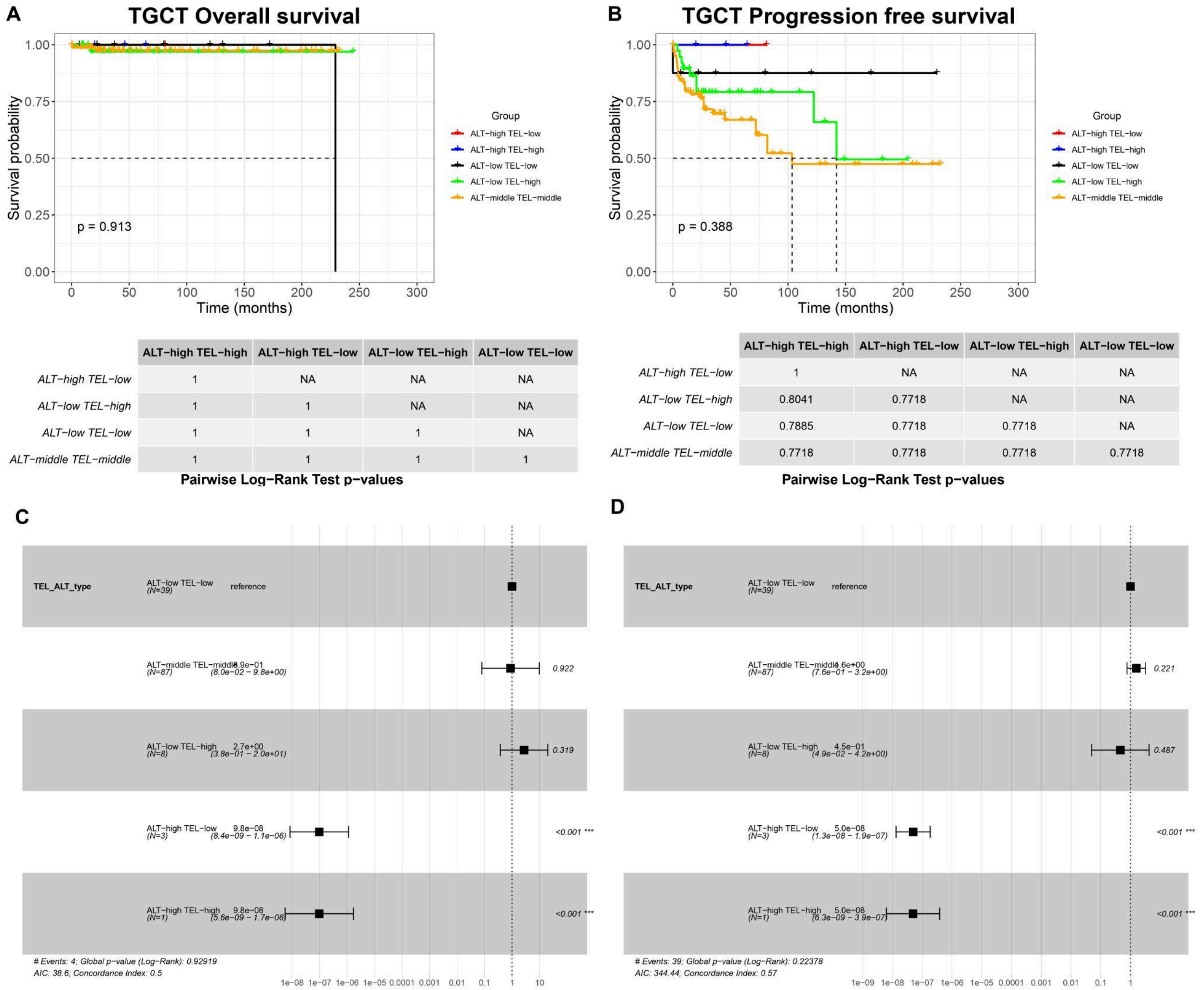

Figure S33. Survival and hazard ratio forest plots for TGCT ALT and TEL phenotypes (**A**) and (**C**) Overall survival. (**B**) and (**D**) Progression-free survival. Significance was calculated using a Log-rank test for K-M plots and a Cox proportional hazards regression model was used to estimate hazard ratios.The pairwise log-rank test was used to assess the significance between TMM phenotype groups.

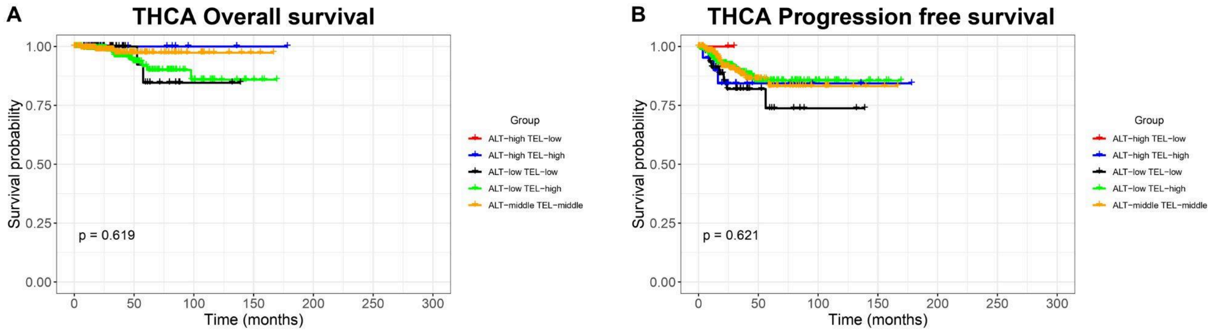

	ALT−high TEL−high	ALT−high TEL−low	ALT−low TEL−high	ALT−low TEL−low
ALT−high TEL−low	1	NA	NA	NA
ALT−low TEL−high	1	0.9933	NA	NA
ALT−low TEL−low	1	0.9933	1	NA
ALT−middle TEL−middle	1	1	0.9933	0.9933

Pairwise Log−Rank Test p−values

	ALT−high TEL−high	ALT−high TEL−low	ALT−low TEL−high	ALT−low TEL−low
ALT−high TEL−low	0.8477	NA	NA	NA
ALT−low TEL−high	0.8477	0.8561	NA	NA
ALT−low TEL−low	0.8477	0.8477	0.8477	NA
ALT−middle TEL−middle	0.8477	0.8477	0.8477	0.942

Pairwise Log−Rank Test p−values

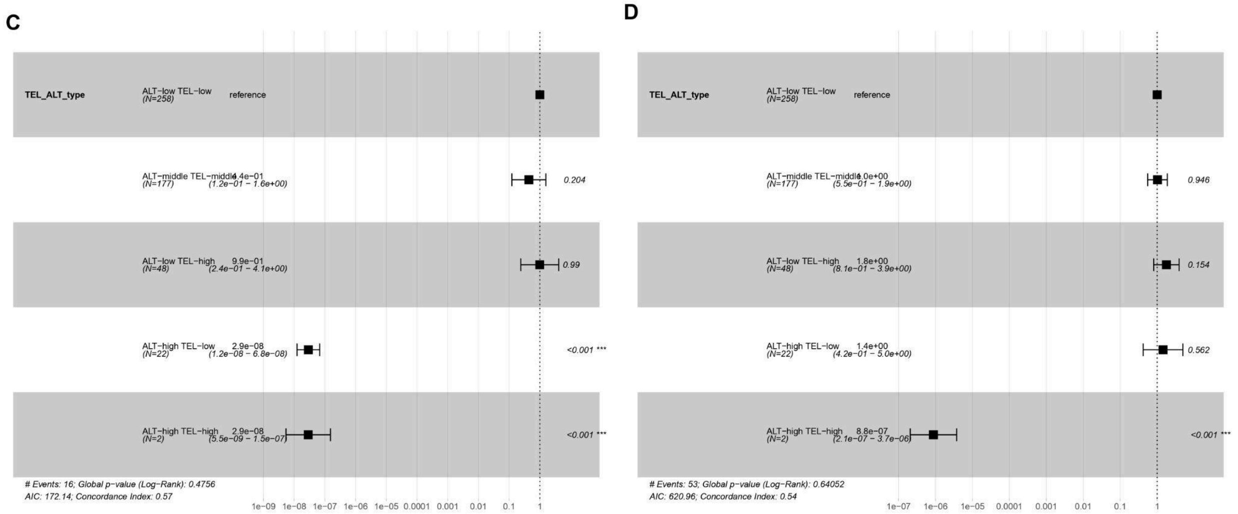

Figure S34. Survival and hazard ratio forest plots for THCA ALT and TEL phenotypes (**A**) and (**C**) Overall survival. (**B**) and (**D**) Progression-free survival. Significance was calculated using a Log-rank test for K-M plots and a Cox proportional hazards regression model was used to estimate hazard ratios.The pairwise log-rank test was used to assess the significance between TMM phenotype groups.

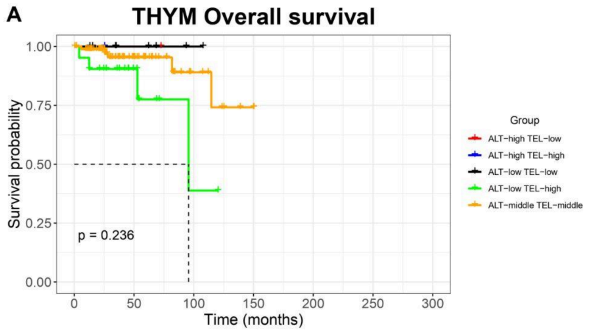

	ALT−high TEL−high	ALT−high TEL−low	ALT−low TEL−high	ALT−low TEL−low
ALT−high TEL−low	1	NA	NA	NA
ALT−low TEL−high	1	1	NA	NA
ALT−low TEL−low	1	1	0.8705	NA
ALT−middle TEL−middle	1	1	1	0.3801

Pairwise Log−Rank Test p−values

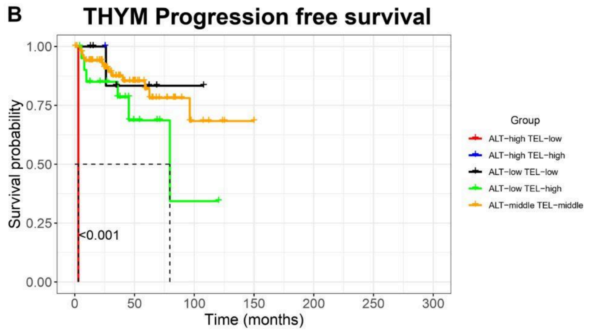

	ALT−high TEL−high	ALT−high TEL−low	ALT−low TEL−high	ALT−low TEL−low
ALT−high TEL−low	0.6346	NA	NA	NA
ALT−low TEL−high	0.0156	1.0000	NA	NA
ALT−low TEL−low	<0.001	0.8898	0.6790	NA
ALT−middle TEL−middle	<0.001	0.8898	0.8898	0.3239

Pairwise Log−Rank Test p−values

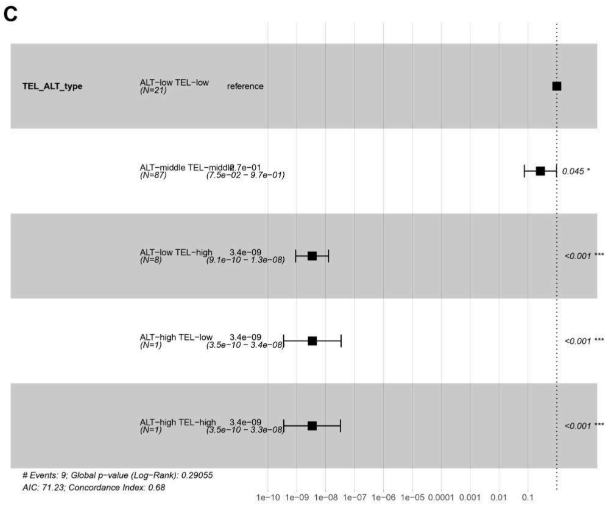

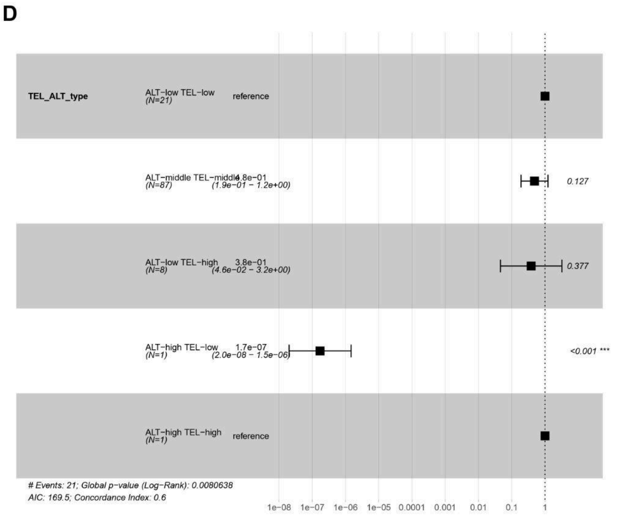

Figure S35. Survival and hazard ratio forest plots for THYM ALT and TEL phenotypes (**A**) and (**C**) Overall survival. (**B**) and (**D**) Progression-free survival. Significance was calculated using a Log-rank test for K-M plots and a Cox proportional hazards regression model was used to estimate hazard ratios.The pairwise log-rank test was used to assess the significance between TMM phenotype groups.

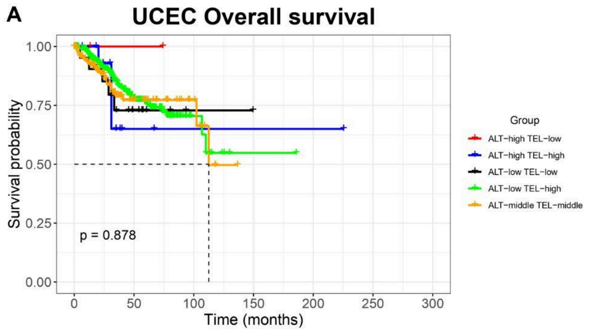

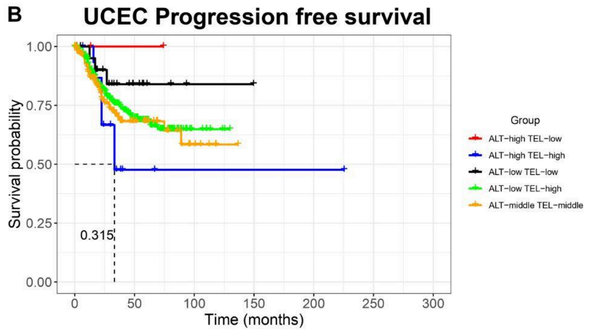

	ALT−high TEL−high	ALT−high TEL−low	ALT−low TEL−high	ALT−low TEL−low
ALT−high TEL−low	0.8841	NA	NA	NA
ALT−low TEL−high	0.8841	0.8841	NA	NA
ALT−low TEL−low	0.8841	0.8841	0.8841	NA
ALT−middle TEL−middle	0.8841	0.8841	0.8841	0.8841

Pairwise Log−Rank Test p−values

	ALT−high TEL−high	ALT−high TEL−low	ALT−low TEL−high	ALT−low TEL−low
ALT−high TEL−low	0.5482	NA	NA	NA
ALT−low TEL−high	0.6396	0.5482	NA	NA
ALT−low TEL−low	0.5482	0.5482	0.5482	NA
ALT−middle TEL−middle	0.5482	0.5482	0.5482	0.5482

Pairwise Log−Rank Test p−values

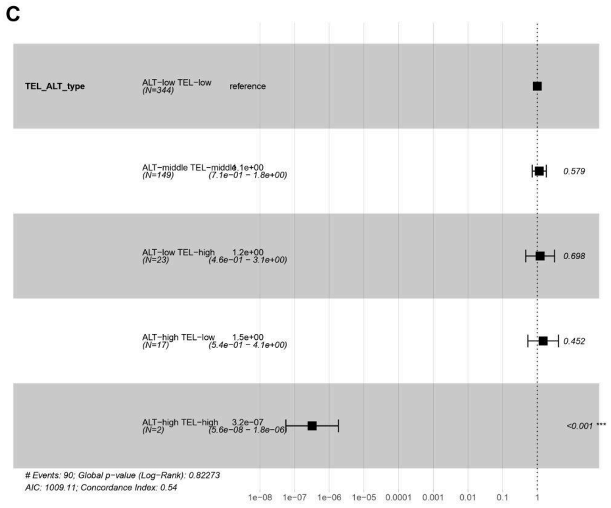

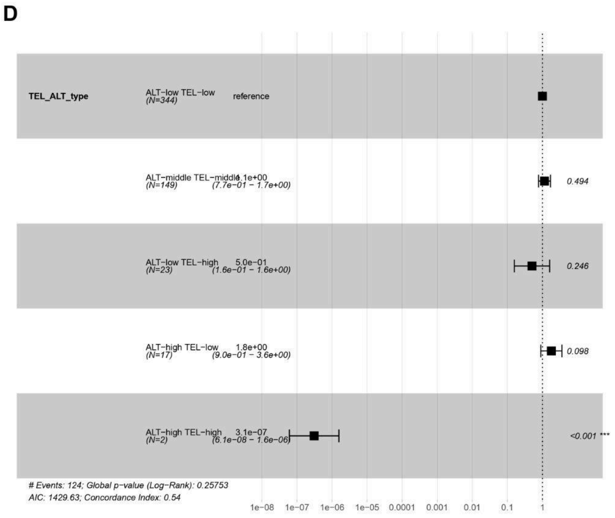

Figure S36. Survival and hazard ratio forest plots for UCEC ALT and TEL phenotypes (**A**) and (**C**) Overall survival. (**B**) and (**D**) Progression-free survival. Significance was calculated using a Log-rank test for K-M plots and a Cox proportional hazards regression model was used to estimate hazard ratios.The pairwise log-rank test was used to assess the significance between TMM phenotype groups.

A

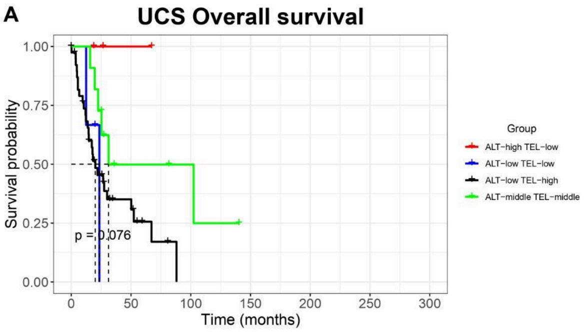

	ALT−high TEL−low	ALT−low TEL−high	ALT−low TEL−low
ALT−low TEL−high	0.1695	NA	NA
ALT−low TEL−low	0.1695	0.8873	NA
ALT−middle TEL−middle	0.3329	0.1695	0.1695

Pairwise Log−Rank Test p−values

B

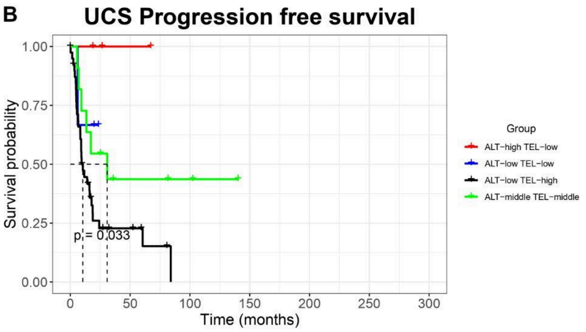

	ALT−high TEL−low	ALT−low TEL−high	ALT−low TEL−low
ALT−low TEL−high	0.3808	NA	NA
ALT−low TEL−low	0.1478	0.3808	NA
ALT−middle TEL−middle	0.3352	0.8263	0.1478

Pairwise Log−Rank Test p−values

C

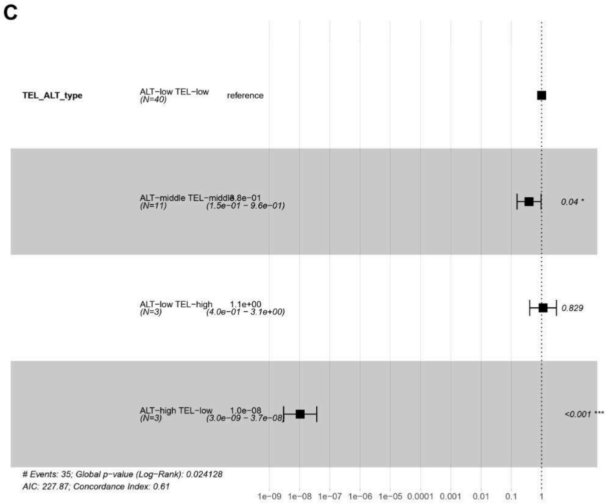

D

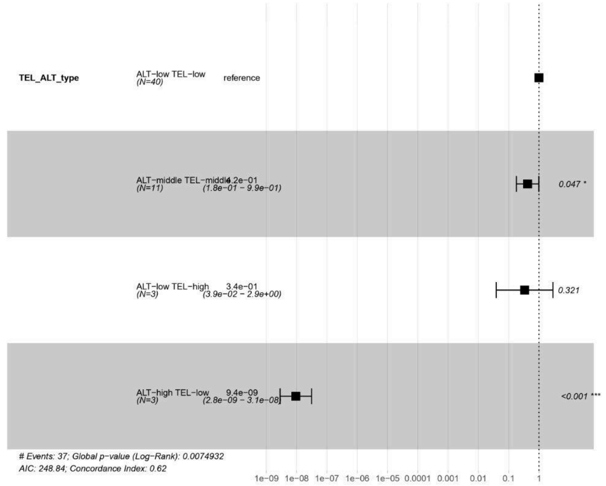

Figure S37. Survival and hazard ratio forest plots for UCS ALT and TEL phenotypes (**A**) and (**C**) Overall survival. (**B**) and (**D**) Progression-free survival. Significance was calculated using a Log-rank test for K-M plots and a Cox proportional hazards regression model was used to estimate hazard ratios.The pairwise log-rank test was used to assess the significance between TMM phenotype groups.

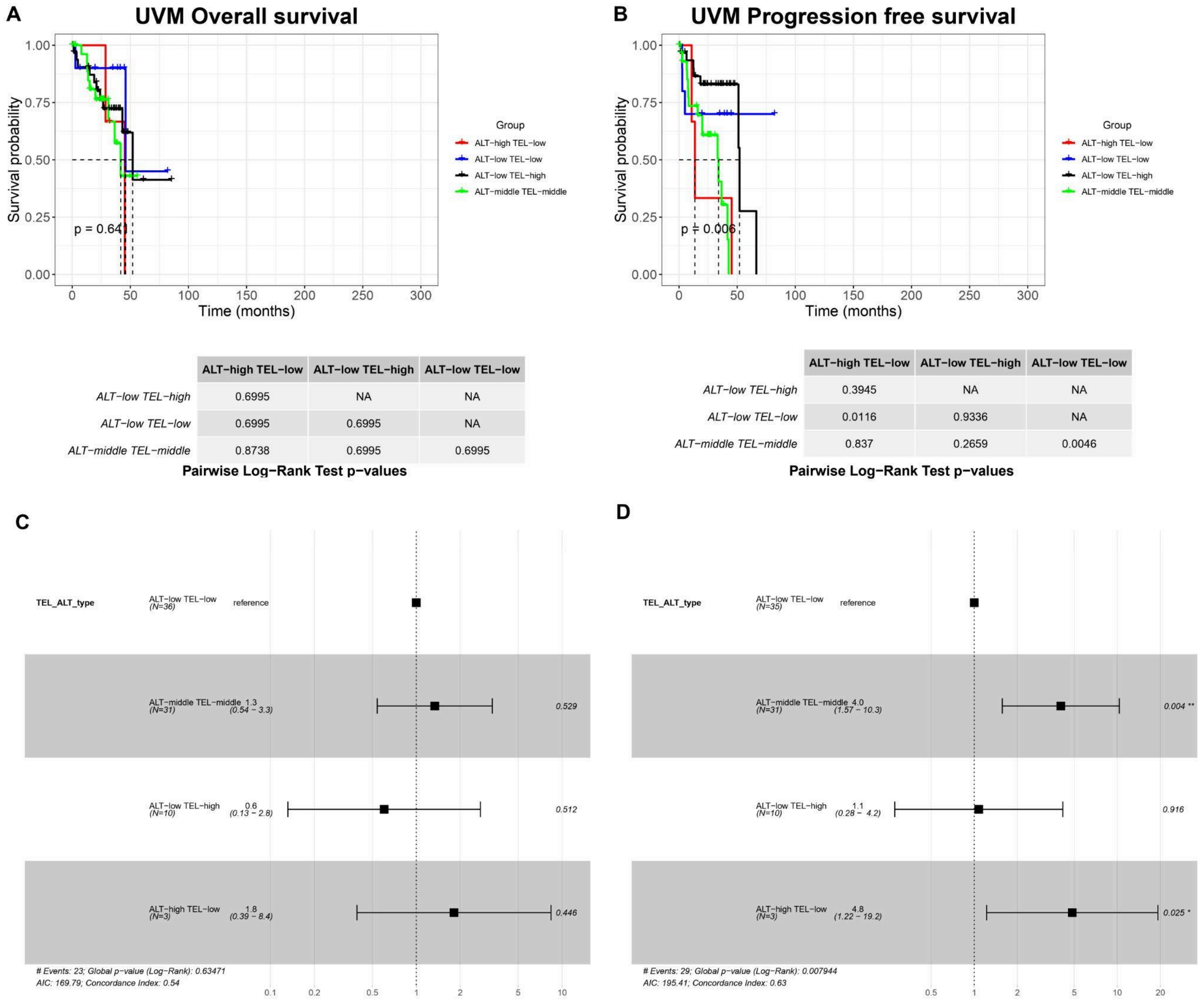

Figure S38. Survival and hazard ratio forest plots for UVM ALT and TEL phenotypes (**A**) and (**C**) Overall survival. (**B**) and (**D**) Progression-free survival. Significance was calculated using a Log-rank test for K-M plots and a Cox proportional hazards regression model was used to estimate hazard ratios.The pairwise log-rank test was used to assess the significance between TMM phenotype groups.

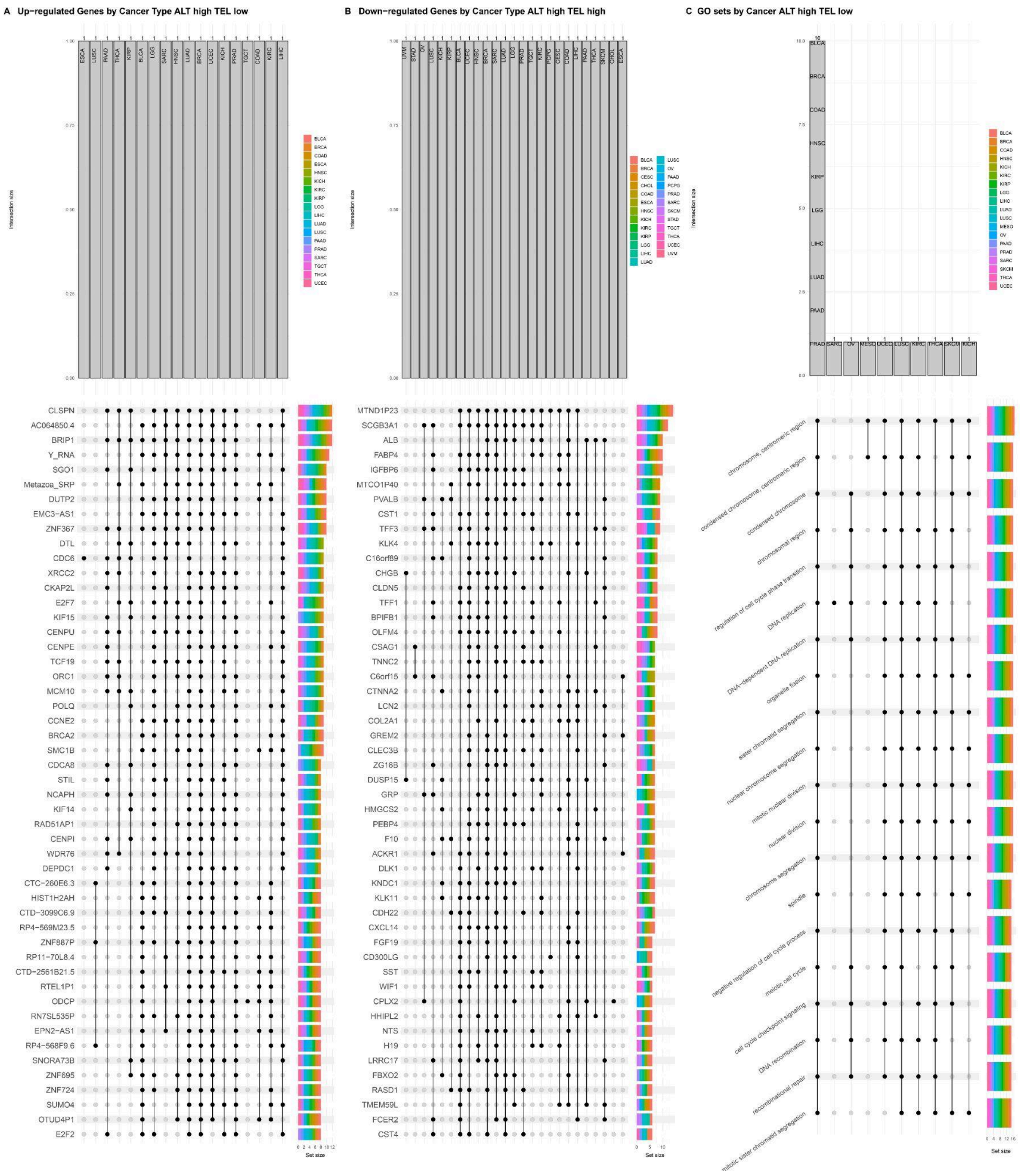

Figure S39. Up-regulated and down-regulated genes and over-represented GO terms UpSet plot for the pan-cancer studies. (**A**) Frequently up-regulated genes by cancer types for ALT ^{high} TEL ^{low} phenotype, color coding corresponds to the cancer type. The top 50 were chosen. (**B**) Frequently down-regulated genes by cancer types for ALT ^{high} TEL ^{low} phenotype, color coding corresponds to the cancer type. The top 50 were chosen. (**C**) Over-represented GO terms by cancer types for ALT ^{high} TEL ^{low} phenotype, color coding corresponds to the cancer type. The top 20 were chosen. Matched normal samples have been removed.

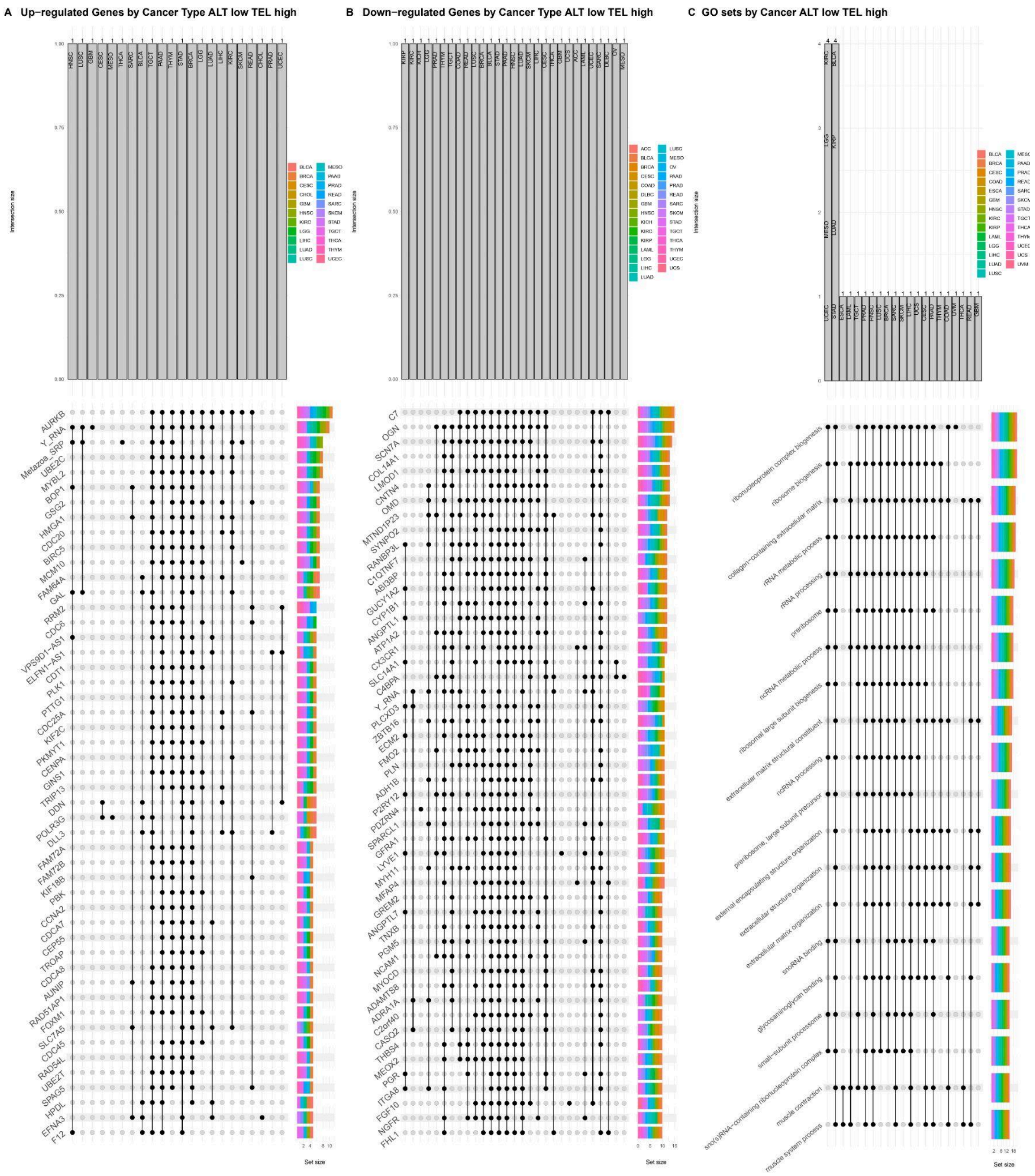

Figure S40. Up-regulated and down-regulated genes and over-represented GO terms UpSet plot for the pan-cancer studies. (**A**) Frequently up-regulated genes by cancer types for ALT ^{low} TEL ^{high} phenotype, color coding corresponds to the cancer type. The top 50 were chosen. (**B**) Frequently down-regulated genes by cancer types for ALT ^{low} TEL ^{high} phenotype, color coding corresponds to the cancer type. The top 50 were chosen. (**C**) Over-represented GO terms by cancer types for ALT ^{low} TEL ^{high} phenotype, color coding corresponds to the cancer type. The top 20 were chosen. Matched normal samples have been removed.

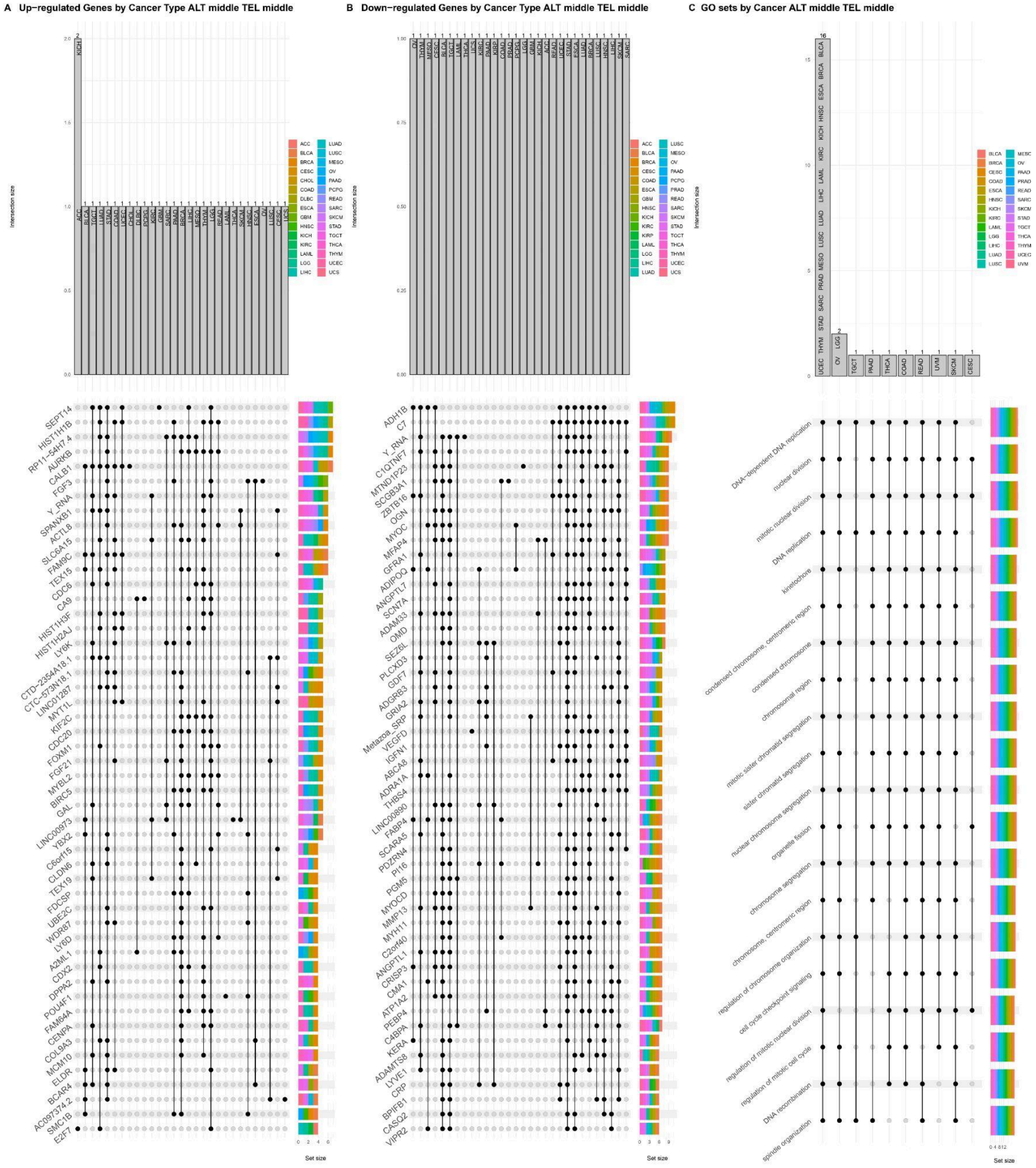

Figure S41. Up-regulated and down-regulated genes and over-represented GO terms UpSet plot for the pan-cancer studies. (**A**) Frequently up-regulated genes by cancer types for ALT ^{middle} TEL ^{middle} phenotype, color coding corresponds to the cancer type. The top 50 were chosen. (**B**) Frequently down-regulated genes by cancer types for ALT ^{middle} TEL ^{middle} phenotype, color coding corresponds to the cancer type. The top 50 were chosen. (**C**) Over-represented GO terms by cancer types for ALT ^{middle} TEL ^{middle} phenotype, color coding corresponds to the cancer type. The top 20 were chosen. Matched normal samples have been removed.

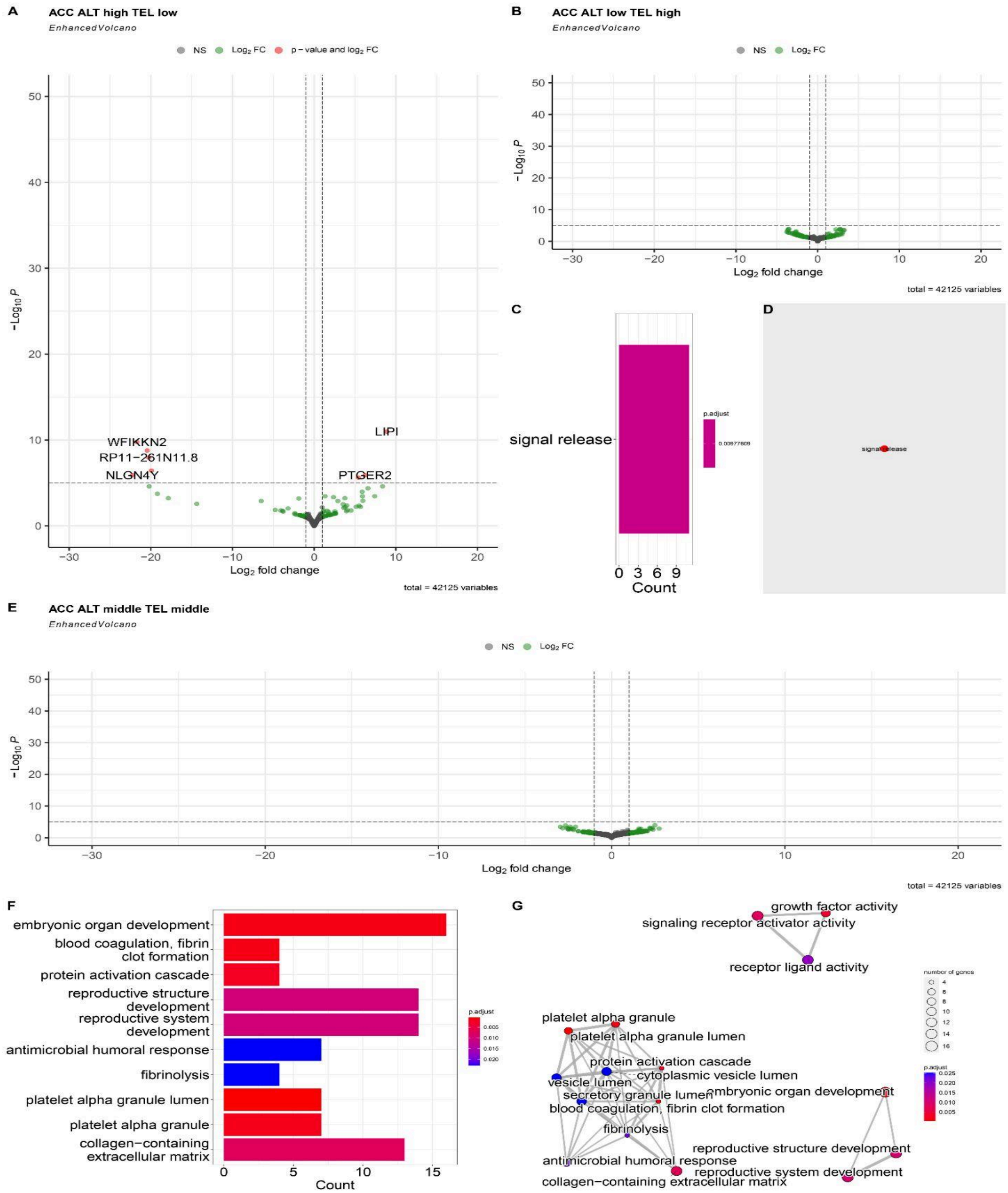

Figure S42. ACC DEG results and GO over-represented terms. (**A**) Volcano plot of differentially expressed genes (DEGs) for ALT ^{high} TEL ^{low} phenotype. Over-represented terms were missing. (**B**) Volcano plot of differentially expressed genes (DEGs) for ALT ^{low} TEL ^{high} phenotype. (**C**) Barplot displays the top GO terms by Adjusted p-value. Only one term "signal release" was over-represented. (**D**) Enrichmap clusters the most significant (by padj) GO terms to visualize relationships between terms. Only one term "signal release" was over-represented. (**E**) Volcano plot of differentially expressed genes (DEGs) for ALT ^{middle} TEL ^{middle} phenotype. (**F**) Barplot displays the top GO terms by Adjusted p-value. (**G**) Enrichmap clusters the most significant (by padj) GO terms to visualize relationships between terms. Matched normal samples have been removed.

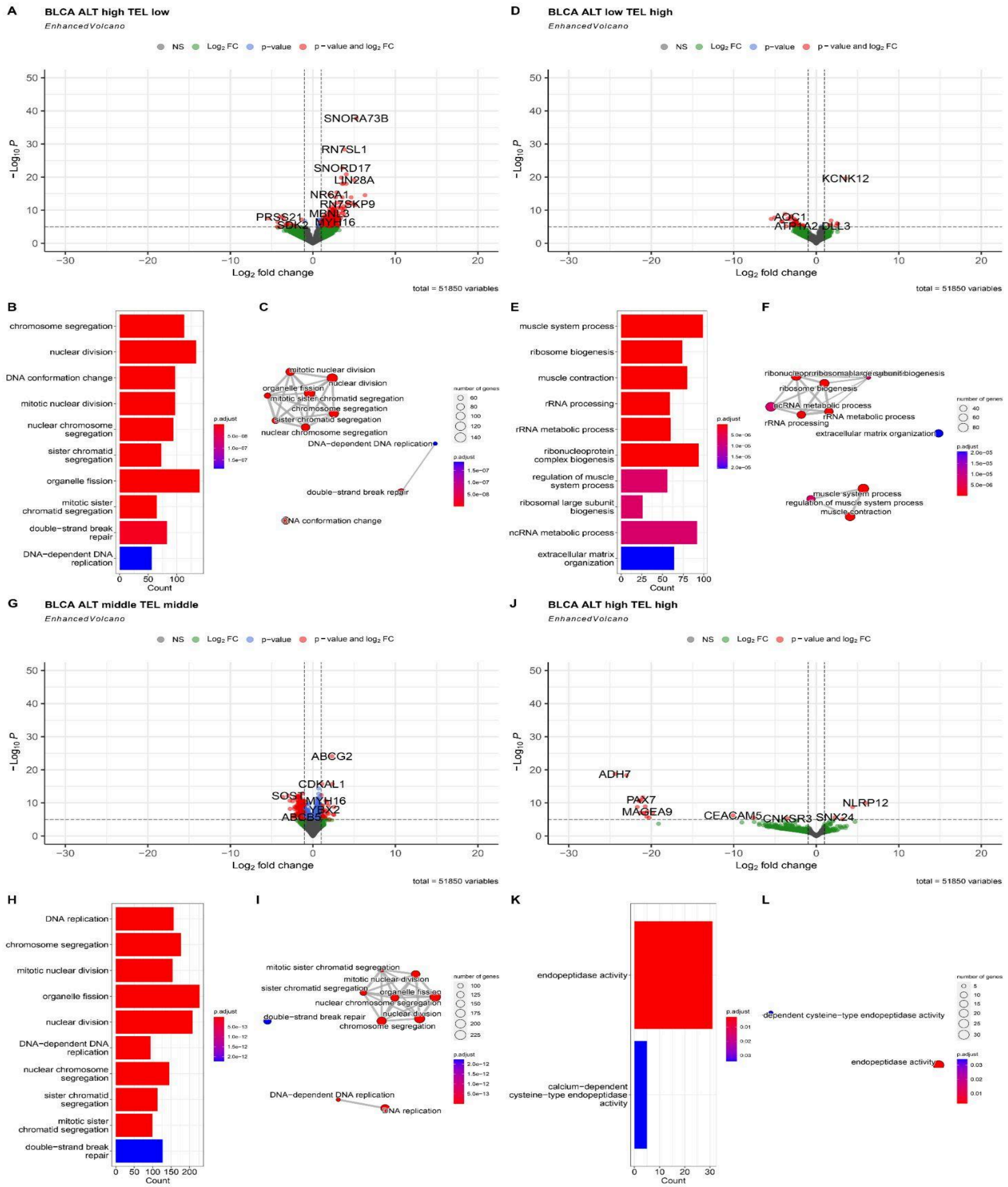

Figure S43. BLCA DEG results and GO over-represented terms. (**A**) Volcano plot of differentially expressed genes (DEGs) for ALT ^{high} TEL ^{low} phenotype. (**B**) Barplot displays the top GO terms by Adjusted p-value. (**C**) Enrichmap clusters the most significant (by padj) GO terms to visualize relationships between terms. (**D**) Volcano plot of differentially expressed genes (DEGs) for ALT ^{low} TEL ^{high} phenotype. (**E**) Barplot displays the top GO terms by Adjusted p-value. (**F**) Enrichmap clusters the most significant (by padj) GO terms to visualize relationships between terms. (**G**) Volcano plot of differentially expressed genes (DEGs) for ALT ^{middle} TEL ^{middle} phenotype. (**H**) Barplot displays the top GO terms by Adjusted p-value. (**I**) Enrichmap clusters the most significant (by padj) GO terms to visualize relationships between terms. (**J**) Volcano plot of differentially expressed genes (DEGs) for ALT ^{high} TEL^{high} phenotype. (**K**) Barplot displays the top GO terms by Adjusted p-value. (**L**) Enrichmap clusters the most significant (by padj) GO terms to visualize relationships between terms. Matched normal samples have been removed.

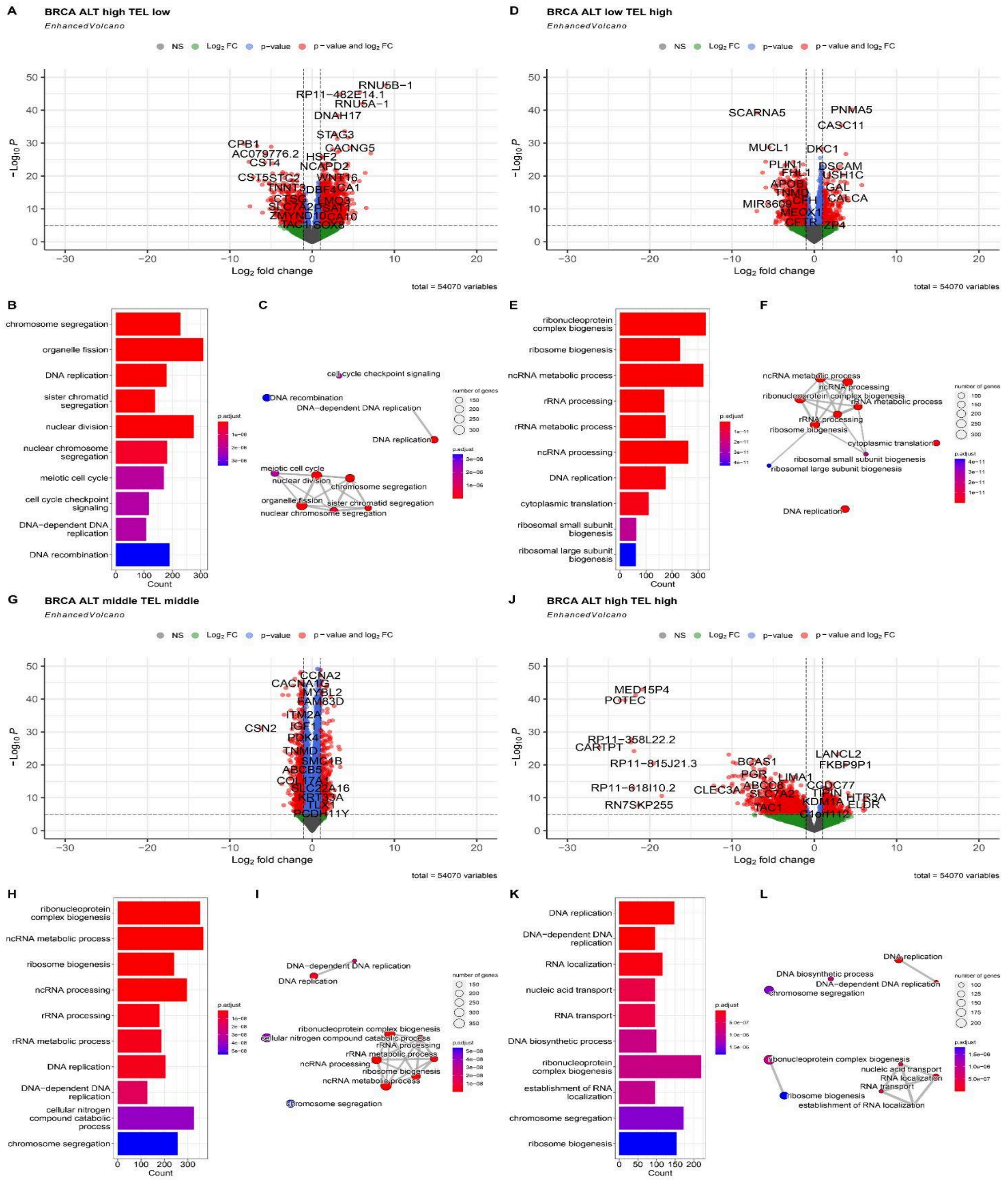

Figure S44. BRCA DEG results and GO over-represented terms. (**A**) Volcano plot of differentially expressed genes (DEGs) for ALT ^{high} TEL ^{low} phenotype. (**B**) Barplot displays the top GO terms by Adjusted p-value. (**C**) Enrichmap clusters the most significant (by padj) GO terms to visualize relationships between terms. (**D**) Volcano plot of differentially expressed genes (DEGs) for ALT ^{low} TEL ^{high} phenotype. (**E**) Barplot displays the top GO terms by Adjusted p-value. (**F**) Enrichmap clusters the most significant (by padj) GO terms to visualize relationships between terms. (**G**) Volcano plot of differentially expressed genes (DEGs) for ALT ^{middle} TEL ^{middle} phenotype. (**H**) Barplot displays the top GO terms by Adjusted p-value. (**I**) Enrichmap clusters the most significant (by padj) GO terms to visualize relationships between terms. (**J**) Volcano plot of differentially expressed genes (DEGs) for ALT ^{high} TEL^{high} phenotype. (**K**) Barplot displays the top GO terms by Adjusted p-value. (**L**) Enrichmap clusters the most significant (by padj) GO terms to visualize relationships between terms. Matched normal samples have been removed.

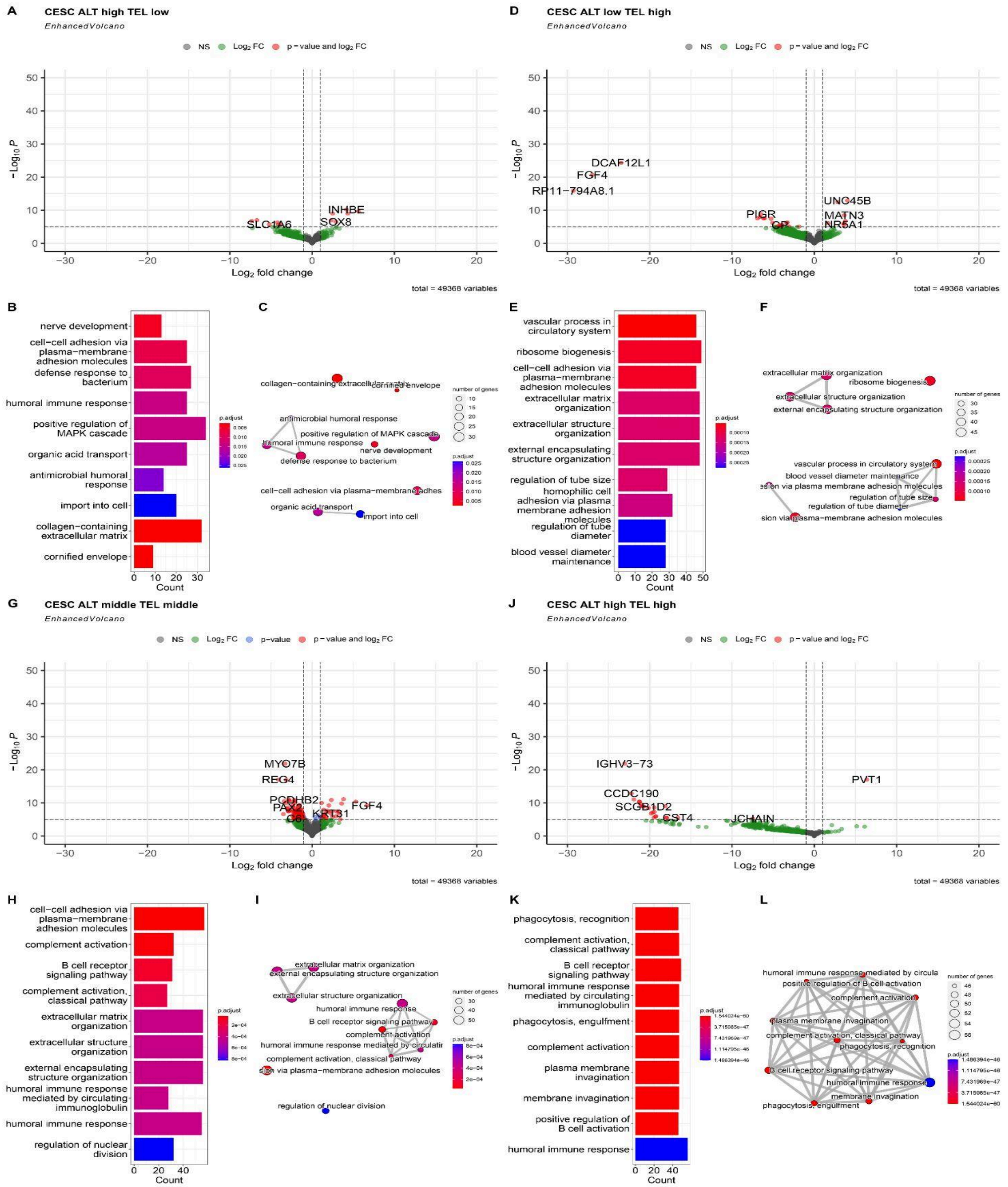

Figure S45. CESC DEG results and GO over-represented terms. (**A**) Volcano plot of differentially expressed genes (DEGs) for ALT ^{high} TEL ^{low} phenotype. (**B**) Barplot displays the top GO terms by Adjusted p-value. (**C**) Enrichmap clusters the most significant (by padj) GO terms to visualize relationships between terms. (**D**) Volcano plot of differentially expressed genes (DEGs) for ALT ^{low} TEL ^{high} phenotype. (**E**) Barplot displays the top GO terms by Adjusted p-value. (**F**) Enrichmap clusters the most significant (by padj) GO terms to visualize relationships between terms. (**G**) Volcano plot of differentially expressed genes (DEGs) for ALT ^{middle} TEL ^{middle} phenotype. (**H**) Barplot displays the top GO terms by Adjusted p-value. (**I**) Enrichmap clusters the most significant (by padj) GO terms to visualize relationships between terms. (**J**) Volcano plot of differentially expressed genes (DEGs) for ALT ^{high} TEL^{high} phenotype. (**K**) Barplot displays the top GO terms by Adjusted p-value. (**L**) Enrichmap clusters the most significant (by padj) GO terms to visualize relationships between terms. Matched normal samples have been removed.

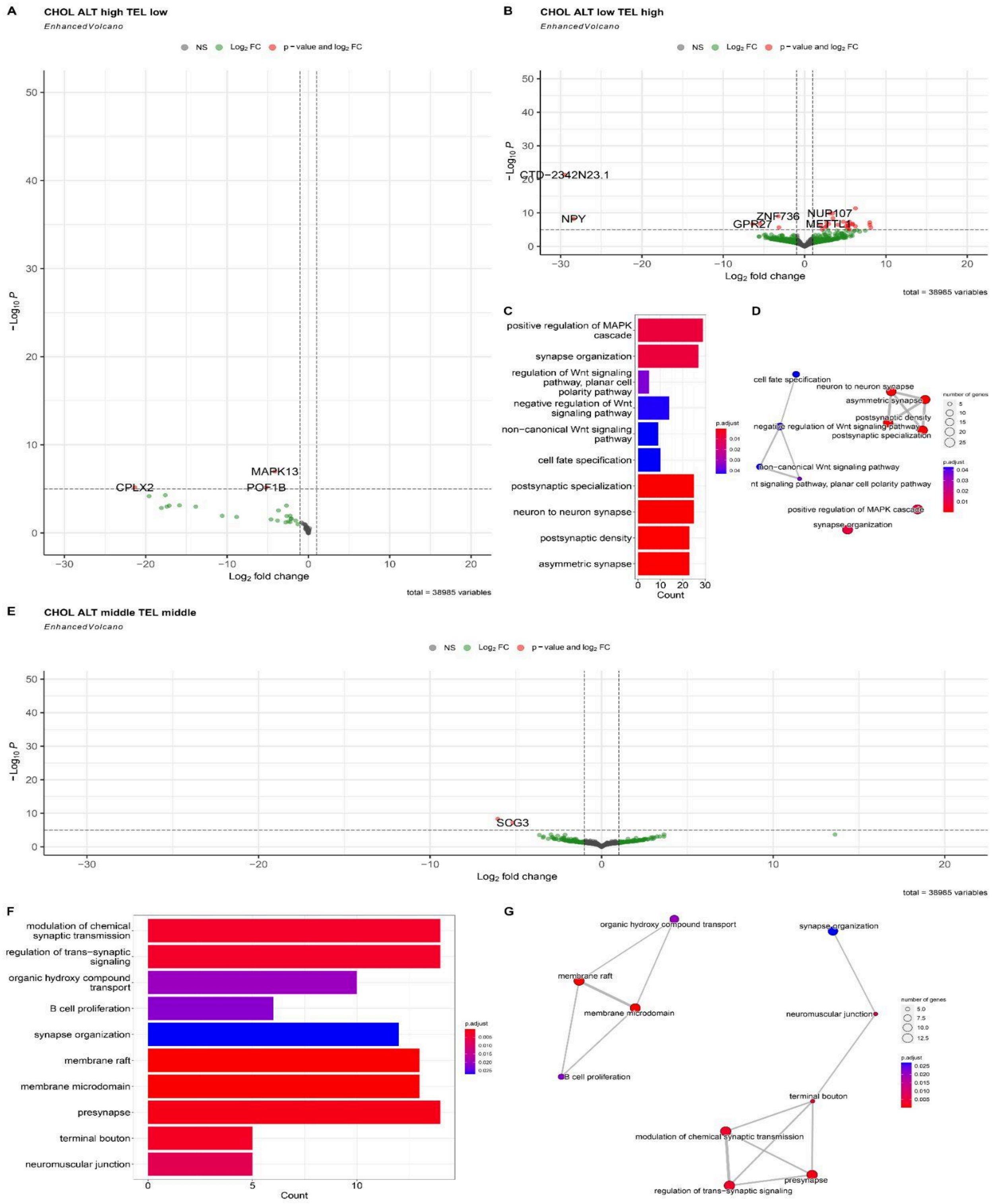

Figure S46. CHOL DEG results and GO over-represented terms. (**A**) Volcano plot of differentially expressed genes (DEGs) for ALT ^{high} TEL ^{low} phenotype. Over-represented terms were missing. (**B**) Volcano plot of differentially expressed genes (DEGs) for ALT ^{low} TEL ^{high} phenotype. (**C**) Barplot displays the top GO terms by Adjusted p-value. (**D**) Enrichmap clusters the most significant (by padj) GO terms to visualize relationships between terms. (**E**) Volcano plot of differentially expressed genes (DEGs) for ALT ^{middle} TEL ^{middle} phenotype. (**F**) Barplot displays the top GO terms by Adjusted p-value. (**G**) Enrichmap clusters the most significant (by padj) GO terms to visualize relationships between terms. Matched normal samples have been removed.

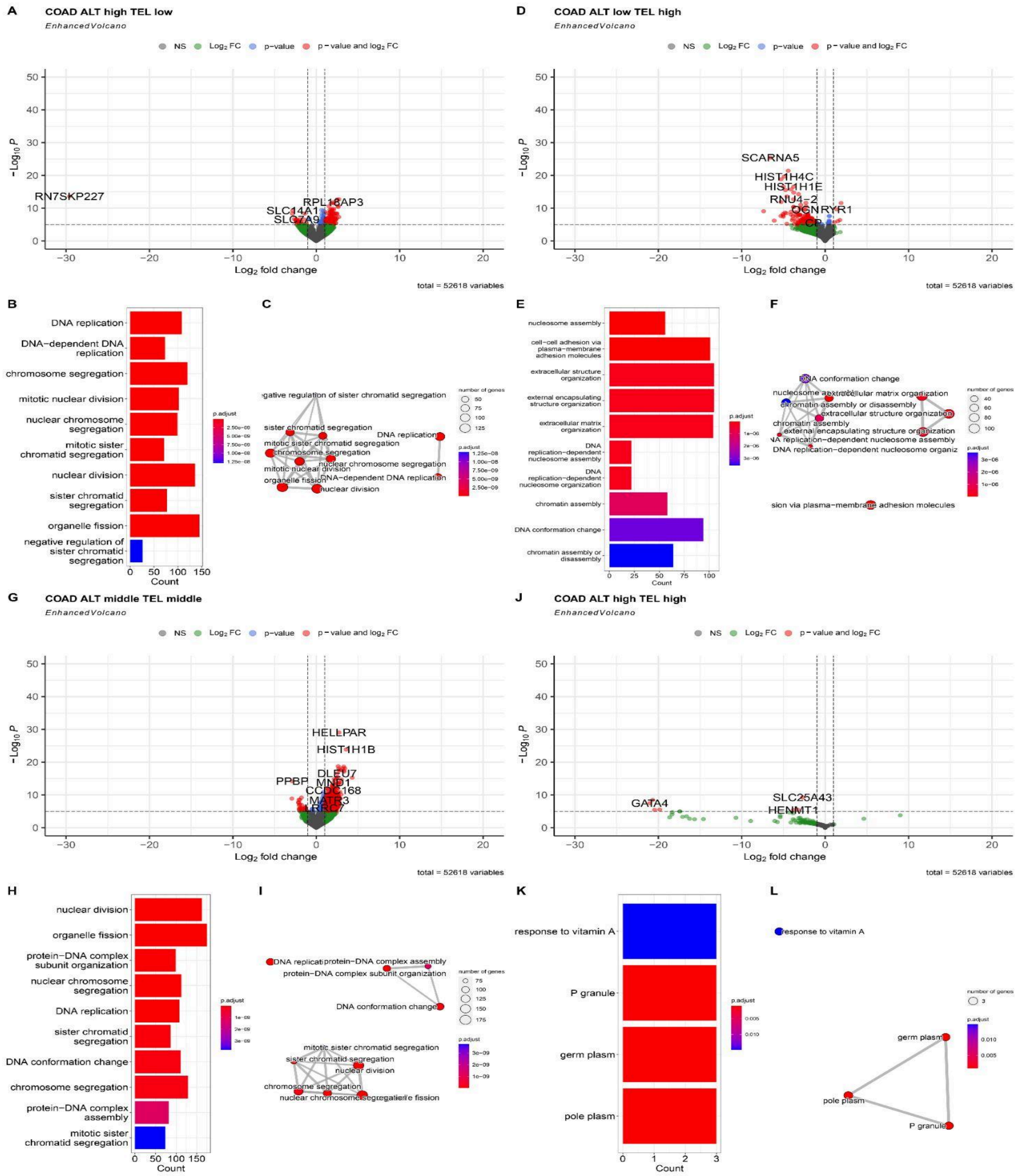

Figure S47. COAD DEG results and GO over-represented terms. (**A**) Volcano plot of differentially expressed genes (DEGs) for ALT ^{high} TEL ^{low} phenotype. (**B**) Barplot displays the top GO terms by Adjusted p-value. (**C**) Enrichmap clusters the most significant (by padj) GO terms to visualize relationships between terms. (**D**) Volcano plot of differentially expressed genes (DEGs) for ALT ^{low} TEL ^{high} phenotype. (**E**) Barplot displays the top GO terms by Adjusted p-value. (**F**) Enrichmap clusters the most significant (by padj) GO terms to visualize relationships between terms. (**G**) Volcano plot of differentially expressed genes (DEGs) for ALT ^{middle} TEL ^{middle} phenotype. (**H**) Barplot displays the top GO terms by Adjusted p-value. (**I**) Enrichmap clusters the most significant (by padj) GO terms to visualize relationships between terms. (**J**) Volcano plot of differentially expressed genes (DEGs) for ALT ^{high} TEL^{high} phenotype. (**K**) Barplot displays the top GO terms by Adjusted p-value. (**L**) Enrichmap clusters the most significant (by padj) GO terms to visualize relationships between terms. Matched normal samples have been removed.

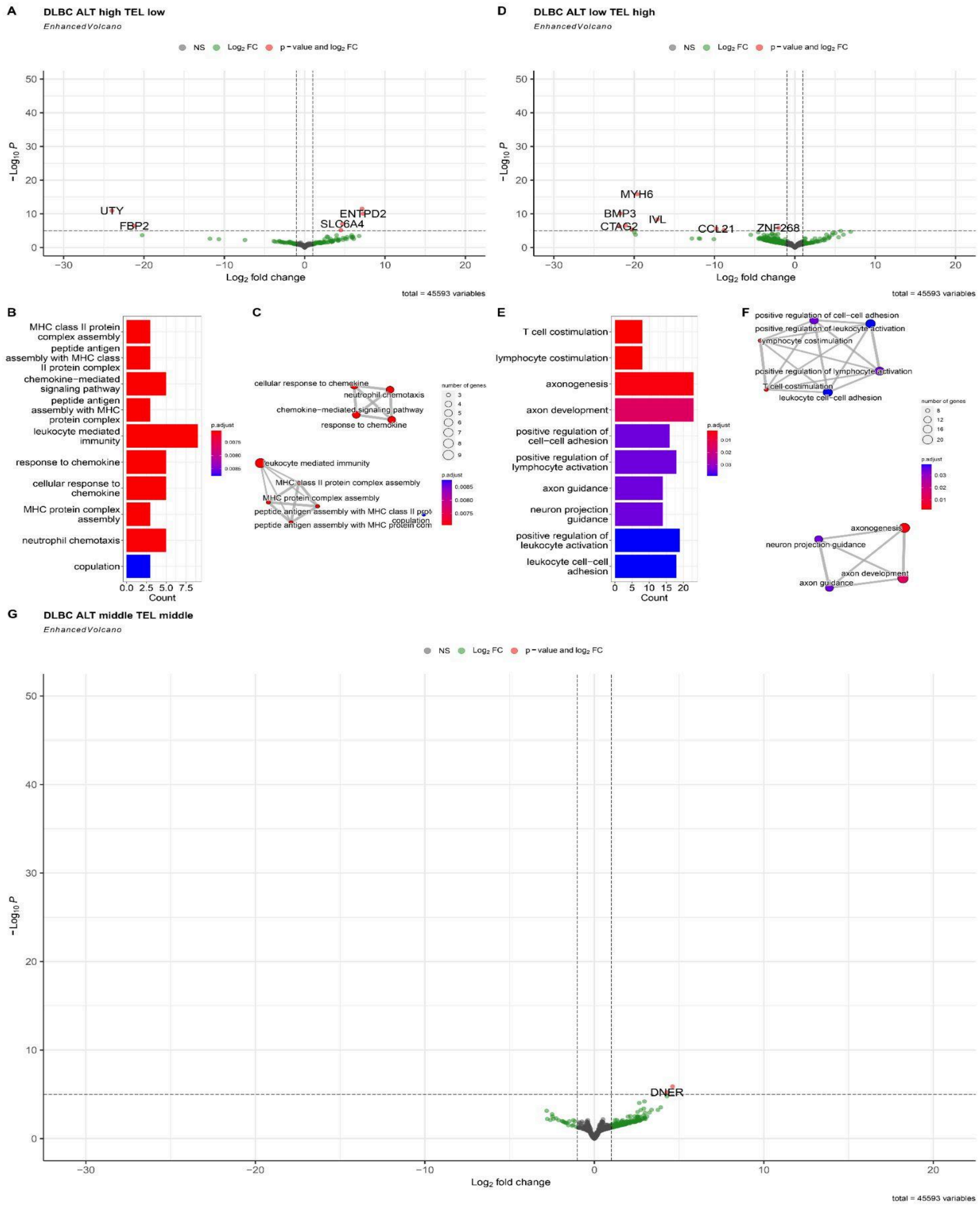

Figure S48. DLBC DEG results and GO over-represented terms. (**A**) Volcano plot of differentially expressed genes (DEGs) for ALT ^{high} TEL ^{low} phenotype. (**B**) Barplot displays the top GO terms by Adjusted p-value. (**C**) Enrichmap clusters the most significant (by padj) GO terms to visualize relationships between terms. (**D**) Volcano plot of differentially expressed genes (DEGs) for ALT ^{low} TEL ^{high} phenotype. (**E**) Barplot displays the top GO terms by Adjusted p-value. (**F**) Enrichmap clusters the most significant (by padj) GO terms to visualize relationships between terms. (**G**) Volcano plot of differentially expressed genes (DEGs) for ALT ^{middle} TEL ^{middle} phenotype. Over-represented terms were missing. Matched normal samples have been removed.

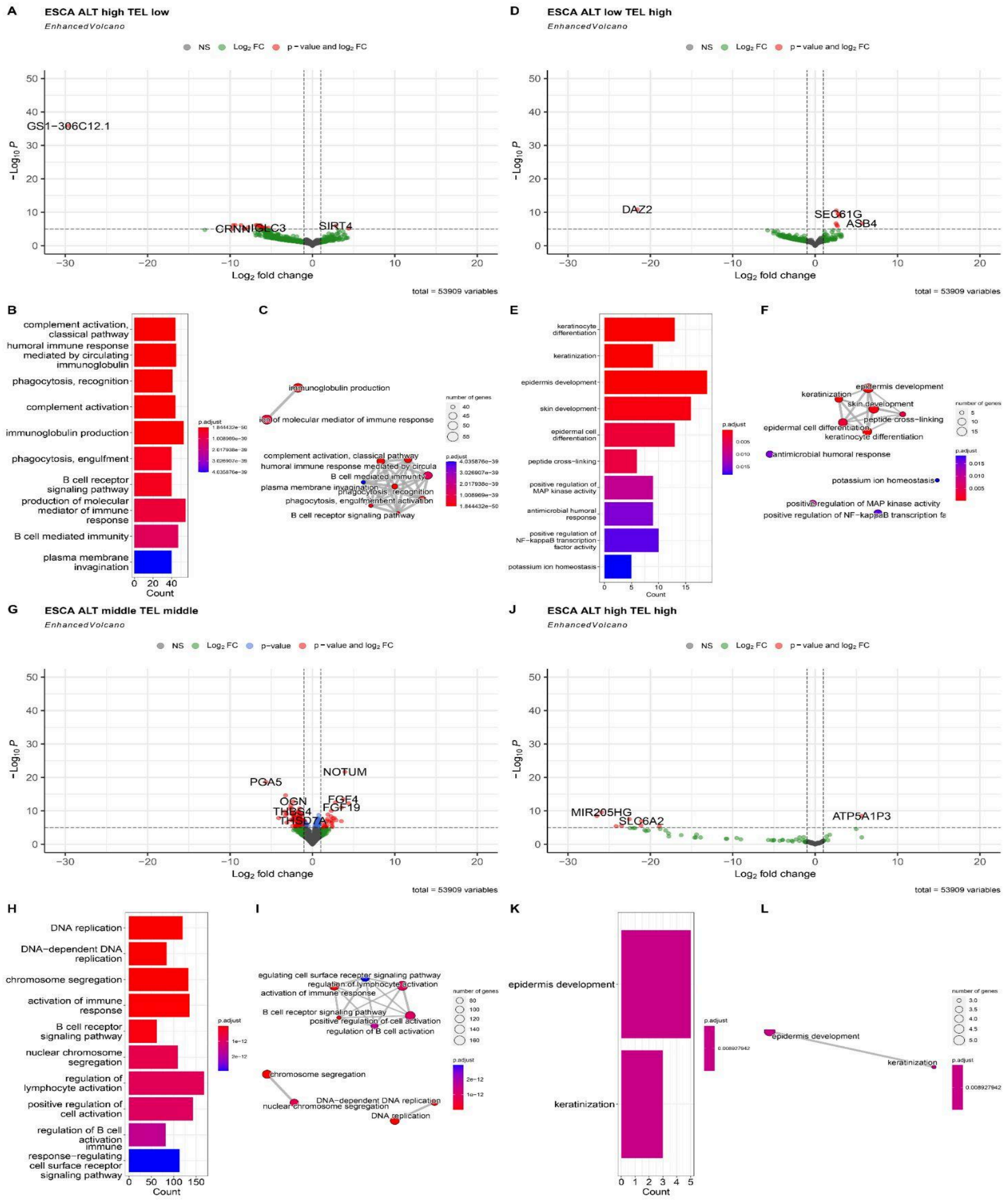

Figure S49. ESCA DEG results and GO over-represented terms. (**A**) Volcano plot of differentially expressed genes (DEGs) for ALT ^{high} TEL ^{low} phenotype. (**B**) Barplot displays the top GO terms by Adjusted p-value. (**C**) Enrichmap clusters the most significant (by padj) GO terms to visualize relationships between terms. (**D**) Volcano plot of differentially expressed genes (DEGs) for ALT ^{low} TEL ^{high} phenotype. (**E**) Barplot displays the top GO terms by Adjusted p-value. (**F**) Enrichmap clusters the most significant (by padj) GO terms to visualize relationships between terms. (**G**) Volcano plot of differentially expressed genes (DEGs) for ALT ^{middle} TEL ^{middle} phenotype. (**H**) Barplot displays the top GO terms by Adjusted p-value. (**I**) Enrichmap clusters the most significant (by padj) GO terms to visualize relationships between terms. (**J**) Volcano plot of differentially expressed genes (DEGs) for ALT ^{high} TEL^{high} phenotype. (**K**) Barplot displays the top GO terms by Adjusted p-value. (**L**) Enrichmap clusters the most significant (by padj) GO terms to visualize relationships between terms. Matched normal samples have been removed.

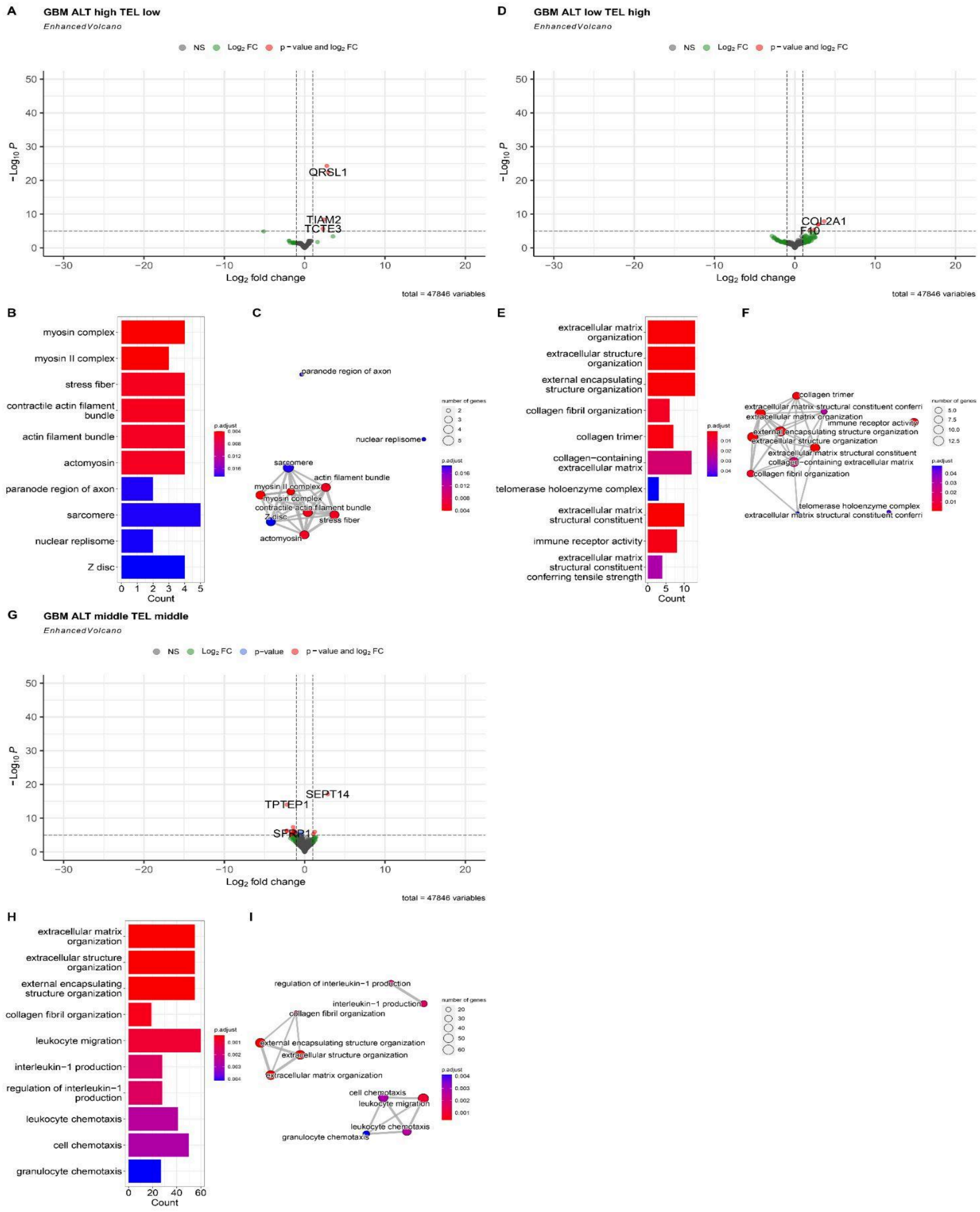

Figure S50. GBM DEG results and GO over-represented terms. (**A**) Volcano plot of differentially expressed genes (DEGs) for ALT ^{high} TEL ^{low} phenotype. (**B**) Barplot displays the top GO terms by Adjusted p-value. (**C**) Enrichmap clusters the most significant (by padj) GO terms to visualize relationships between terms. (**D**) Volcano plot of differentially expressed genes (DEGs) for ALT ^{low} TEL ^{high} phenotype. (**E**) Barplot displays the top GO terms by Adjusted p-value. (**F**) Enrichmap clusters the most significant (by padj) GO terms to visualize relationships between terms. (**G**) Volcano plot of differentially expressed genes (DEGs) for ALT ^{middle} TEL ^{middle} phenotype. (**H**) Barplot displays the top GO terms by Adjusted p-value. (**I**) Enrichmap clusters the most significant (by padj) GO terms to visualize relationships between terms. Matched normal samples have been removed.

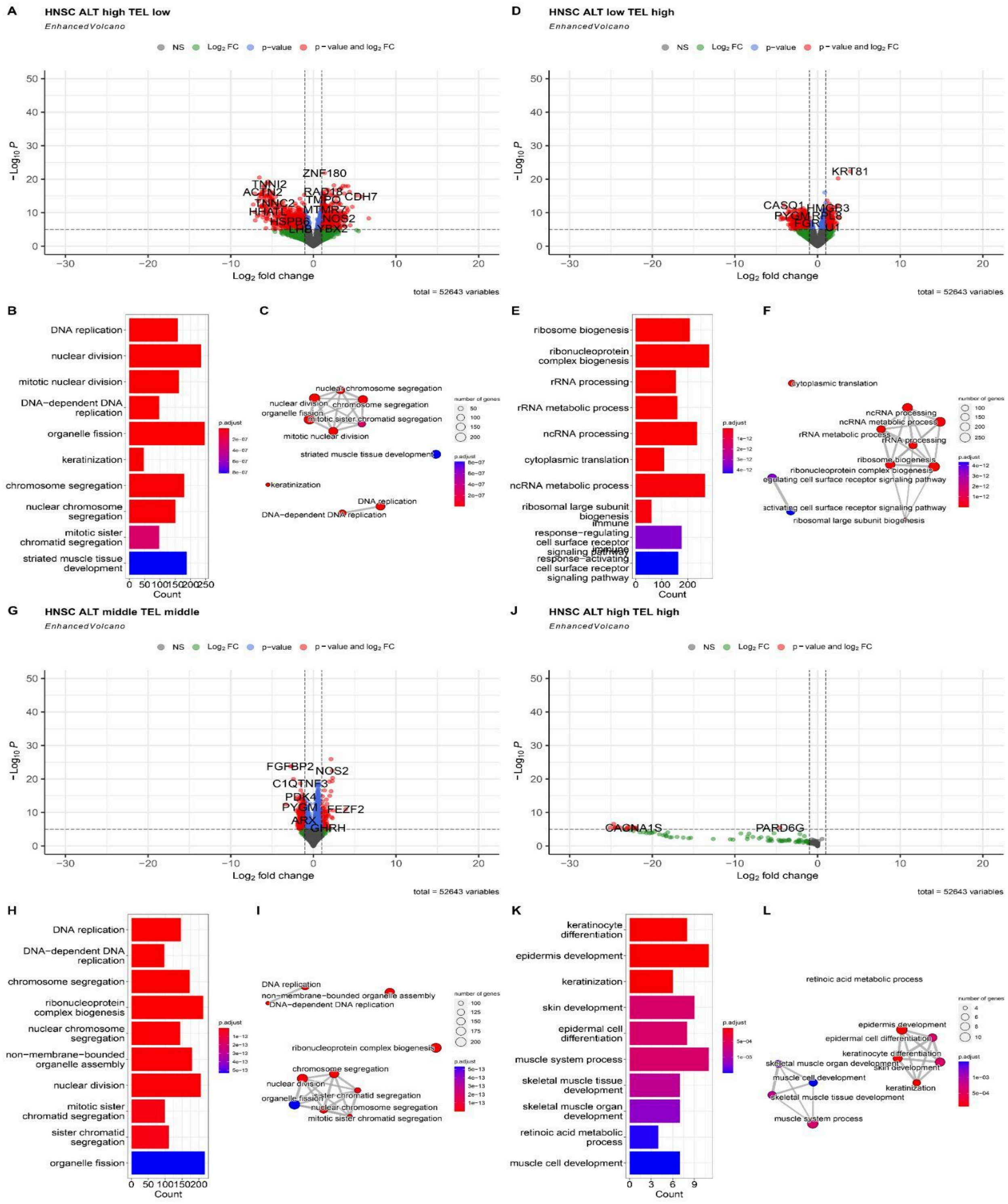

Figure S51. HNSC DEG results and GO over-represented terms. (**A**) Volcano plot of differentially expressed genes (DEGs) for ALT ^{high} TEL ^{low} phenotype. (**B**) Barplot displays the top GO terms by Adjusted p-value. (**C**) Enrichmap clusters the most significant (by padj) GO terms to visualize relationships between terms. (**D**) Volcano plot of differentially expressed genes (DEGs) for ALT ^{low} TEL ^{high} phenotype. (**E**) Barplot displays the top GO terms by Adjusted p-value. (**F**) Enrichmap clusters the most significant (by padj) GO terms to visualize relationships between terms. (**G**) Volcano plot of differentially expressed genes (DEGs) for ALT ^{middle} TEL ^{middle} phenotype. (**H**) Barplot displays the top GO terms by Adjusted p-value. (**I**) Enrichmap clusters the most significant (by padj) GO terms to visualize relationships between terms. (**J**) Volcano plot of differentially expressed genes (DEGs) for ALT ^{high} TEL^{high} phenotype. (**K**) Barplot displays the top GO terms by Adjusted p-value. (**L**) Enrichmap clusters the most significant (by padj) GO terms to visualize relationships between terms. Matched normal samples have been removed.

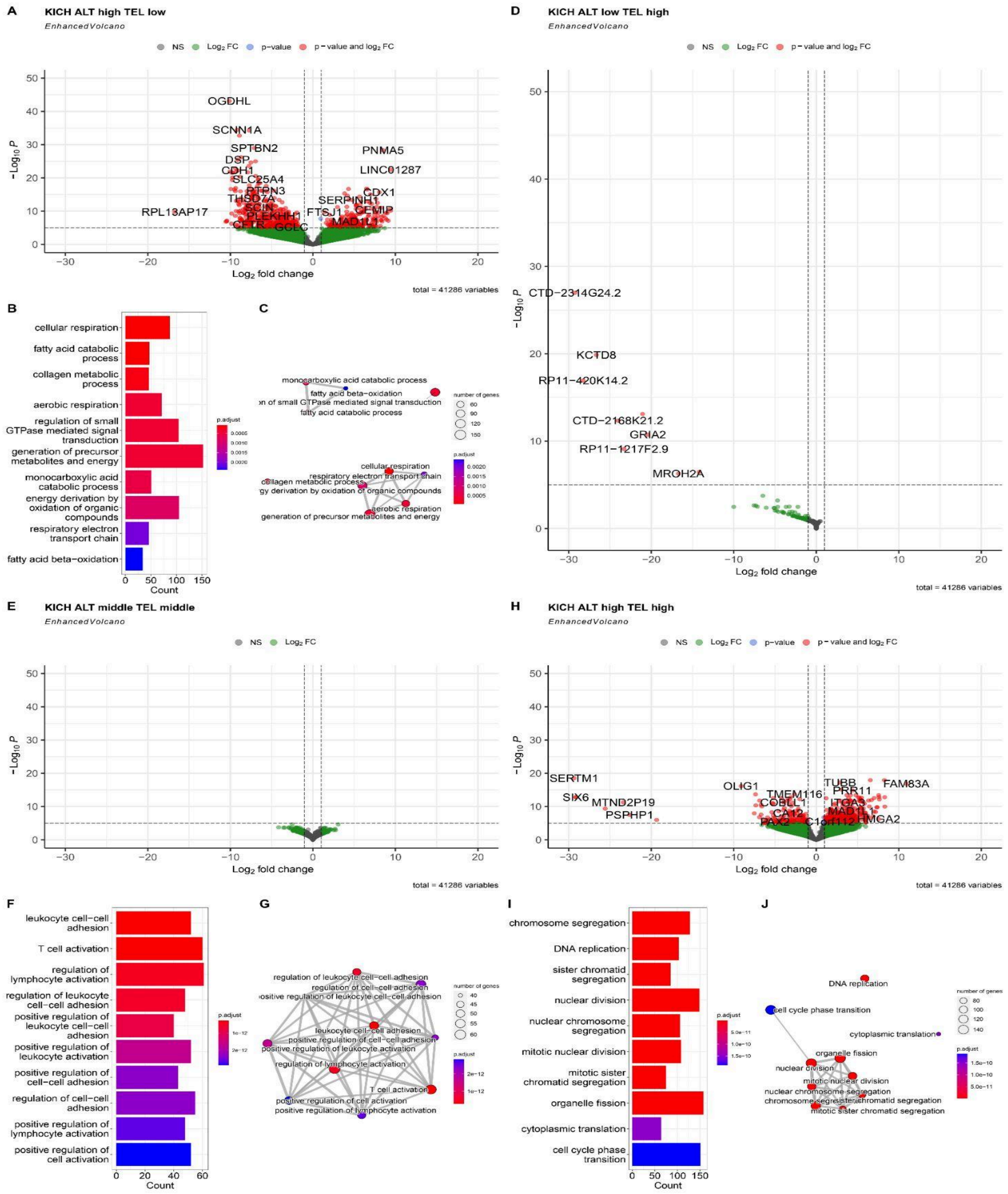

Figure S52. KIHC DEG results and GO over-represented terms. (**A**) Volcano plot of differentially expressed genes (DEGs) for ALT ^{high} TEL ^{low} phenotype. (**B**) Barplot displays the top GO terms by Adjusted p-value. (**C**) Enrichmap clusters the most significant (by padj) GO terms to visualize relationships between terms. (**D**) Volcano plot of differentially expressed genes (DEGs) for ALT ^{low} TEL ^{high} phenotype. Over-represented terms were missing. (**E**) Volcano plot of differentially expressed genes (DEGs) for ALT ^{middle} TEL ^{middle} phenotype. (**F**) Barplot displays the top GO terms by Adjusted p-value. (**G**) Enrichmap clusters the most significant (by padj) GO terms to visualize relationships between terms. (**H**) Volcano plot of differentially expressed genes (DEGs) for ALT ^{high} TEL^{high} phenotype. (**I**) Barplot displays the top GO terms by Adjusted p-value. (**J**) Enrichmap clusters the most significant (by padj) GO terms to visualize relationships between terms. Matched normal samples have been removed.

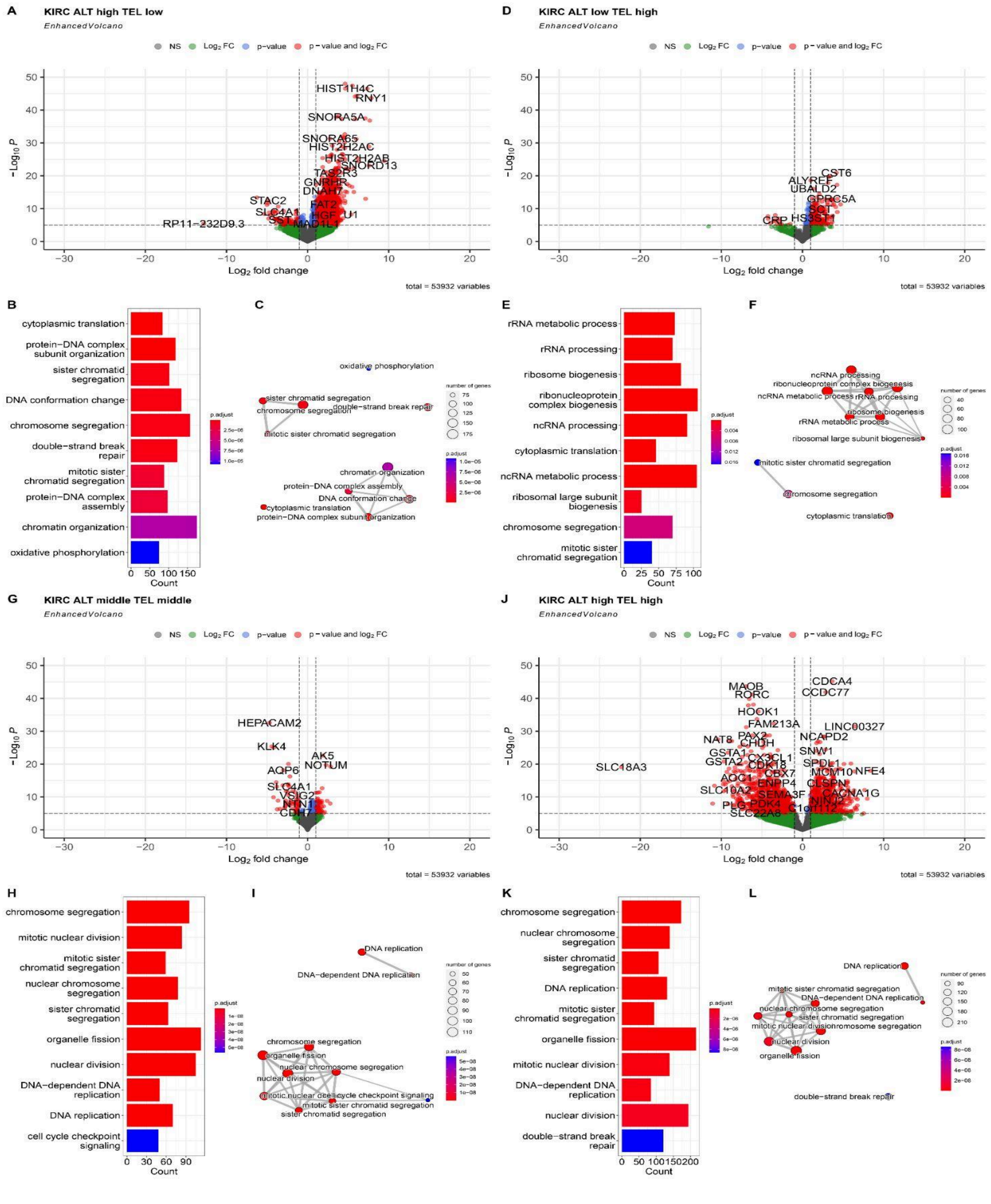

Figure S53. KIRC DEG results and GO over-represented terms. (**A**) Volcano plot of differentially expressed genes (DEGs) for ALT ^{high} TEL ^{low} phenotype. (**B**) Barplot displays the top GO terms by Adjusted p-value. (**C**) Enrichmap clusters the most significant (by padj) GO terms to visualize relationships between terms. (**D**) Volcano plot of differentially expressed genes (DEGs) for ALT ^{low} TEL ^{high} phenotype. (**E**) Barplot displays the top GO terms by Adjusted p-value. (**F**) Enrichmap clusters the most significant (by padj) GO terms to visualize relationships between terms. (**G**) Volcano plot of differentially expressed genes (DEGs) for ALT ^{middle} TEL ^{middle} phenotype. (**H**) Barplot displays the top GO terms by Adjusted p-value. (**I**) Enrichmap clusters the most significant (by padj) GO terms to visualize relationships between terms. (**J**) Volcano plot of differentially expressed genes (DEGs) for ALT ^{high} TEL^{high} phenotype. (**K**) Barplot displays the top GO terms by Adjusted p-value. (**L**) Enrichmap clusters the most significant (by padj) GO terms to visualize relationships between terms. Matched normal samples have been removed.

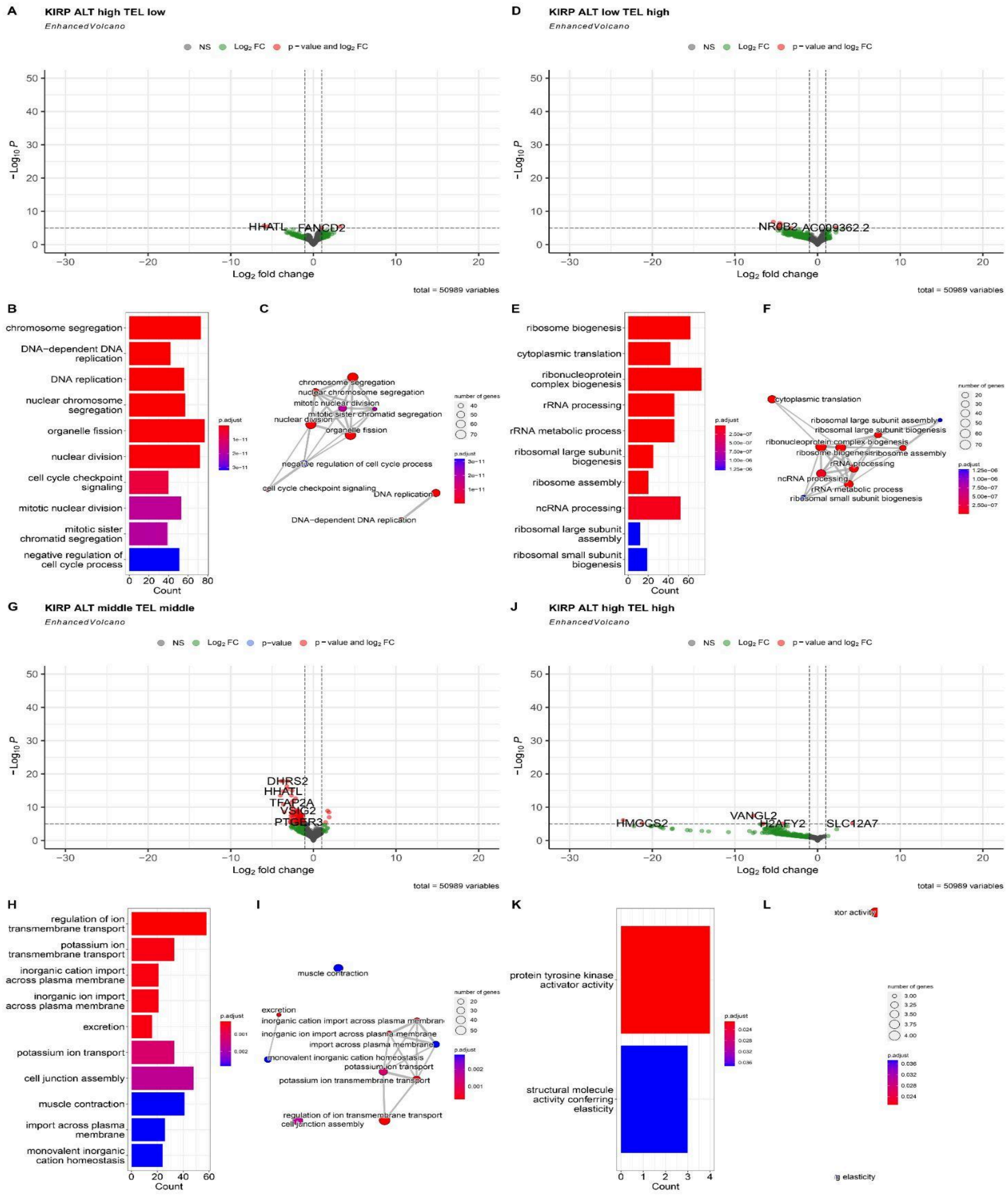

Figure S54. KIRP DEG results and GO over-represented terms. (**A**) Volcano plot of differentially expressed genes (DEGs) for ALT ^{high} TEL ^{low} phenotype. (**B**) Barplot displays the top GO terms by Adjusted p-value. (**C**) Enrichmap clusters the most significant (by padj) GO terms to visualize relationships between terms. (**D**) Volcano plot of differentially expressed genes (DEGs) for ALT ^{low} TEL ^{high} phenotype. (**E**) Barplot displays the top GO terms by Adjusted p-value. (**F**) Enrichmap clusters the most significant (by padj) GO terms to visualize relationships between terms. (**G**) Volcano plot of differentially expressed genes (DEGs) for ALT ^{middle} TEL ^{middle} phenotype. (**H**) Barplot displays the top GO terms by Adjusted p-value. (**I**) Enrichmap clusters the most significant (by padj) GO terms to visualize relationships between terms. (**J**) Volcano plot of differentially expressed genes (DEGs) for ALT ^{high} TEL^{high} phenotype. (**K**) Barplot displays the top GO terms by Adjusted p-value. (**L**) Enrichmap clusters the most significant (by padj) GO terms to visualize relationships between terms. Matched normal samples have been removed.

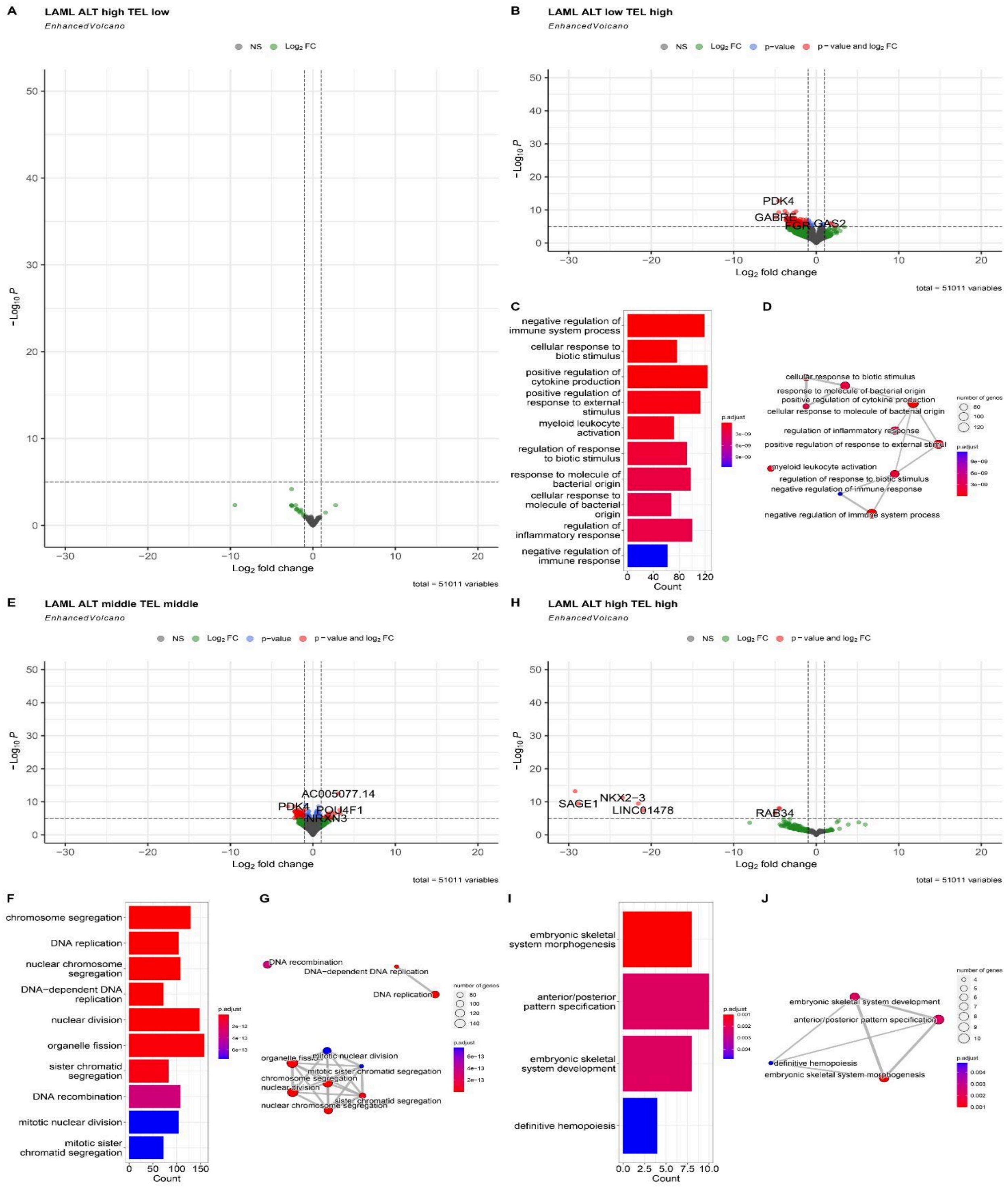

Figure S55. LAML DEG results and GO over-represented terms. (**A**) Volcano plot of differentially expressed genes (DEGs) for ALT ^{high} TEL ^{low} phenotype. Over-represented terms were missing. (**B**) Volcano plot of differentially expressed genes (DEGs) for ALT ^{low} TEL ^{high} phenotype. (**C**) Barplot displays the top GO terms by Adjusted p-value. Only one term "signal release" was over-represented. (**D**) Enrichmap clusters the most significant (by padj) GO terms to visualize relationships between terms. Only one term "signal release" was over-represented. (**E**) Volcano plot of differentially expressed genes (DEGs) for ALT ^{middle} TEL ^{middle} phenotype. (**F**) Barplot displays the top GO terms by Adjusted p-value. (**G**) Enrichmap clusters the most significant (by padj) GO terms to visualize relationships between terms. (**H**) Volcano plot of differentially expressed genes (DEGs) for ALT ^{high} TEL^{high} phenotype. (**I**) Barplot displays the top GO terms by Adjusted p-value. (**J**) Enrichmap clusters the most significant (by padj) GO terms to visualize relationships between terms. Matched normal samples have been removed.

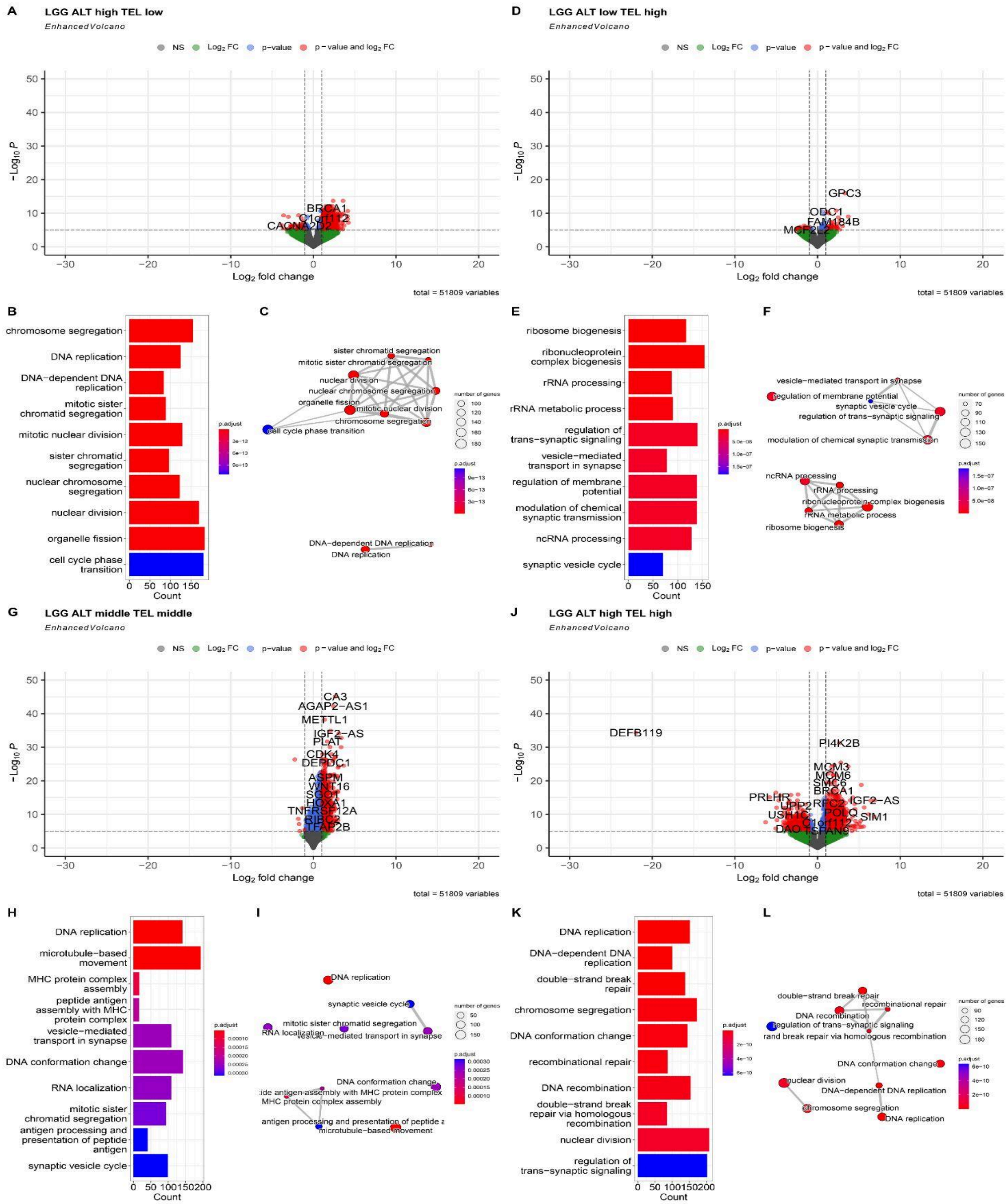

Figure S56. LGG DEG results and GO over-represented terms. (**A**) Volcano plot of differentially expressed genes (DEGs) for ALT ^{high} TEL ^{low} phenotype. (**B**) Barplot displays the top GO terms by Adjusted p-value. (**C**) Enrichmap clusters the most significant (by padj) GO terms to visualize relationships between terms. (**D**) Volcano plot of differentially expressed genes (DEGs) for ALT ^{low} TEL ^{high} phenotype. (**E**) Barplot displays the top GO terms by Adjusted p-value. (**F**) Enrichmap clusters the most significant (by padj) GO terms to visualize relationships between terms. (**G**) Volcano plot of differentially expressed genes (DEGs) for ALT ^{middle} TEL ^{middle} phenotype. (**H**) Barplot displays the top GO terms by Adjusted p-value. (**I**) Enrichmap clusters the most significant (by padj) GO terms to visualize relationships between terms. (**J**) Volcano plot of differentially expressed genes (DEGs) for ALT ^{high} TEL^{high} phenotype. (**K**) Barplot displays the top GO terms by Adjusted p-value. (**L**) Enrichmap clusters the most significant (by padj) GO terms to visualize relationships between terms. Matched normal samples have been removed.

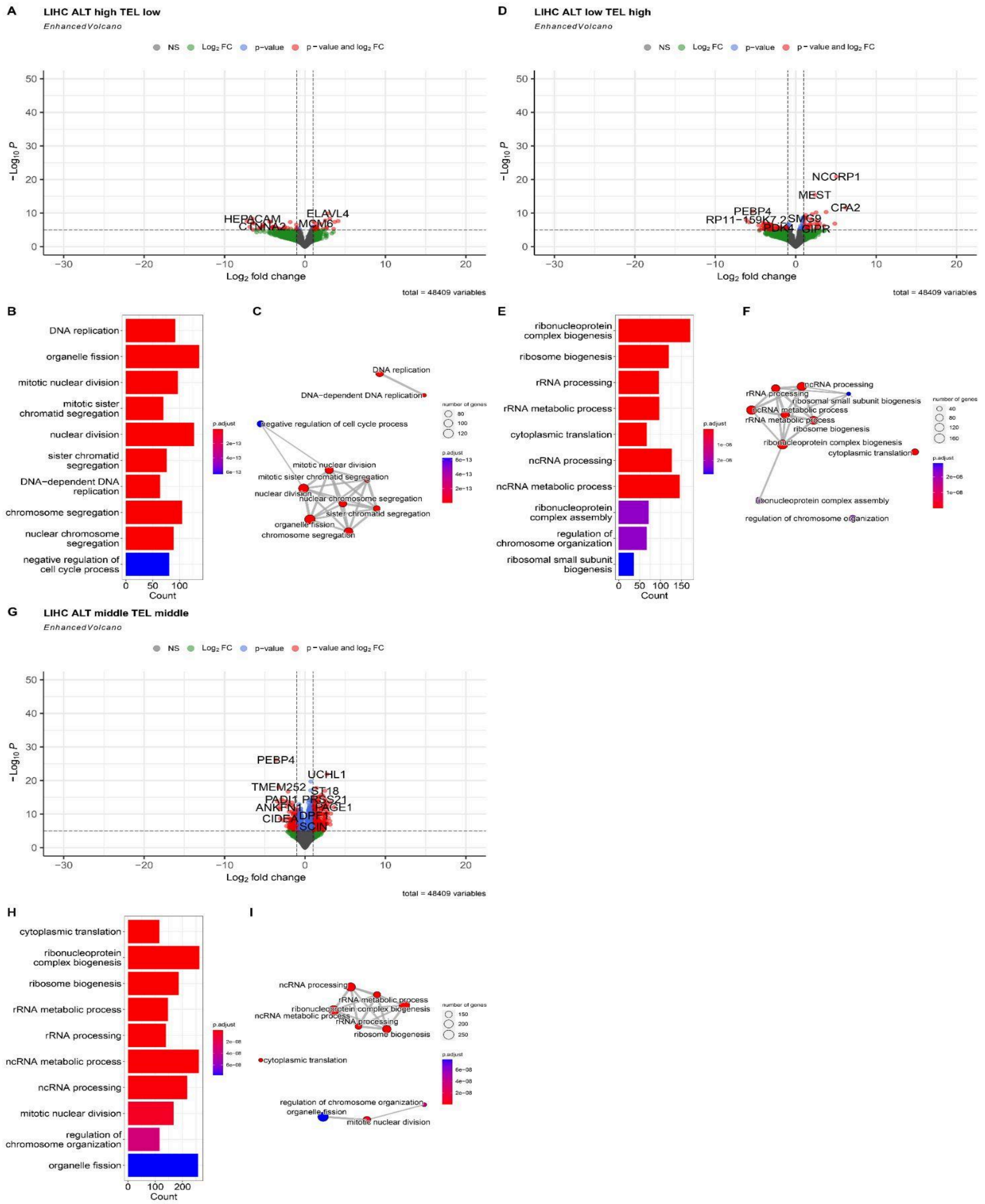

Figure S57. LIHC DEG results and GO over-represented terms. (**A**) Volcano plot of differentially expressed genes (DEGs) for ALT ^{high} TEL ^{low} phenotype. (**B**) Barplot displays the top GO terms by Adjusted p-value. (**C**) Enrichmap clusters the most significant (by padj) GO terms to visualize relationships between terms. (**D**) Volcano plot of differentially expressed genes (DEGs) for ALT ^{low} TEL ^{high} phenotype. (**E**) Barplot displays the top GO terms by Adjusted p-value. (**F**) Enrichmap clusters the most significant (by padj) GO terms to visualize relationships between terms. (**G**) Volcano plot of differentially expressed genes (DEGs) for ALT ^{middle} TEL ^{middle} phenotype. (**H**) Barplot displays the top GO terms by Adjusted p-value. (**I**) Enrichmap clusters the most significant (by padj) GO terms to visualize relationships between terms. Matched normal samples have been removed.

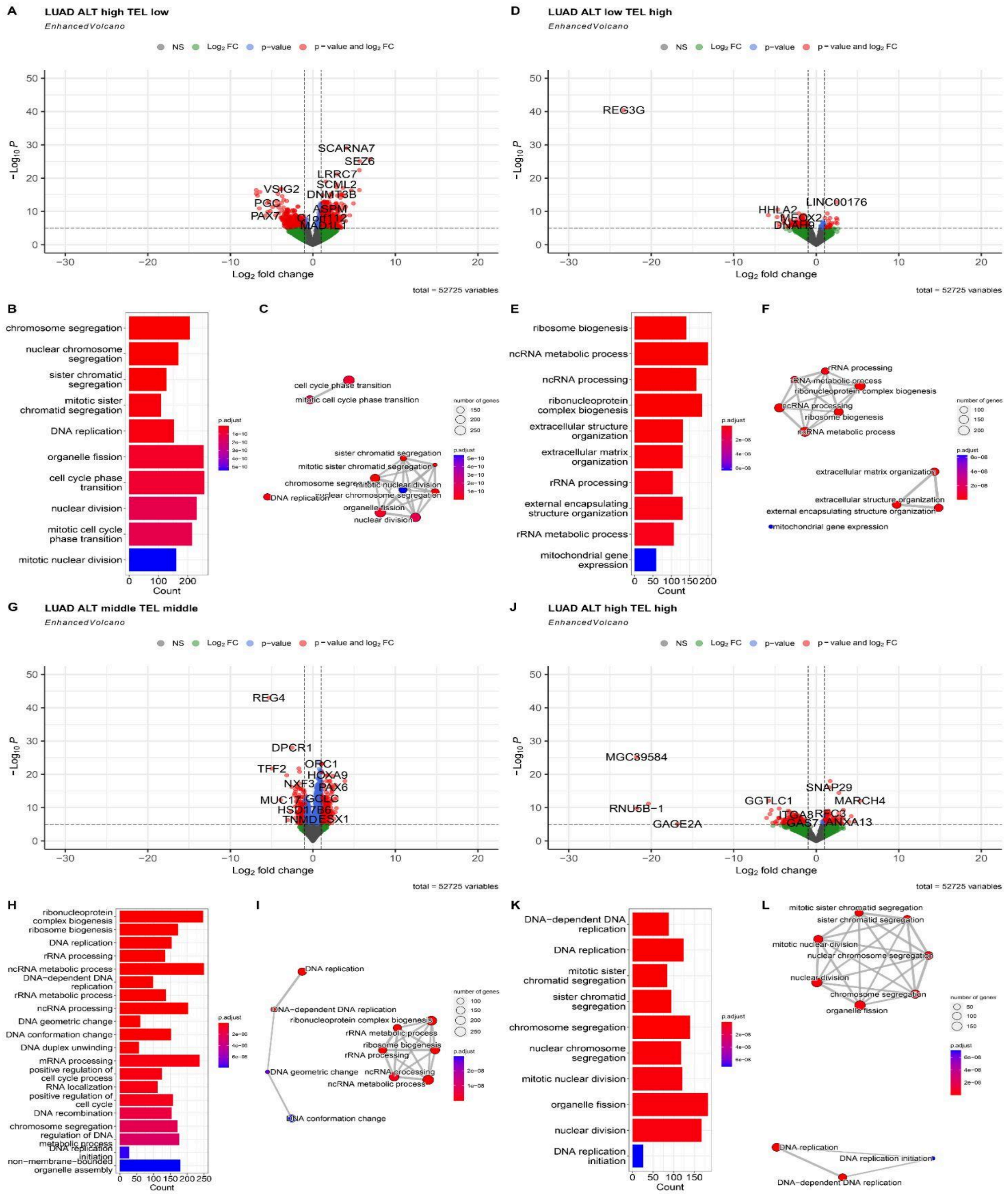

Figure S58. LUAD DEG results and GO over-represented terms. (**A**) Volcano plot of differentially expressed genes (DEGs) for ALT ^{high} TEL ^{low} phenotype. (**B**) Barplot displays the top GO terms by Adjusted p-value. (**C**) Enrichmap clusters the most significant (by padj) GO terms to visualize relationships between terms. (**D**) Volcano plot of differentially expressed genes (DEGs) for ALT ^{low} TEL ^{high} phenotype. (**E**) Barplot displays the top GO terms by Adjusted p-value. (**F**) Enrichmap clusters the most significant (by padj) GO terms to visualize relationships between terms. (**G**) Volcano plot of differentially expressed genes (DEGs) for ALT ^{middle} TEL ^{middle} phenotype. (**H**) Barplot displays the top GO terms by Adjusted p-value. (**I**) Enrichmap clusters the most significant (by padj) GO terms to visualize relationships between terms. (**J**) Volcano plot of differentially expressed genes (DEGs) for ALT ^{high} TEL^{high} phenotype. (**K**) Barplot displays the top GO terms by Adjusted p-value. (**L**) Enrichmap clusters the most significant (by padj) GO terms to visualize relationships between terms. Matched normal samples have been removed.

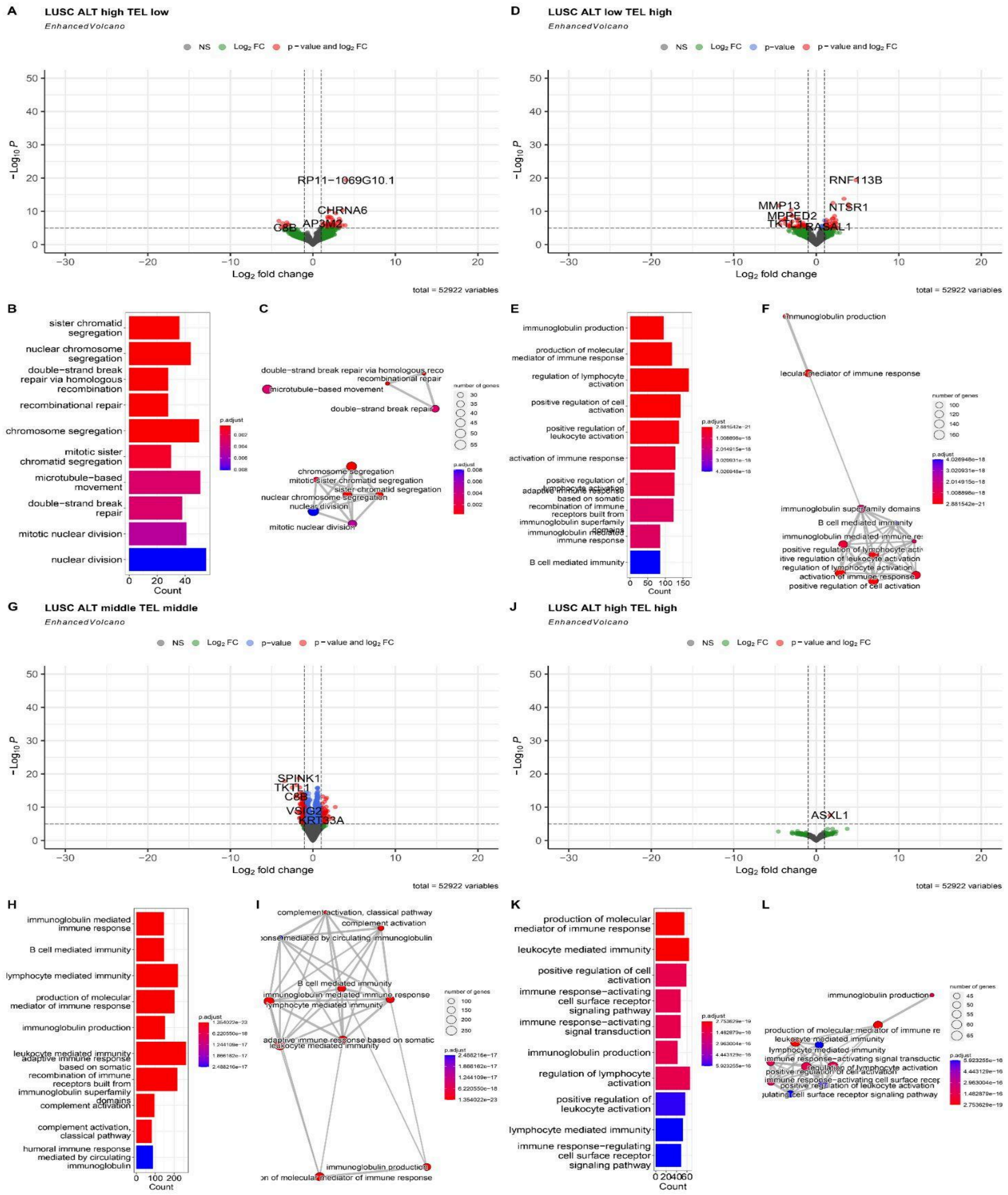

Figure S59. LUSC DEG results and GO over-represented terms. (**A**) Volcano plot of differentially expressed genes (DEGs) for ALT ^{high} TEL ^{low} phenotype. (**B**) Barplot displays the top GO terms by Adjusted p-value. (**C**) Enrichmap clusters the most significant (by padj) GO terms to visualize relationships between terms. (**D**) Volcano plot of differentially expressed genes (DEGs) for ALT ^{low} TEL ^{high} phenotype. (**E**) Barplot displays the top GO terms by Adjusted p-value. (**F**) Enrichmap clusters the most significant (by padj) GO terms to visualize relationships between terms. (**G**) Volcano plot of differentially expressed genes (DEGs) for ALT ^{middle} TEL ^{middle} phenotype. (**H**) Barplot displays the top GO terms by Adjusted p-value. (**I**) Enrichmap clusters the most significant (by padj) GO terms to visualize relationships between terms. (**J**) Volcano plot of differentially expressed genes (DEGs) for ALT ^{high} TEL^{high} phenotype. (**K**) Barplot displays the top GO terms by Adjusted p-value. (**L**) Enrichmap clusters the most significant (by padj) GO terms to visualize relationships between terms. Matched normal samples have been removed.

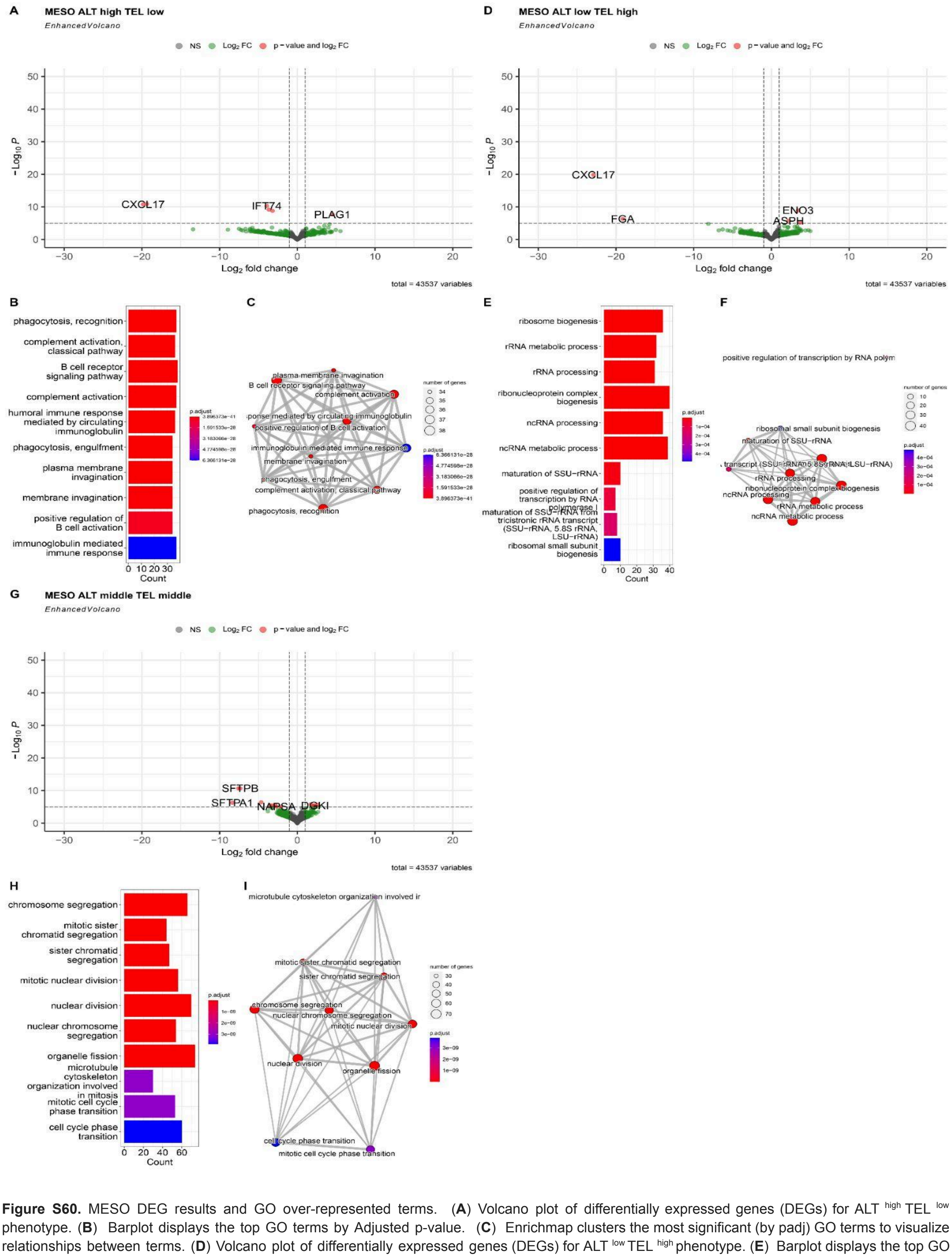

Figure S60. MESO DEG results and GO over-represented terms. (**A**) Volcano plot of differentially expressed genes (DEGs) for ALT ^{high} TEL ^{low} phenotype. (**B**) Barplot displays the top GO terms by Adjusted p-value. (**C**) Enrichmap clusters the most significant (by padj) GO terms to visualize relationships between terms. (**D**) Volcano plot of differentially expressed genes (DEGs) for ALT ^{low} TEL ^{high} phenotype. (**E**) Barplot displays the top GO terms by Adjusted p-value. (**F**) Enrichmap clusters the most significant (by padj) GO terms to visualize relationships between terms. (**G**) Volcano plot of differentially expressed genes (DEGs) for ALT ^{middle} TEL ^{middle} phenotype. (**H**) Barplot displays the top GO terms by Adjusted p-value. (**I**) Enrichmap clusters the most significant (by padj) GO terms to visualize relationships between terms. Matched normal samples have been removed.

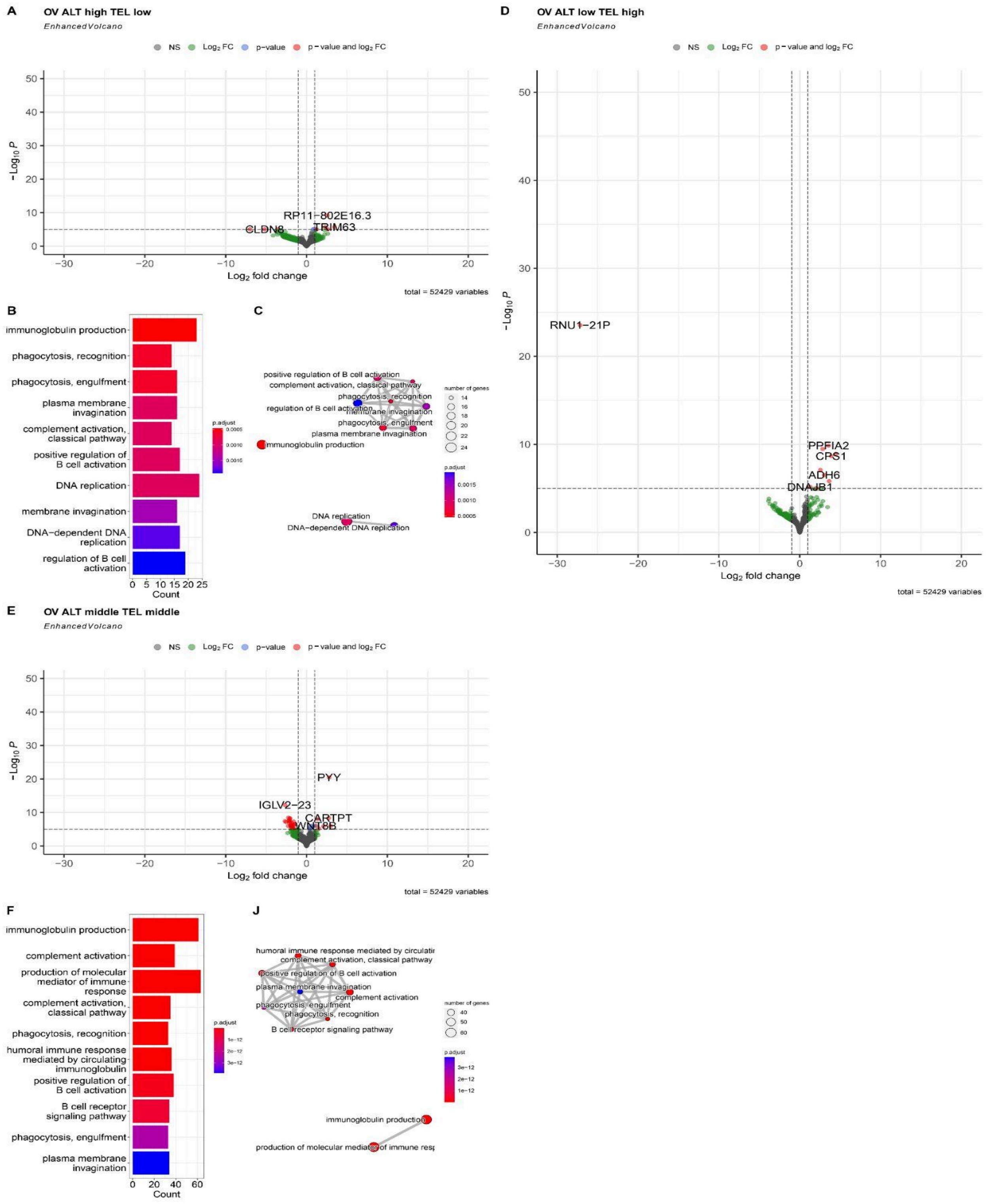

Figure S61. OV DEG results and GO over-represented terms. (**A**) Volcano plot of differentially expressed genes (DEGs) for ALT ^{high} TEL ^{low} phenotype. (**B**) Barplot displays the top GO terms by Adjusted p-value. (**C**) Enrichmap clusters the most significant (by padj) GO terms to visualize relationships between terms. (**D**) Volcano plot of differentially expressed genes (DEGs) for ALT ^{low} TEL ^{high} phenotype. Over-represented terms were missing. (**E**) Volcano plot of differentially expressed genes (DEGs) for ALT ^{middle} TEL ^{middle} phenotype. (**F**) Barplot displays the top GO terms by Adjusted p-value. (**J**) Enrichmap clusters the most significant (by padj) GO terms to visualize relationships between terms. Matched normal samples have been removed.

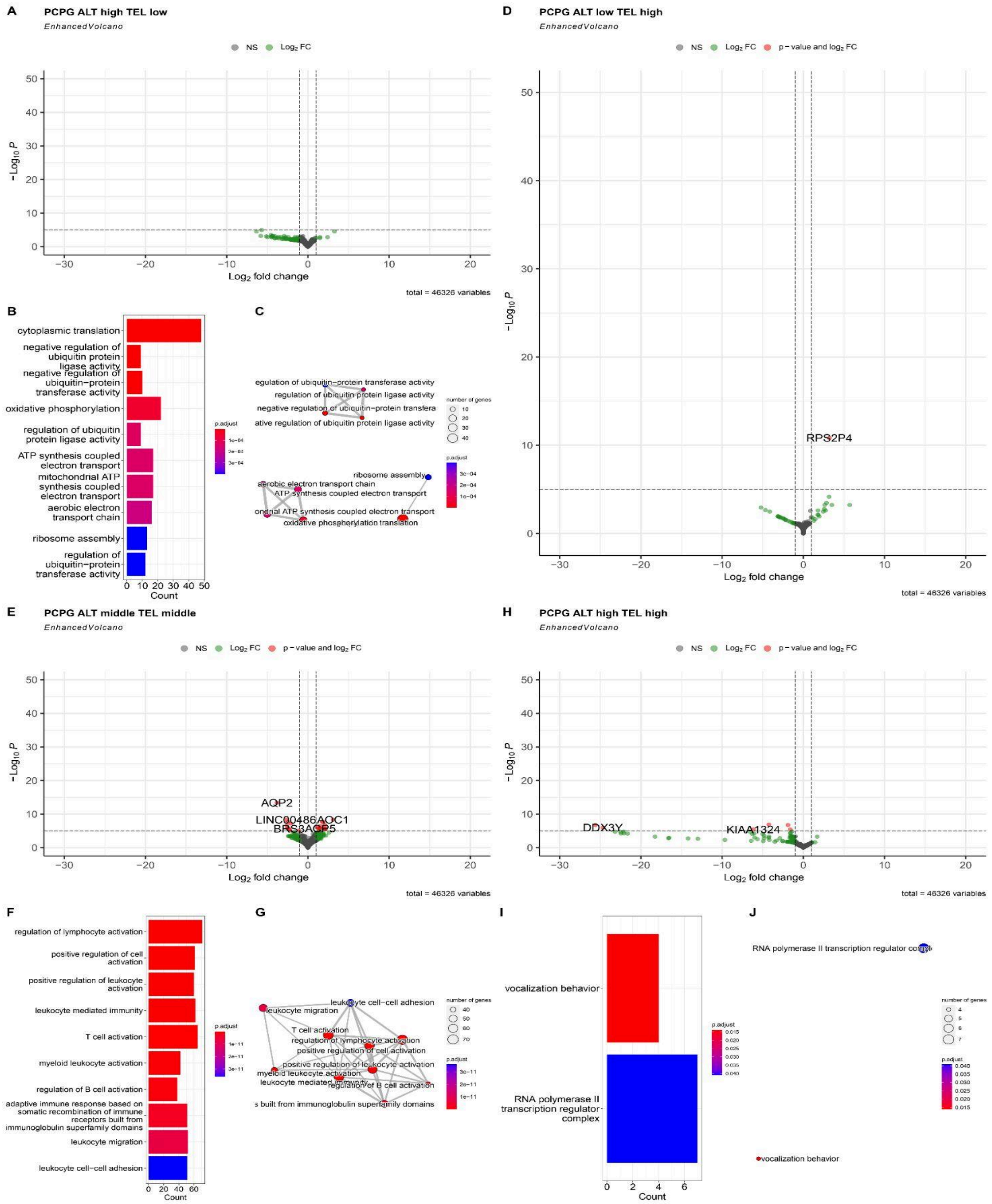

Figure S62. PCPG DEG results and GO over-represented terms. (**A**) Volcano plot of differentially expressed genes (DEGs) for ALT ^{high} TEL ^{low} phenotype. (**B**) Barplot displays the top GO terms by Adjusted p-value. (**C**) Enrichmap clusters the most significant (by padj) GO terms to visualize relationships between terms. (**D**) Volcano plot of differentially expressed genes (DEGs) for ALT ^{low} TEL ^{high} phenotype. Over-represented terms were missing. (**E**) Volcano plot of differentially expressed genes (DEGs) for ALT ^{middle} TEL ^{middle} phenotype. (**F**) Barplot displays the top GO terms by Adjusted p-value. (**J**) Enrichmap clusters the most significant (by padj) GO terms to visualize relationships between terms.(**H**) Volcano plot of differentially expressed genes (DEGs) for ALT ^{high} TEL^{high} phenotype. (**I**) Barplot displays the top GO terms by Adjusted p-value. (**J**) Enrichmap clusters the most significant (by padj) GO terms to visualize relationships between terms. Matched normal samples have been removed.

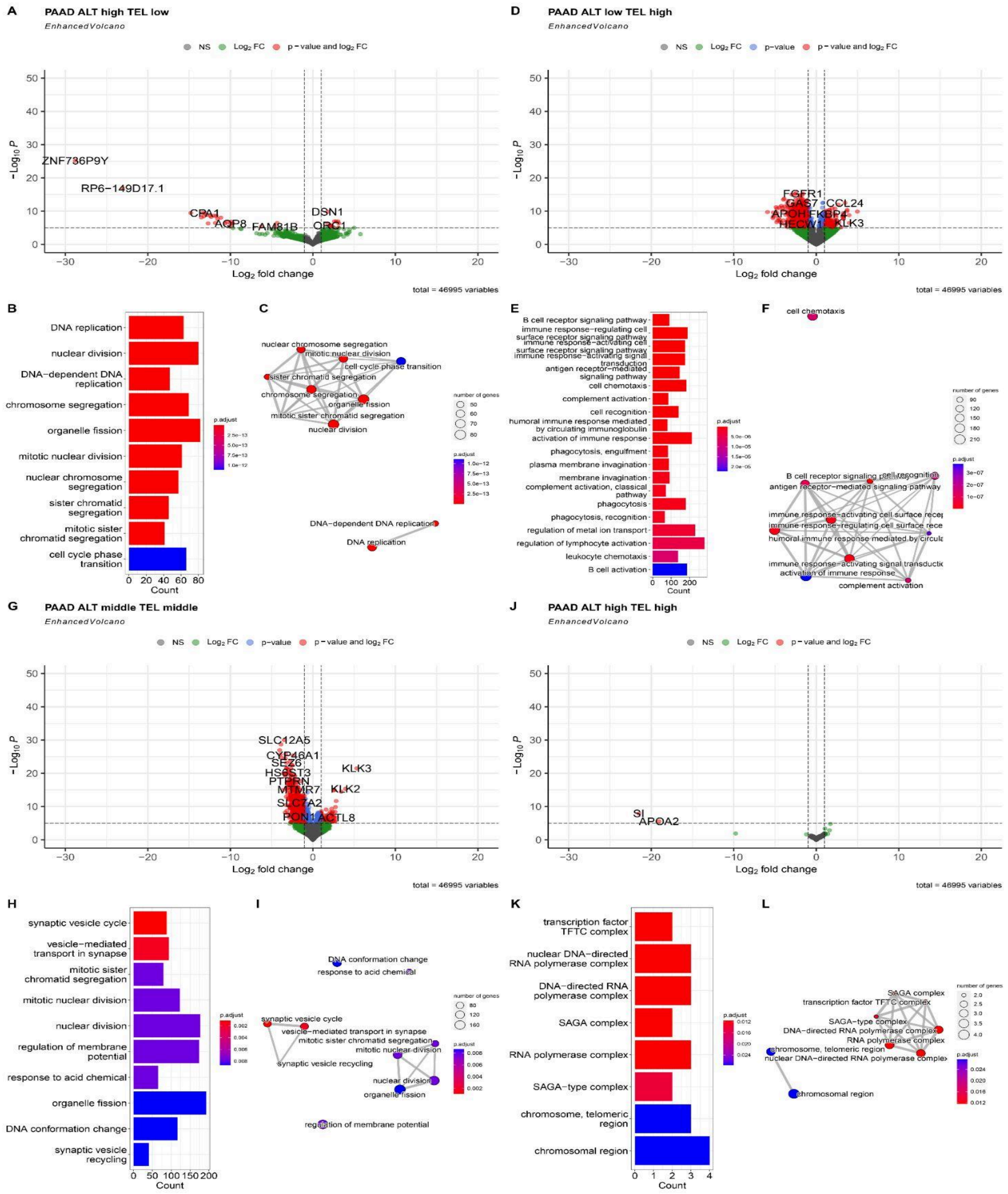

Figure S63. PAAD DEG results and GO over-represented terms. (**A**) Volcano plot of differentially expressed genes (DEGs) for ALT high TEL low phenotype. (**B**) Barplot displays the top GO terms by Adjusted p-value. (**C**) Enrichmap clusters the most significant (by padj) GO terms to visualize relationships between terms. (**D**) Volcano plot of differentially expressed genes (DEGs) for ALT low TEL high phenotype. (**E**) Barplot displays the top GO terms by Adjusted p-value. (**F**) Enrichmap clusters the most significant (by padj) GO terms to visualize relationships between terms. (**G**) Volcano plot of differentially expressed genes (DEGs) for ALT middle TEL middle phenotype. (**H**) Barplot displays the top GO terms by Adjusted p-value. (**I**) Enrichmap clusters the most significant (by padj) GO terms to visualize relationships between terms. (**J**) Volcano plot of differentially expressed genes (DEGs) for ALT high TEL high phenotype. (**K**) Barplot displays the top GO terms by Adjusted p-value. (**L**) Enrichmap clusters the most significant (by padj) GO terms to visualize relationships between terms. Matched normal samples have been removed.

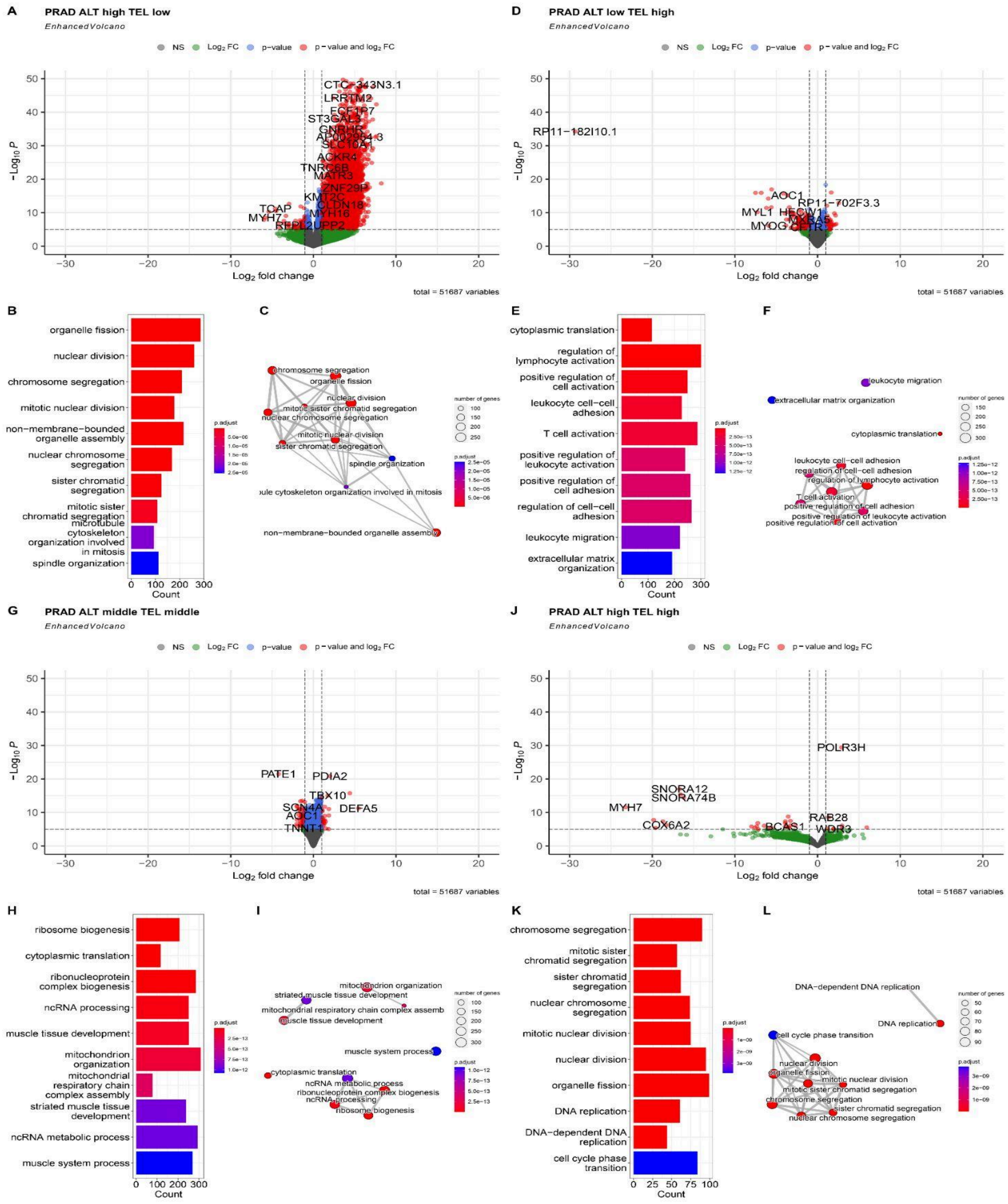

Figure S64. PRAD DEG results and GO over-represented terms. (**A**) Volcano plot of differentially expressed genes (DEGs) for ALT ^{high} TEL ^{low} phenotype. (**B**) Barplot displays the top GO terms by Adjusted p-value. (**C**) Enrichmap clusters the most significant (by padj) GO terms to visualize relationships between terms. (**D**) Volcano plot of differentially expressed genes (DEGs) for ALT ^{low} TEL ^{high} phenotype. (**E**) Barplot displays the top GO terms by Adjusted p-value. (**F**) Enrichmap clusters the most significant (by padj) GO terms to visualize relationships between terms. (**G**) Volcano plot of differentially expressed genes (DEGs) for ALT ^{middle} TEL ^{middle} phenotype. (**H**) Barplot displays the top GO terms by Adjusted p-value. (**I**) Enrichmap clusters the most significant (by padj) GO terms to visualize relationships between terms. (**J**) Volcano plot of differentially expressed genes (DEGs) for ALT ^{high} TEL^{high} phenotype. (**K**) Barplot displays the top GO terms by Adjusted p-value. (**L**) Enrichmap clusters the most significant (by padj) GO terms to visualize relationships between terms. Matched normal samples have been removed.

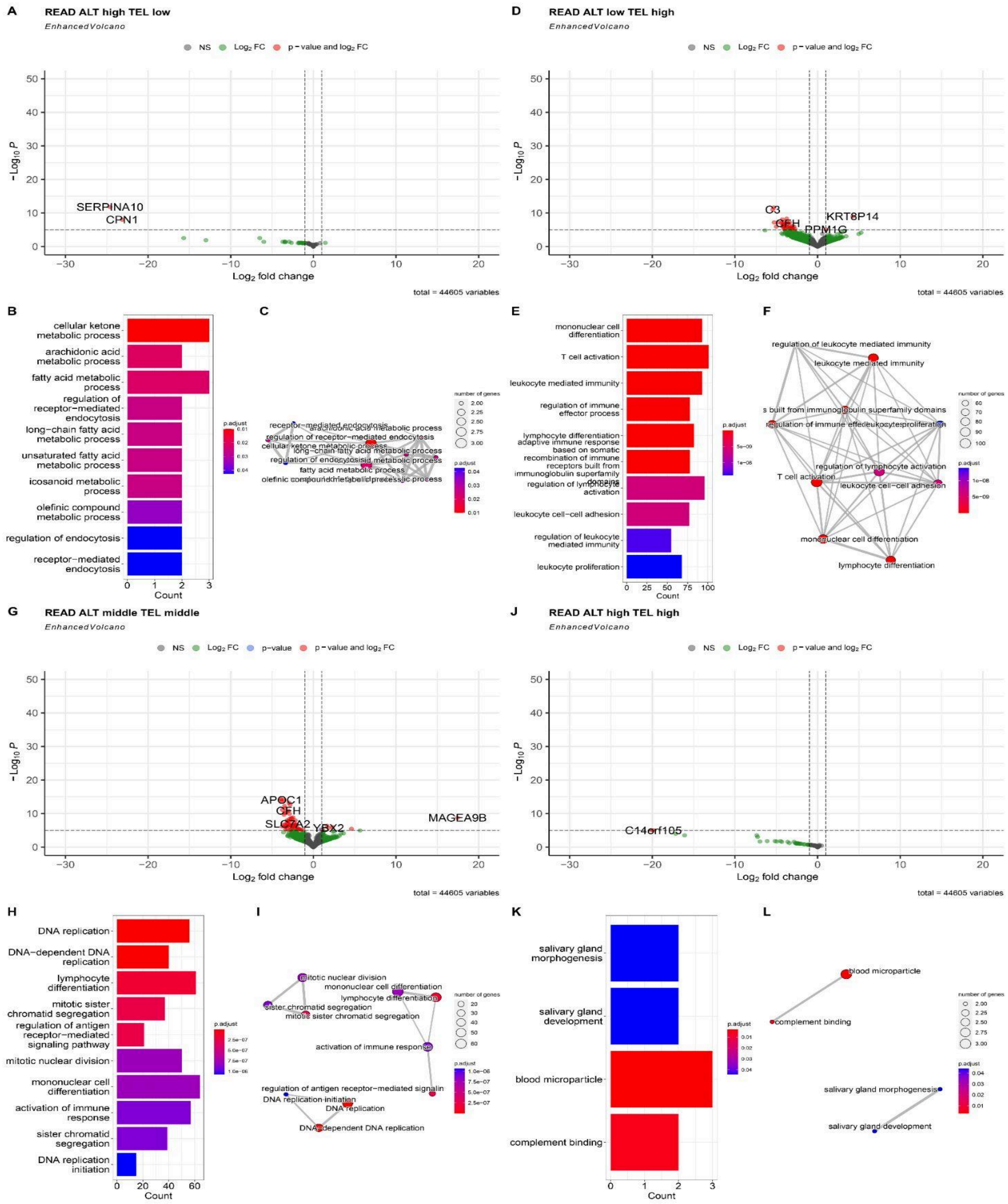

Figure S65. READ DEG results and GO over-represented terms. (**A**) Volcano plot of differentially expressed genes (DEGs) for ALT ^{high} TEL ^{low} phenotype. (**B**) Barplot displays the top GO terms by Adjusted p-value. (**C**) Enrichmap clusters the most significant (by padj) GO terms to visualize relationships between terms. (**D**) Volcano plot of differentially expressed genes (DEGs) for ALT ^{low} TEL ^{high} phenotype. (**E**) Barplot displays the top GO terms by Adjusted p-value. (**F**) Enrichmap clusters the most significant (by padj) GO terms to visualize relationships between terms. (**G**) Volcano plot of differentially expressed genes (DEGs) for ALT ^{middle} TEL ^{middle} phenotype. (**H**) Barplot displays the top GO terms by Adjusted p-value. (**I**) Enrichmap clusters the most significant (by padj) GO terms to visualize relationships between terms. (**J**) Volcano plot of differentially expressed genes (DEGs) for ALT ^{high} TEL^{high} phenotype. (**K**) Barplot displays the top GO terms by Adjusted p-value. (**L**) Enrichmap clusters the most significant (by padj) GO terms to visualize relationships between terms. Matched normal samples have been removed.

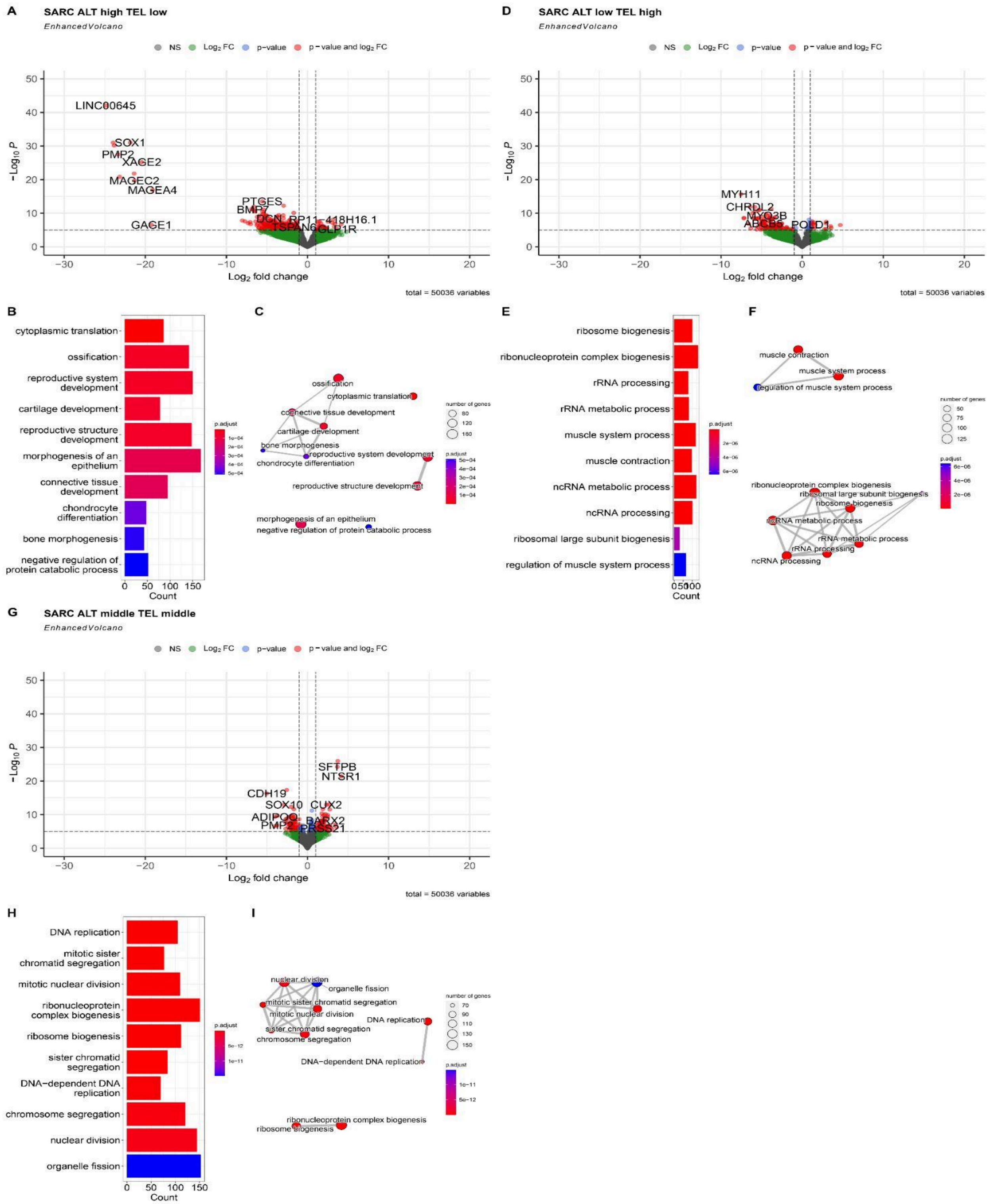

Figure S66. SARC DEG results and GO over-represented terms. (**A**) Volcano plot of differentially expressed genes (DEGs) for ALT ^{high} TEL ^{low} phenotype. (**B**) Barplot displays the top GO terms by Adjusted p-value. (**C**) Enrichmap clusters the most significant (by padj) GO terms to visualize relationships between terms. (**D**) Volcano plot of differentially expressed genes (DEGs) for ALT ^{low} TEL ^{high} phenotype. (**E**) Barplot displays the top GO terms by Adjusted p-value. (**F**) Enrichmap clusters the most significant (by padj) GO terms to visualize relationships between terms. (**G**) Volcano plot of differentially expressed genes (DEGs) for ALT ^{middle} TEL ^{middle} phenotype. (**H**) Barplot displays the top GO terms by Adjusted p-value. (**I**) Enrichmap clusters the most significant (by padj) GO terms to visualize relationships between terms.

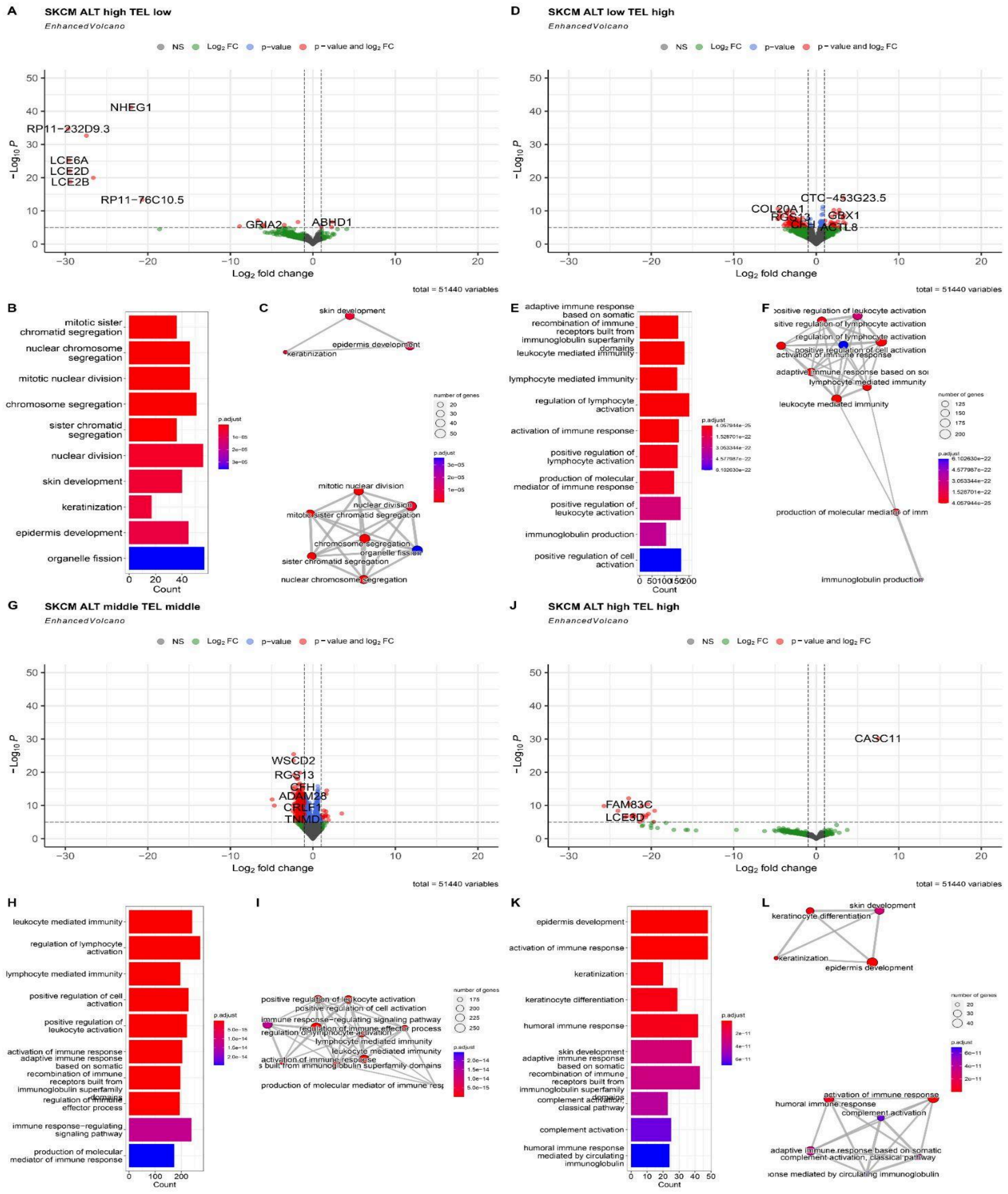

Figure S67. SKCM DEG results and GO over-represented terms. (**A**) Volcano plot of differentially expressed genes (DEGs) for ALT ^{high} TEL ^{low} phenotype. (**B**) Barplot displays the top GO terms by Adjusted p-value. (**C**) Enrichmap clusters the most significant (by padj) GO terms to visualize relationships between terms. (**D**) Volcano plot of differentially expressed genes (DEGs) for ALT ^{low} TEL ^{high} phenotype. (**E**) Barplot displays the top GO terms by Adjusted p-value. (**F**) Enrichmap clusters the most significant (by padj) GO terms to visualize relationships between terms. (**G**) Volcano plot of differentially expressed genes (DEGs) for ALT ^{middle} TEL ^{middle} phenotype. (**H**) Barplot displays the top GO terms by Adjusted p-value. (**I**) Enrichmap clusters the most significant (by padj) GO terms to visualize relationships between terms. (**J**) Volcano plot of differentially expressed genes (DEGs) for ALT ^{high} TEL^{high} phenotype. (**K**) Barplot displays the top GO terms by Adjusted p-value. (**L**) Enrichmap clusters the most significant (by padj) GO terms to visualize relationships between terms. Matched normal samples have been removed.

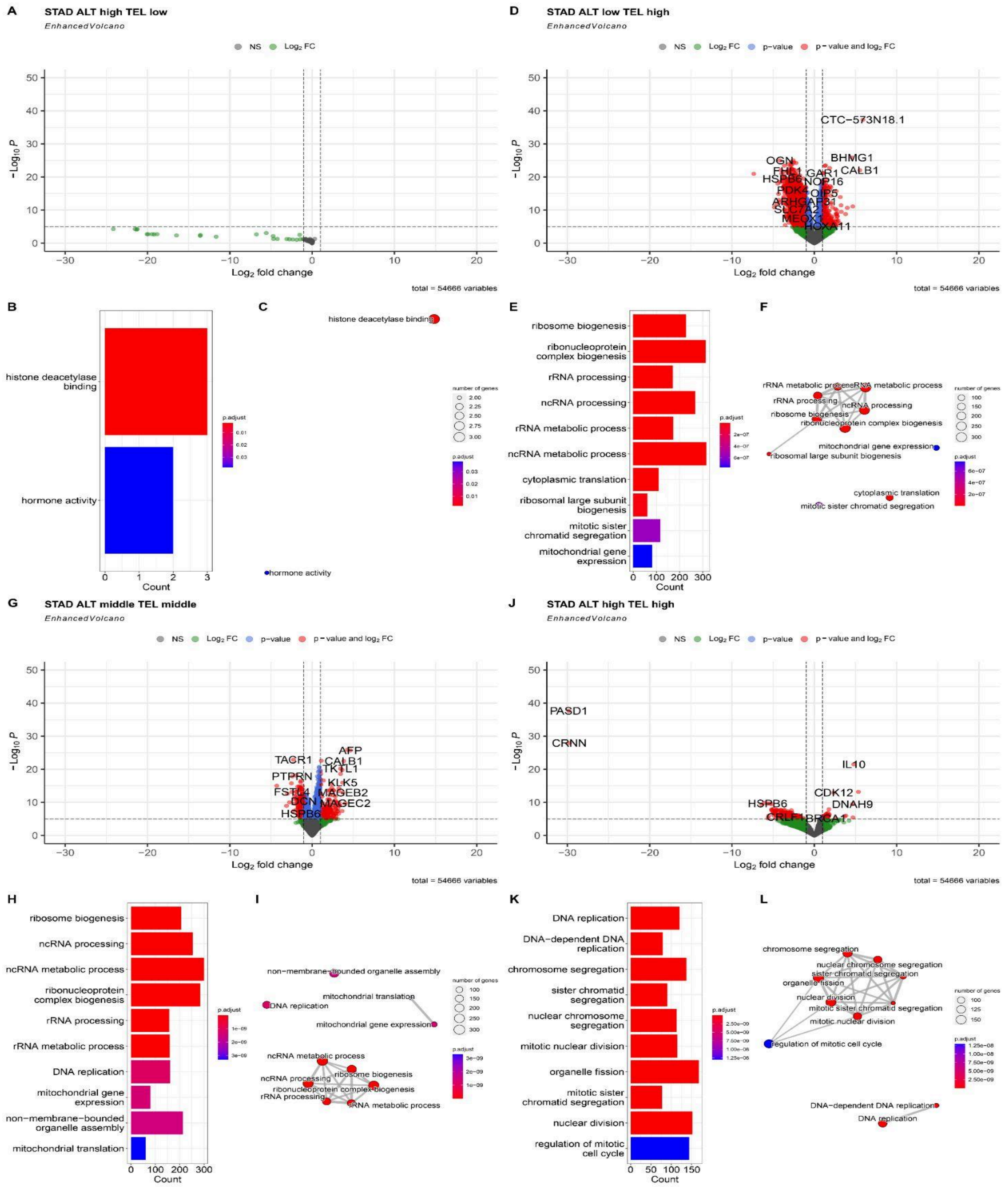

Figure S68. STAD DEG results and GO over-represented terms. (**A**) Volcano plot of differentially expressed genes (DEGs) for ALT ^{high} TEL ^{low} phenotype. (**B**) Barplot displays the top GO terms by Adjusted p-value. (**C**) Enrichmap clusters the most significant (by padj) GO terms to visualize relationships between terms. (**D**) Volcano plot of differentially expressed genes (DEGs) for ALT ^{low} TEL ^{high} phenotype. (**E**) Barplot displays the top GO terms by Adjusted p-value. (**F**) Enrichmap clusters the most significant (by padj) GO terms to visualize relationships between terms. (**G**) Volcano plot of differentially expressed genes (DEGs) for ALT ^{middle} TEL ^{middle} phenotype. (**H**) Barplot displays the top GO terms by Adjusted p-value. (**I**) Enrichmap clusters the most significant (by padj) GO terms to visualize relationships between terms. (**J**) Volcano plot of differentially expressed genes (DEGs) for ALT ^{high} TEL^{high} phenotype. (**K**) Barplot displays the top GO terms by Adjusted p-value. (**L**) Enrichmap clusters the most significant (by padj) GO terms to visualize relationships between terms. Matched normal samples have been removed.

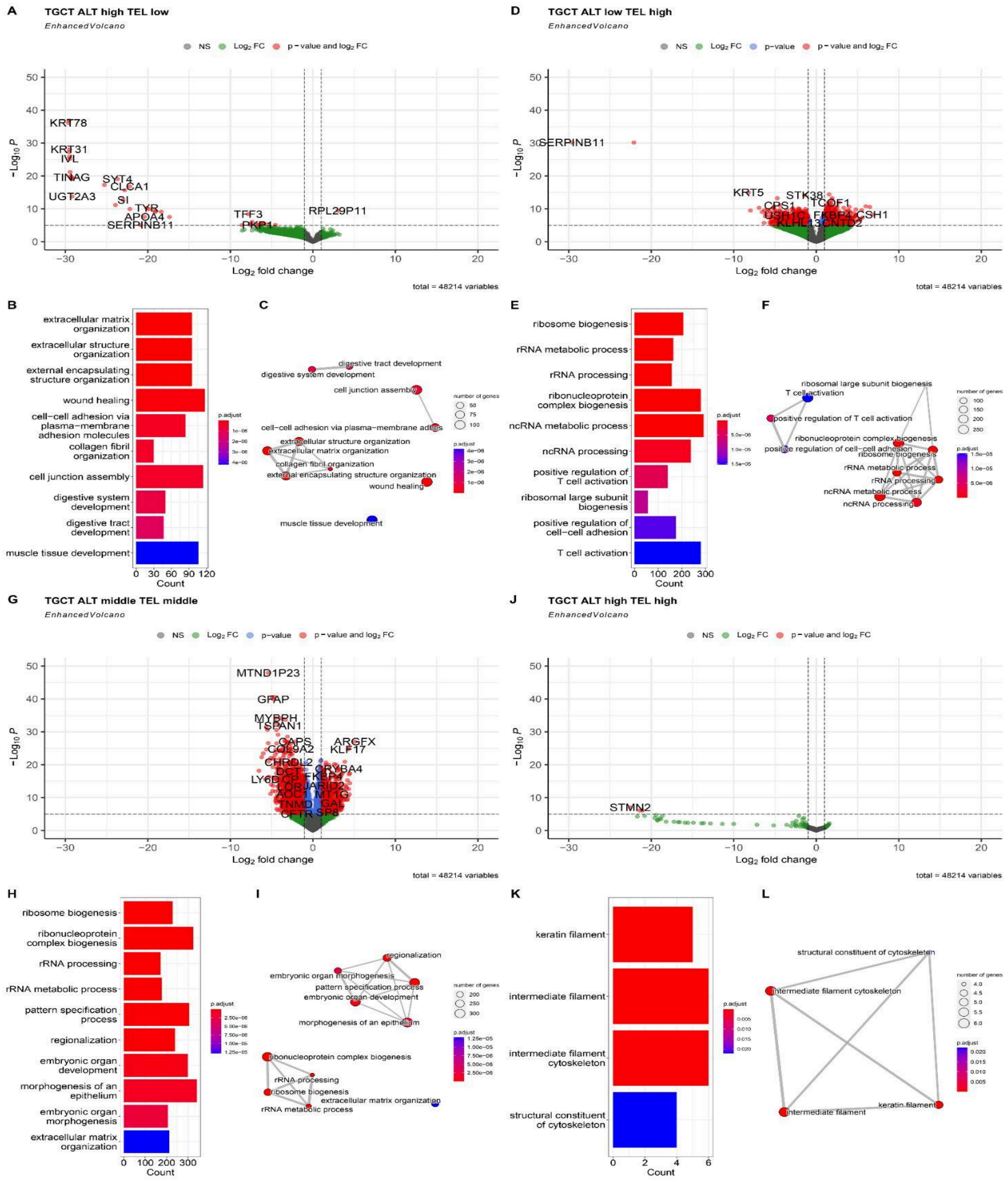

Figure S69. TGCT DEG results and GO over-represented terms. (**A**) Volcano plot of differentially expressed genes (DEGs) for ALT ^{high} TEL ^{low} phenotype. (**B**) Barplot displays the top GO terms by Adjusted p-value. (**C**) Enrichmap clusters the most significant (by padj) GO terms to visualize relationships between terms. (**D**) Volcano plot of differentially expressed genes (DEGs) for ALT ^{low} TEL ^{high} phenotype. (**E**) Barplot displays the top GO terms by Adjusted p-value. (**F**) Enrichmap clusters the most significant (by padj) GO terms to visualize relationships between terms. (**G**) Volcano plot of differentially expressed genes (DEGs) for ALT ^{middle} TEL ^{middle} phenotype. (**H**) Barplot displays the top GO terms by Adjusted p-value. (**I**) Enrichmap clusters the most significant (by padj) GO terms to visualize relationships between terms. (**J**) Volcano plot of differentially expressed genes (DEGs) for ALT ^{high} TEL^{high} phenotype. (**K**) Barplot displays the top GO terms by Adjusted p-value. (**L**) Enrichmap clusters the most significant (by padj) GO terms to visualize relationships between terms. Matched normal samples have been removed.

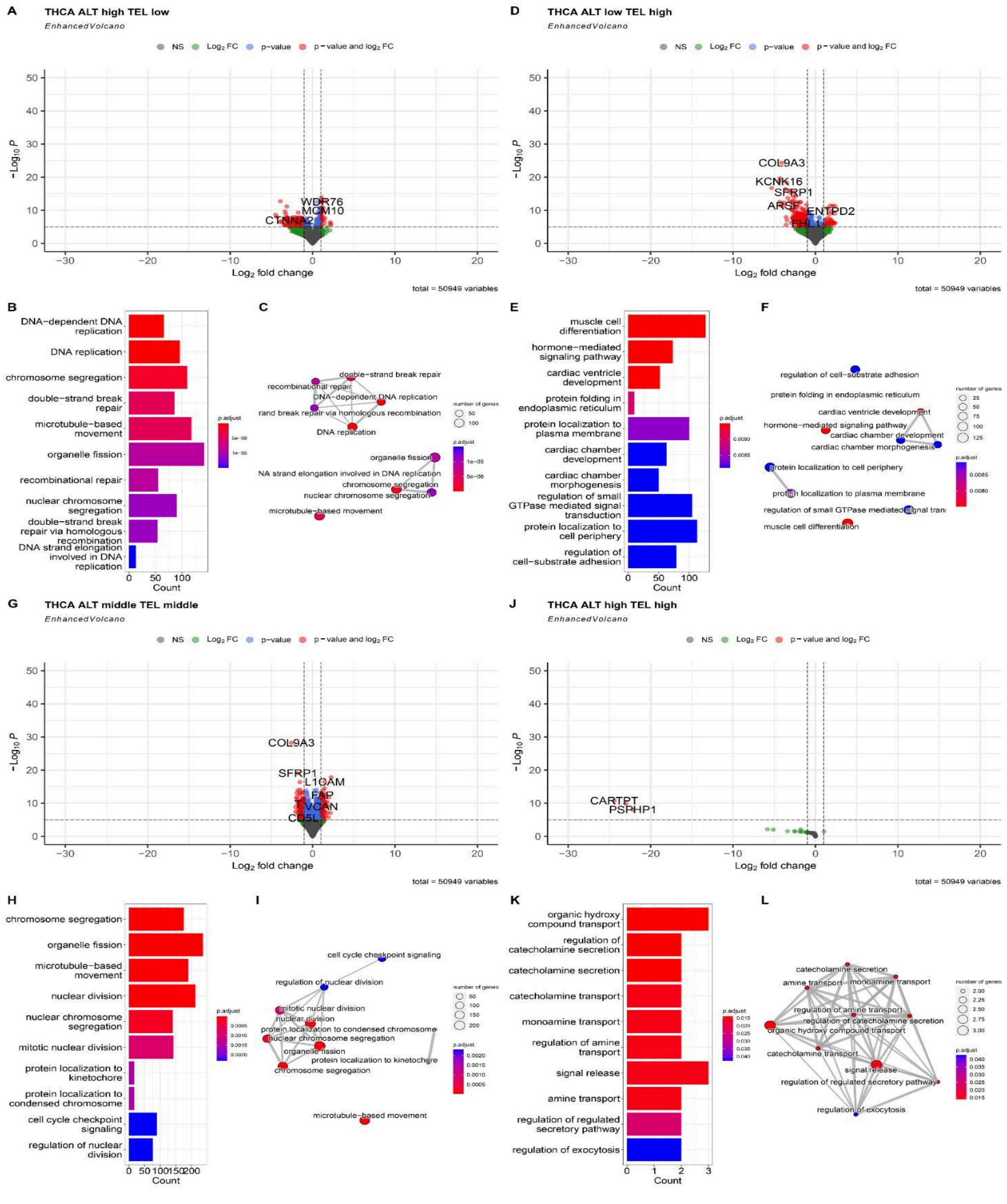

Figure S70. THCA DEG results and GO over-represented terms. (**A**) Volcano plot of differentially expressed genes (DEGs) for ALT high TEL low phenotype. (**B**) Barplot displays the top GO terms by Adjusted p-value. (**C**) Enrichmap clusters the most significant (by padj) GO terms to visualize relationships between terms. (**D**) Volcano plot of differentially expressed genes (DEGs) for ALT low TEL high phenotype. (**E**) Barplot displays the top GO terms by Adjusted p-value. (**F**) Enrichmap clusters the most significant (by padj) GO terms to visualize relationships between terms. (**G**) Volcano plot of differentially expressed genes (DEGs) for ALT middle TEL middle phenotype. (**H**) Barplot displays the top GO terms by Adjusted p-value. (**I**) Enrichmap clusters the most significant (by padj) GO terms to visualize relationships between terms. (**J**) Volcano plot of differentially expressed genes (DEGs) for ALT high TEL high phenotype. (**K**) Barplot displays the top GO terms by Adjusted p-value. (**L**) Enrichmap clusters the most significant (by padj) GO terms to visualize relationships between terms. Matched normal samples have been removed.

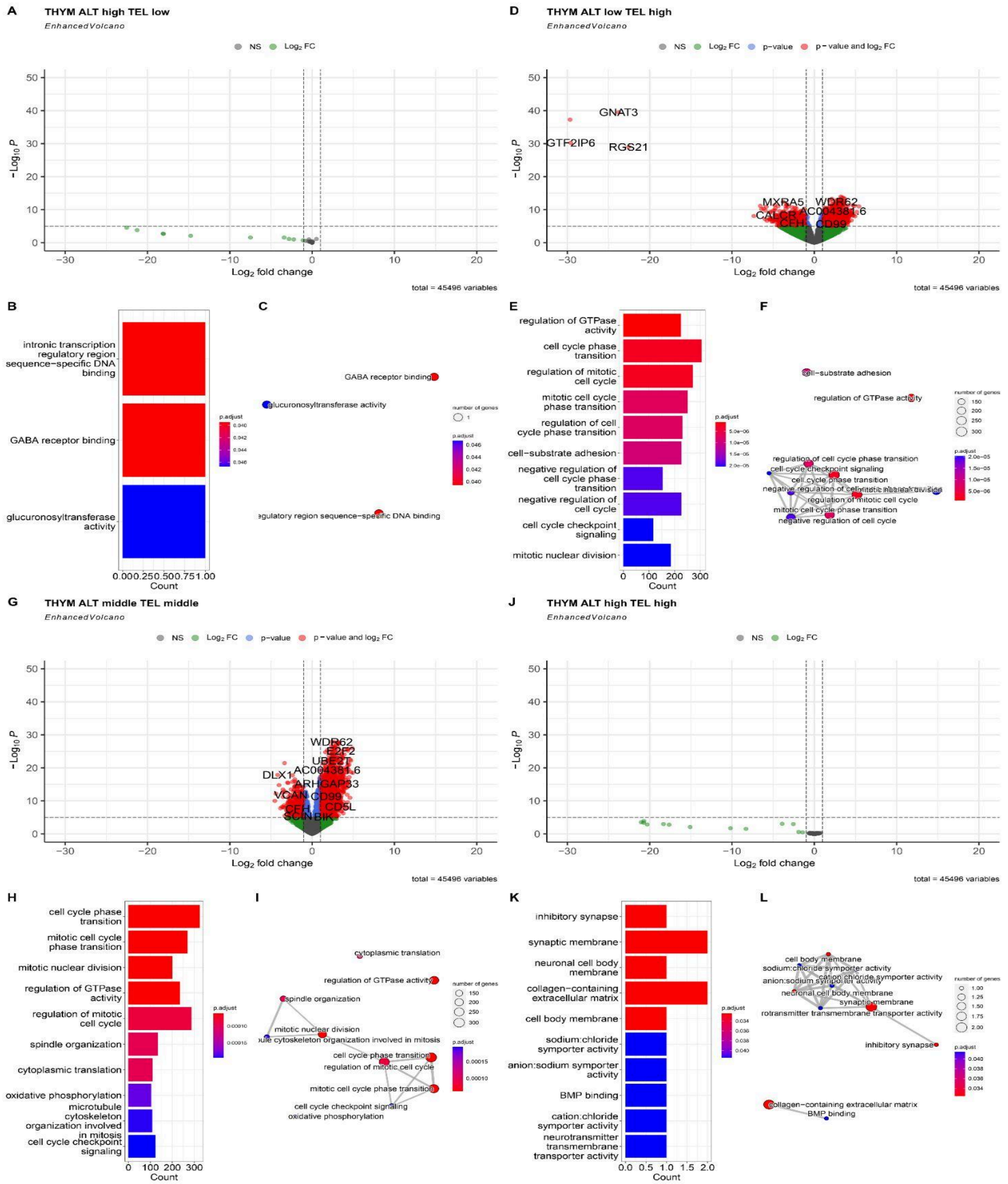

Figure S71. THYM DEG results and GO over-represented terms. (**A**) Volcano plot of differentially expressed genes (DEGs) for ALT ^{high} TEL ^{low} phenotype. (**B**) Barplot displays the top GO terms by Adjusted p-value. (**C**) Enrichmap clusters the most significant (by padj) GO terms to visualize relationships between terms. (**D**) Volcano plot of differentially expressed genes (DEGs) for ALT ^{low} TEL ^{high} phenotype. (**E**) Barplot displays the top GO terms by Adjusted p-value. (**F**) Enrichmap clusters the most significant (by padj) GO terms to visualize relationships between terms. (**G**) Volcano plot of differentially expressed genes (DEGs) for ALT ^{middle} TEL ^{middle} phenotype. (**H**) Barplot displays the top GO terms by Adjusted p-value. (**I**) Enrichmap clusters the most significant (by padj) GO terms to visualize relationships between terms. (**J**) Volcano plot of differentially expressed genes (DEGs) for ALT ^{high} TEL^{high} phenotype. (**K**) Barplot displays the top GO terms by Adjusted p-value. (**L**) Enrichmap clusters the most significant (by padj) GO terms to visualize relationships between terms. Matched normal samples have been removed.

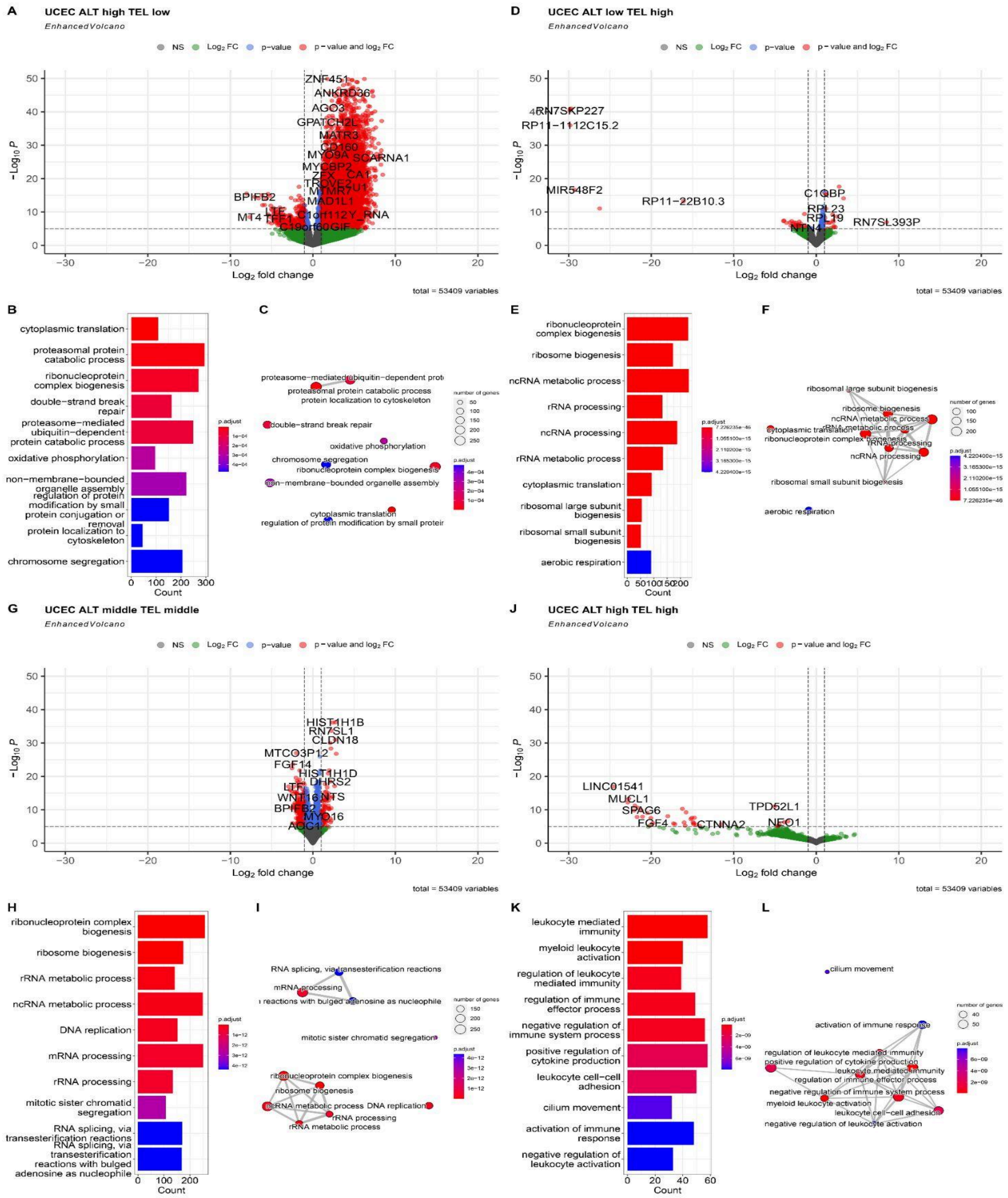

Figure S72. UCEC DEG results and GO over-represented terms. (**A**) Volcano plot of differentially expressed genes (DEGs) for ALT ^{high} TEL ^{low} phenotype. (**B**) Barplot displays the top GO terms by Adjusted p-value. (**C**) Enrichmap clusters the most significant (by padj) GO terms to visualize relationships between terms. (**D**) Volcano plot of differentially expressed genes (DEGs) for ALT ^{low} TEL ^{high} phenotype. (**E**) Barplot displays the top GO terms by Adjusted p-value. (**F**) Enrichmap clusters the most significant (by padj) GO terms to visualize relationships between terms. (**G**) Volcano plot of differentially expressed genes (DEGs) for ALT ^{middle} TEL ^{middle} phenotype. (**H**) Barplot displays the top GO terms by Adjusted p-value. (**I**) Enrichmap clusters the most significant (by padj) GO terms to visualize relationships between terms. (**J**) Volcano plot of differentially expressed genes (DEGs) for ALT ^{high} TEL^{high} phenotype. (**K**) Barplot displays the top GO terms by Adjusted p-value. (**L**) Enrichmap clusters the most significant (by padj) GO terms to visualize relationships between terms. Matched normal samples have been removed.

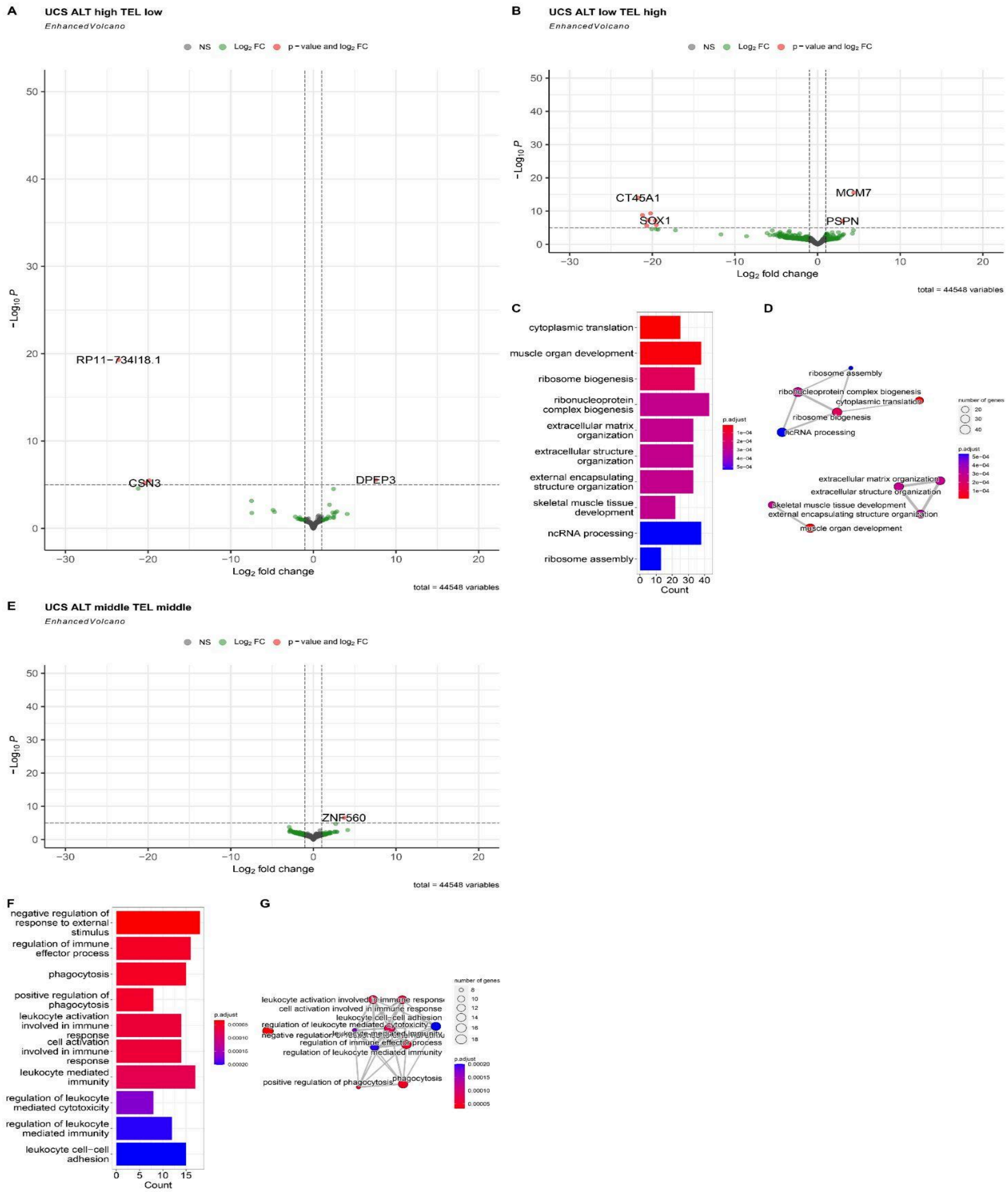

Figure S73. UCS DEG results and GO over-represented terms. (**A**) Volcano plot of differentially expressed genes (DEGs) for ALT high TEL low phenotype. Over-represented terms were missing. (**B**) Volcano plot of differentially expressed genes (DEGs) for ALT low TEL high phenotype. (**C**) Barplot displays the top GO terms by Adjusted p-value. (**D**) Enrichmap clusters the most significant (by padj) GO terms to visualize relationships between terms. (**E**) Volcano plot of differentially expressed genes (DEGs) for ALT middle TEL middle phenotype. (**F**) Barplot displays the top GO terms by Adjusted p-value. (**G**) Enrichmap clusters the most significant (by padj) GO terms to visualize relationships between terms. Matched normal samples have been removed.

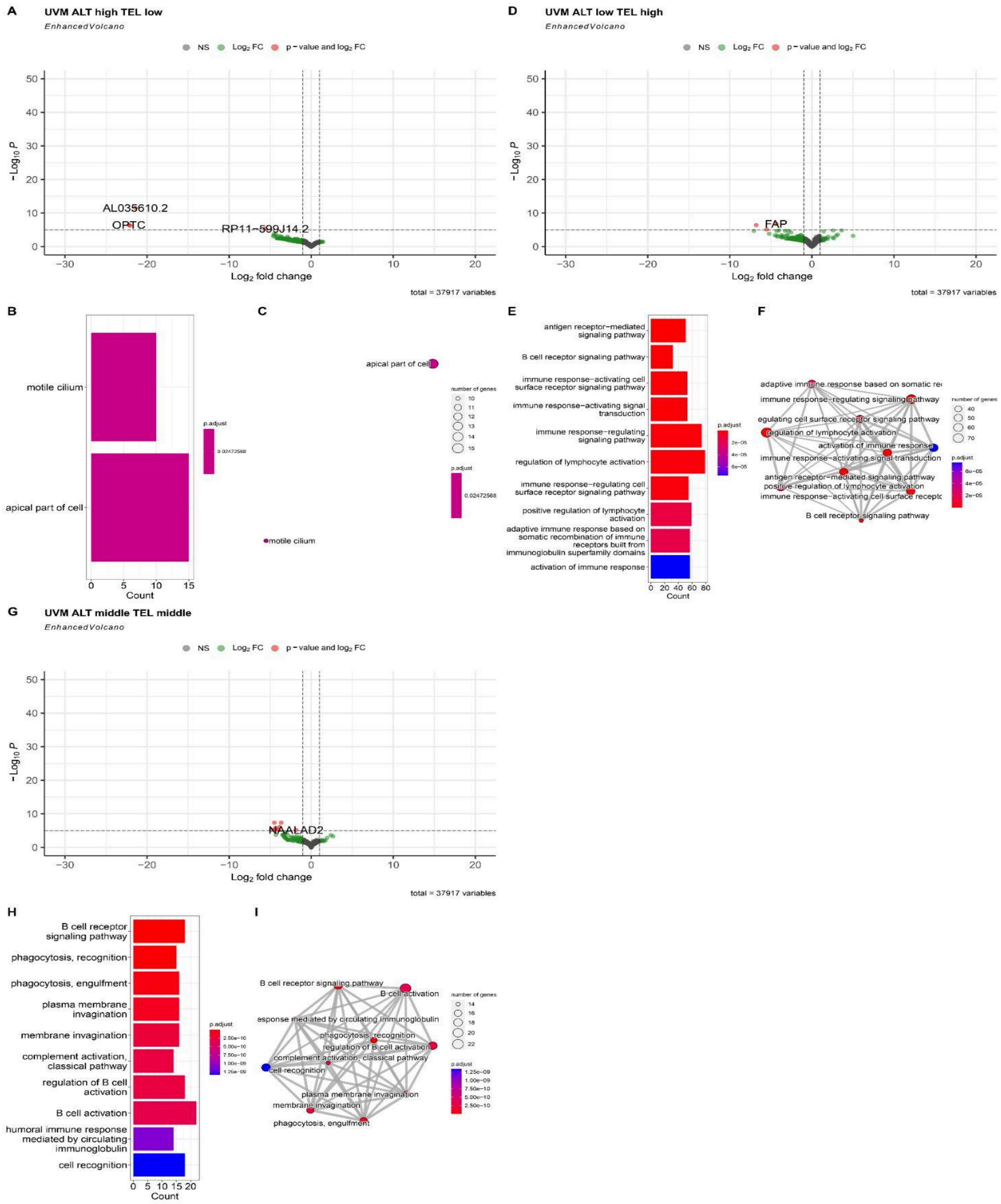

Figure S74. UVM DEG results and GO over-represented terms. (**A**) Volcano plot of differentially expressed genes (DEGs) for ALT ^{high} TEL ^{low} phenotype. (**B**) Barplot displays the top GO terms by Adjusted p-value. (**C**) Enrichmap clusters the most significant (by padj) GO terms to visualize relationships between terms. (**D**) Volcano plot of differentially expressed genes (DEGs) for ALT ^{low} TEL ^{high} phenotype. (**E**) Barplot displays the top GO terms by Adjusted p-value. (**F**) Enrichmap clusters the most significant (by padj) GO terms to visualize relationships between terms. (**G**) Volcano plot of differentially expressed genes (DEGs) for ALT ^{middle} TEL ^{middle} phenotype. (**H**) Barplot displays the top GO terms by Adjusted p-value.

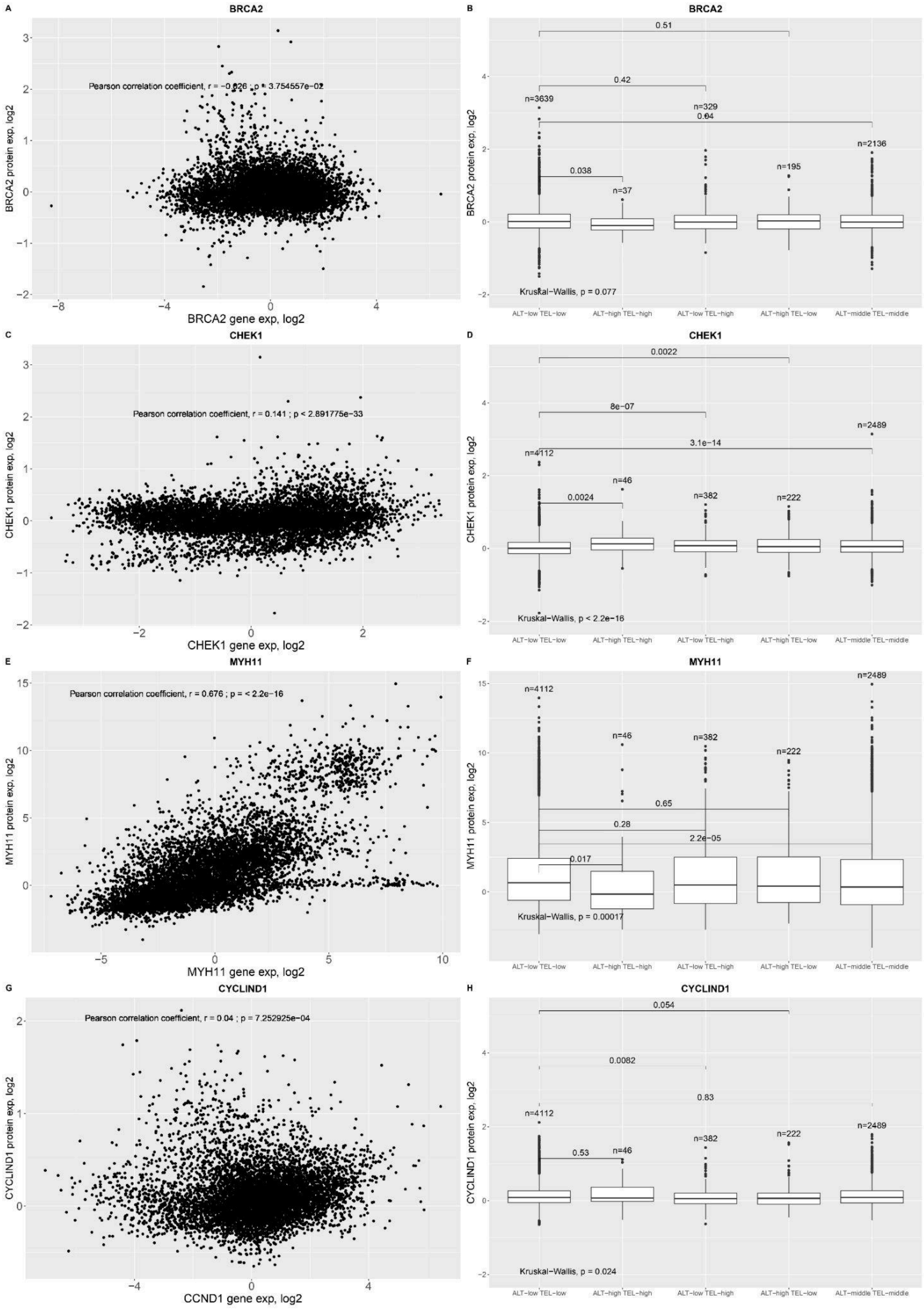
A
BRCA2
Pearson correlation coefficient, r = −0.026 ; p = 3.754557e−02
BRCA2 protein exp, log2
BRCA2 gene exp, log2
B
BRCA2
0.51
0.42
n=3639
n=329
0.64
n=2136
0.038
n=195
n=37
BRCA2 protein exp, log2
Kruskal−Wallis, p = 0.077
ALT−low TEL−low
ALT−high TEL−high
ALT−low TEL−high
ALT−high TEL−low
ALT−middle TEL−middle
C
CHEK1
Pearson correlation coefficient, r = 0.141 ; p < 2.891775e−33
CHEK1 protein exp, log2
CHEK1 gene exp, log2
D
CHEK1
0.0022
8e−07
n=2489
n=4112
3.1e−14
n=46
0.0024
n=382
n=222
CHEK1 protein exp, log2
Kruskal−Wallis, p < 2.2e−16
E
MYH11
Pearson correlation coefficient, r = 0.676 ; p = < 2.2e−16
MYH11 protein exp, log2
MYH11 gene exp, log2
F
MYH11
n=2489
n=4112
n=46
n=382
n=222
0.65
0.28
2.2e−05
0.017
MYH11 protein exp, log2
Kruskal−Wallis, p = 0.00017
G
CYCLIND1
Pearson correlation coefficient, r = 0.04 ; p = 7.252925e−04
CYCLIND1 protein exp, log2
CCND1 gene exp, log2
H
CYCLIND1
0.054
0.0082
0.83
n=4112
n=222
n=2489
n=382
0.53
n=46
CYCLIND1 protein exp, log2
Kruskal−Wallis, p = 0.024

Figure S75. Correlation plots of the relationship between protein expression versus RNA-seq gene expression, and proteome-level TMM phenotype activity plots for all cancer types. (**A**) and (**B**) BRCA2 gene. Statistical analysis for TMM phenotypes comparison used a Kruskal-Wallis test. (**C**) and (**D**) CHECK1 gene. Statistical analysis for TMM phenotypes comparison used a Kruskal-Wallis test. (**E**) and (**F**) MYH11 gene. Statistical analysis for TMM phenotypes comparison used a Kruskal-Wallis test. (**G**) and (**H**) CCND1 gene. Statistical analysis for TMM phenotypes comparison used a Kruskal-Wallis test.

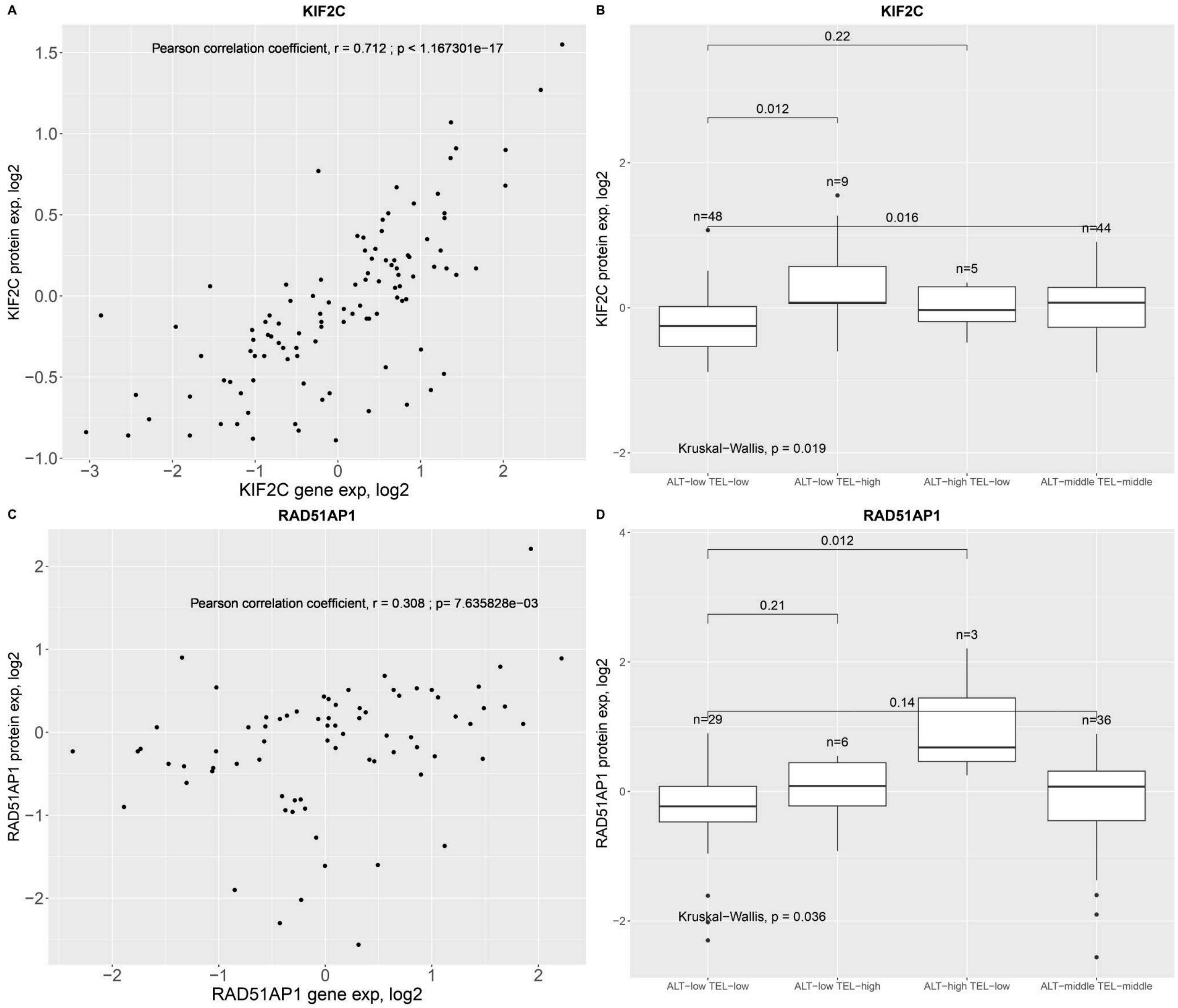

Figure S76. Correlation plots of the relationship between protein expression versus RNA-seq gene expression, and proteome-level TMM phenotype activity plots for BRCA. (**A**) and (**B**) KIF2C gene. Statistical analysis for TMM phenotypes comparison used a Kruskal-Wallis test. (**C**) and (**D**) RAD51AP1 gene. Statistical analysis for TMM phenotypes comparison used a Kruskal-Wallis test.
