# Supplementary material for: Pan-cancer analysis of telomere maintenance mechanisms
Source: J Biol Chem. 2024 May 18;300(6):107392. doi: 10.1016/j.jbc.2024.107392 (PMC11225560; doi:10.1016/j.jbc.2024.107392)
Supplement: Table S2 [file mmc2.docx]

**Table 1. TCGA Cancer types with the number of analyzed samples**

| **Cancer type** | **TCGA code** | **Number of Cancer Samples** | **Number of Normal Samples** | **Cancer type** | **TCGA code** | **Number of Cancer Samples** | **Number of Normal Samples** |
| --- | --- | --- | --- | --- | --- | --- | --- |
| **Adrenocortical carcinoma** | **ACC** | **79** | **NA*** | **Lung squamous cell carcinoma** | **LUSC** | **502** | **49** |
| **Bladder Urothelial Carcinoma** | **BLCA** | **412** | **19** | **Mesothelioma** | **MESO** | **87** | **NA*** |
| **Breast invasive carcinoma** | **BRCA** | **1127** | **99** | **Ovarian serous cystadenocarcinoma** | **OV** | **381** | **NA*** |
| **Cervical squamous cell carcinoma and endocervical adenocarcinoma** | **CESC** | **306** | **3** | **Pancreatic adenocarcinoma** | **PAAD** | **179** | **4** |
| **Cholangiocarcinoma** | **CHOL** | **35** | **9** | **Pheochromocytoma and Paraganglioma** | **PCPG** | **184** | **3** |
| **Colon adenocarcinoma** | **COAD** | **480** | **41** | **Prostate adenocarcinoma** | **PRAD** | **502** | **51** |
| **Lymphoid Neoplasm Diffuse Large B-cell Lymphoma** | **DLBC** | **48** | **NA*** | **Rectum adenocarcinoma** | **READ** | **167** | **10** |
| **Esophageal carcinoma** | **ESCA** | **163** | **11** | **Sarcoma** | **SARC** | **263** | **2** |
| **Glioblastoma multiforme** | **GBM** | **169** | **5** | **Skin Cutaneous Melanoma** | **SKCM** | **472** | **1** |
| **Head and Neck squamous cell carcinoma** | **HNSC** | **504** | **44** | **Stomach adenocarcinoma** | **STAD** | **375** | **32** |
| **Kidney Chromophobe** | **KICH** | **65** | **25** | **Testicular Germ Cell Tumors** | **TGCT** | **156** | **NA*** |
| **Kidney renal clear cell carcinoma** | **KIRC** | **541** | **72** | **Thymoma** | **THYM** | **120** | **2** |
| **Kidney renal papillary cell carcinoma** | **KIRP** | **291** | **32** | **Thyroid carcinoma** | **THCA** | **514** | **57** |
| **Acute Myeloid Leukemia** | **LAML** | **150** | **NA*** | **Uterine Carcinosarcoma** | **UCS** | **57** | **NA** |
| **Brain Lower Grade Glioma** | **LGG** | **532** | **NA*** | **Uterine Corpus Endometrial Carcinoma** | **UCEC** | **554** | **35** |
| **Liver hepatocellular carcinoma** | **LIHC** | **374** | **50** | **Uveal Melanoma** | **UVM** | **80** | **NA*** |
| **Lung adenocarcinoma** | **LUAD** | **540** | **58** |  |  |  |  |

*Note: "NA" indicates Not available data in the TCGA.
